# Spotlight on amino acid changing mutations in the JAK-STAT pathway: from disease-specific mutation to general mutation databases

**DOI:** 10.1038/s41598-025-90788-5

**Published:** 2025-02-20

**Authors:** Markus Hoffmann, Lothar Hennighausen

**Affiliations:** Laboratory of Genetics and Physiology, National Institute of Diabetes and Digestive and Kidney Diseases, Bethesda, MD 20892 USA

**Keywords:** Cancer, Genetics, Immunology, Molecular biology

## Abstract

The JAK-STAT pathway is central to cytokine signaling and controls normal physiology and disease. Aberrant activation via mutations that change amino acids in proteins of the pathway can result in diseases. While disease-centric databases like COSMIC catalog mutations in cancer, their prevalence in healthy populations remains underexplored. We systematically studied such mutations in the JAK-STAT genes by comparing COSMIC and the population-focused All of Us database. Our analysis revealed frequent mutations in all JAK and STAT domains, particularly among white females. We further identified three categories: Mutations uniquely found in All of Us that were associated with cancer in the literature but could not be found in COSMIC, underscoring COSMIC’s limitations. Mutations unique to COSMIC underline their potential as drivers of cancer due to their absence in the general population. Mutations present in both databases, e.g., JAK2^Val617Phe/V617F^ - widely recognized as a cancer driver in hematopoietic cells, but without disease associations in All of Us, raising the possibility that combinatorial SNPs might be responsible for disease development. These findings illustrate the complementarity of both databases for understanding mutation impacts and underscore the need for multi-mutation analyses to uncover genetic factors underlying complex diseases and advance personalized medicine.

**Supplementary Information:**

The online version contains supplementary material available at 10.1038/s41598-025-90788-5.

## Introduction

Single Nucleotide Polymorphisms (SNPs) are the most common type of genetic variation among people^1^. SNPs can be classified based on their DNA location and potential impact on the expression or function of one or several genes^2^. SNPs can play critical roles in gene regulation within non-coding regions by influencing elements such as promoters and enhancers^3–5^. Within coding regions, SNPs are divided into synonymous SNPs, which do not alter the amino acid sequence of the encoded protein but can affect translation speed, and nonsynonymous/missense SNPs, which result in an amino acid change and may affect protein folding and/or function^2^. SNPs in the JAK-STAT pathway—a crucial signaling cascade involved in immune responses and cancer development (Suppl. Figure 1^6–10^), – have been widely recognized as risk factors or causes of a multitude of diseases^11^.

Mutations in JAK and STAT proteins frequently result in a promiscuous activation of the JAK-STAT pathway, a characteristic feature of various hematological cancers. For example, the JAK2^Val617Phe^ (also called V617F) mutation is common in myeloproliferative neoplasms, occurring in roughly 90–95% of polycythemia vera (PV) patients and in 50–60% of those with essential thrombocythemia and primary myelofibrosis^12,13^. This mutation results in a gain-of-function effect, leading to increased signaling through the JAK-STAT pathway even in the absence of cytokine stimulation, thereby promoting cell proliferation and survival^14,15^. Similarly, mutations in JAK1 and JAK3 have been identified in T-cell acute lymphoblastic leukemia (T-ALL), where they contribute to the hyperactivation of the JAK-STAT signaling cascade^16,17^. Another example is the Pseudokinase domain mutation JAK1^Val666Gly^, which has been shown to impair JAK3 phosphorylation and interleukin-2 (IL-2) signaling, which is critical for T-cell proliferation^18^. Moreover, the role of the JAK-STAT pathway extends beyond hematological malignancies. Aberrant activation of this pathway has been implicated in various solid tumors and autoimmune diseases, indicating its broader relevance in human health^19,20^. For instance, mutations in the STAT3 gene have been associated with increased tumor invasiveness and poor prognosis in various cancers^21,22^. Additionally, STAT3 mutations are particularly prevalent in T-cell neoplasms, where they contribute to oncogenesis by promoting cell proliferation and survival. For instance, mutations in the SH2 domain of STAT3 can lead to constitutive activation, resulting in enhanced transcriptional activity of target genes involved in cell growth and survival^5,16,23^. In T-ALL, approximately 20–30% of patients harbor mutations in STAT3 or other components of the JAK-STAT pathway, which are associated with poor prognosis and increased disease aggressiveness^16,23^. Similarly, mutations in STAT5B have been implicated in various hematological malignancies, including acute myeloid leukemia (AML) and chronic lymphocytic leukemia (CLL). The constitutive activation of STAT5B can enhance the expression of anti-apoptotic proteins, thereby promoting cell survival and contributing to the malignant phenotype^24^. In Waldenström’s macroglobulinemia, for example, constitutive activation of STAT5A and STAT5B has been shown to regulate immunoglobulin secretion, further emphasizing the role of these proteins in B-cell malignancies^24^.

This literature, among many more published articles, shows that many missense mutations in the JAK and STAT genes are already known to cause disease. Most of the SNPs published literature are included in SNP databases, such as COSMIC^25,26^, gnomAD^27^, dbSNP^28^, and All of Us^29^. Each database has its unique focus and utility: COSMIC specializes in somatic mutations related to cancer, gnomAD offers a broad survey of human genetic diversity from various global populations, dbSNP provides a catalog of SNPs found in other databases, and All of Us focuses on capturing the diversity of the United States population with an emphasis on underrepresented groups.

However, the current literature lacks a systematic analysis of the frequency of missense mutations in specific domains of the JAK and STAT genes and the prevalence of these mutations across different ethnicities and sexes at birth in the general and mostly healthy population. Additionally, there is a gap in studies comparing SNPs in JAK and STAT genes between disease-specific databases like COSMIC and general population databases such as All of Us. Hence, we examine the prevalence of missense mutations in the COSMIC and All of Us databases representing the cancerous and general mostly healthy populations, respectively. We focus on the main components of this pathway: JAK1-3, TYK2, and STAT1-6 (including STAT5A and STAT5B). We first determine the general frequency of SNPs altering amino acids in these genes within the general population and compare more frequent SNPs (which we found in at least 20 individuals in All of Us) to their prevalence in the disease-centric COSMIC database. We then add what has been published about these SNPs in the literature and highlight inconsistencies between the literature and the All of Us database.

## Results and discussion

### Assessing the frequency of JAK and STAT missense mutations in the gene domains in the general population

**Fig. 1:**
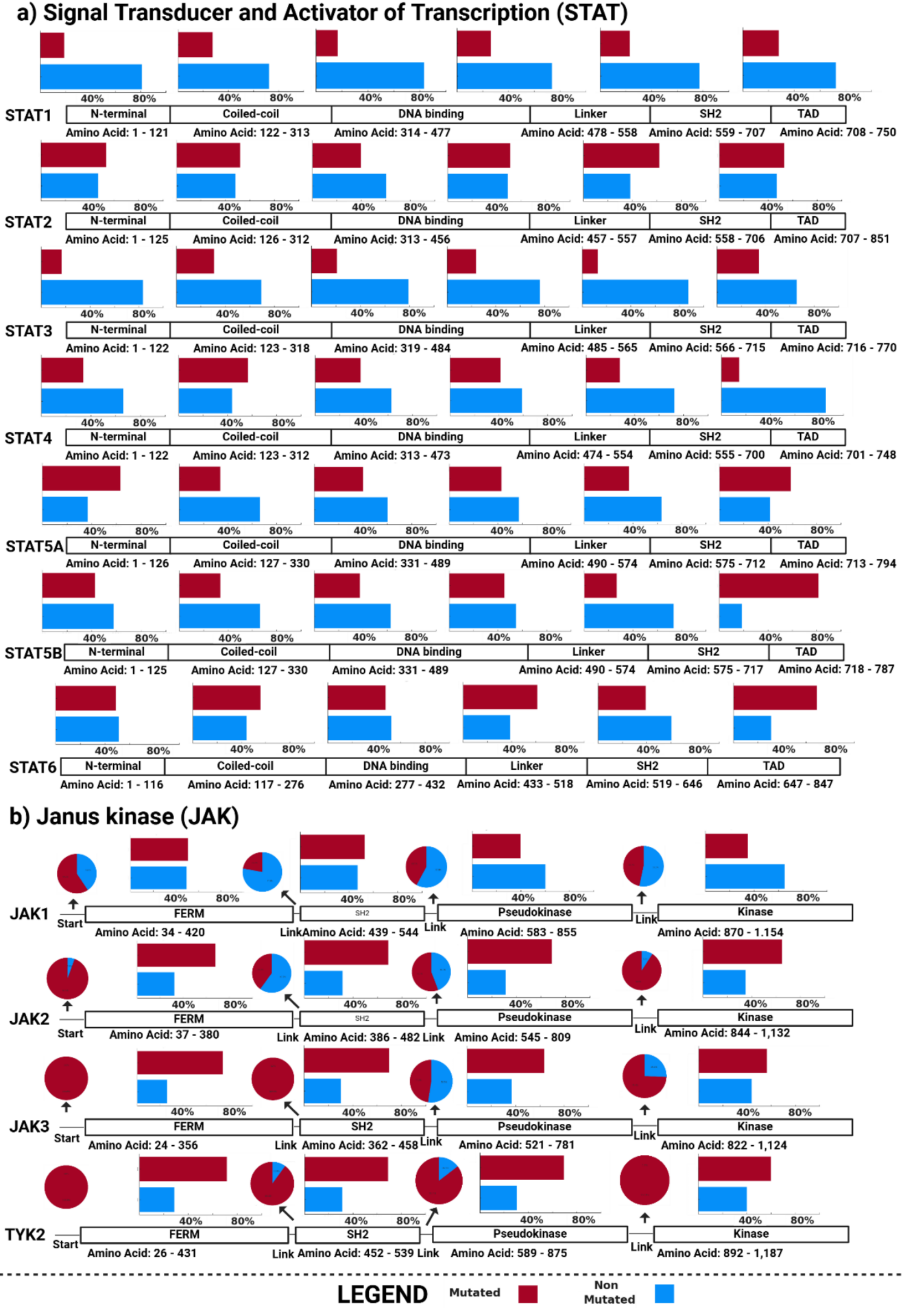
Distribution of mutations in JAK/STAT gene domains in the general population provided by All of Us. The JAK family (JAK1, JAK2, JAK3, and TYK2) and STAT family (STAT1–STAT6) are presented with pie charts and bar graphs that compare the proportion of mutated (red) versus non-mutated (blue) amino acid residues within each domain (i.e., N-terminal, Coiled-coil, DNA-binding, Linker, SH2, and TAD for the STAT family and FERM, SH2, Pseudokinase, Kinase, and links between those domains for the JAK family).

For this analysis, we visualize in Fig. 1 the percentage of how many amino acids per domain are mutated in at least one person in the All of Us database. Despite being important in immune response, we can observe that some gene domains of members within the JAK and STAT gene families are mostly heavily mutated (Fig. 1). The STAT genes have the following domains: (i) the N-terminal domain, (ii) the coiled-coil domain, (iii) the DNA-binding domain, (iv) the linker domain, (v) the SH2 domain, and (vi) the TAD domain. The N-terminal domain, essential for dimerization, seems to be lowly mutated. STAT6 has the lowest mutation rate in the N-terminal domain at 4%, followed by STAT2, STAT4, STAT5A, and STAT5B, each with 7%. STAT1, with a mutation rate of 11%, displays the highest degree of variation. In the coiled-coil domain, which facilitates protein-protein interactions, STAT6 again has the lowest mutation rate at 15%, followed by STAT2 at 16%, and STAT1, STAT3, and STAT4 at 18%, indicating a relatively consistent level of mutational flexibility across these proteins. More differences are observed in the DNA-binding domain, which is responsible for interacting with gene promoters. STAT4 has the lowest mutation rate at 27%, followed by STAT1 at 29% and STAT3 and STAT5B at 33%. STAT6 shows 34%, and STAT5A has the highest rate at 37%. The linker domain, connecting functional regions, presents less variability compared to the DNA binding domain. STAT5B has the lowest mutation rate at 3%, followed by STAT2 at 5%, STAT1 at 6%, STAT3 at 7%, STAT5A at 9%, and STAT6 at 10%. For the SH2 domain, which plays a crucial role in phosphorylation-dependent signaling, STAT5B shows the least mutational change at 24%, followed by STAT5A and STAT1, both at 26%, STAT6 at 29%, STAT4 at 30%, STAT3 at 32%, and STAT2 with the highest mutation rate of 34%, suggesting greater flexibility in phosphorylation engagement across the family. Lastly, the TAD, which regulates transcriptional activity, varies significantly across the STAT proteins. STAT1 has the lowest mutation rate at 29%, followed by STAT3 and STAT5B, both at 33%. STAT5A shows a mutation rate of 37%, while STAT6, with a rate of 42%, exhibits the most variation^30,31^. Overall, it seems that the SH2 and TAD domains of the STAT genes are more heavily mutated throughout the general mostly healthy population than the other domains^30–33^.

The JAK genes have the following domains: (i) the starter link, (ii) the FERM domain, (iii) the FERM link, (iv) the SH2 domain, (v) the SH2 link, (vi) the Pseudokinase, (vii) the Pseudokinase link, and (viii) the Kinase. The Starter link exhibits varying levels of mutation across the JAK family. JAK1 shows the lowest mutation rate at 2%, followed by TYK2 at 5% and JAK2 at 7%, while JAK3 has the highest, with 100% of residues affected. This wide variation suggests differing levels of flexibility and conservation in this domain across the JAK family. The FERM domain, critical for interacting with cytokine receptors, shows considerable variability. JAK1 displays the lowest mutation rate at 51%, followed by JAK2 at 65%. JAK3, with a mutation rate of 74%, and TYK2 at 100%, reflect a high degree of variation. The FERM link also exhibits distinct levels of mutation across the JAK family. JAK1 has the lowest mutation rate at 22%, followed by TYK2 at 47% and JAK2 at 66%. JAK3, with 100% of residues mutated, reflects the highest degree of mutational flexibility in this connecting region. In the SH2 domain, which is essential for recognizing and binding phosphorylated tyrosine residues, JAK1 shows the least variation, with a mutation rate of 40%, followed by TYK2 at 46% and JAK2 at 69%. JAK3, with 70%, exhibits the most variability in this domain, highlighting potential differences in phosphorylation-dependent signaling across the JAK family. The SH2 link domain demonstrates moderate levels of mutation across the JAK family. JAK1 and TYK2 exhibit the lowest rates at 11%, followed by JAK2 at 18%, while JAK3 shows the highest mutation rate at 100%, indicating significant flexibility in this region, particularly in JAK3. The Pseudokinase domain, which regulates the kinase activity, shows high mutation rates across the JAK family. JAK1 displays the lowest rate at 40%, while JAK2 shows 47%, and JAK3 reaches 45%. TYK2 demonstrates the highest mutation rate in this domain at 57%. In the Pseudokinase linker, JAK2 has the lowest mutation rate at 1%, followed by TYK2 at 18%, JAK1 at 47%, and JAK3 at 100%. Lastly, the kinase domain, which is vital for catalytic activity, exhibits substantial differences in mutation rates. JAK1 has the lowest rate at 35%, while JAK3 and TYK2 show similar rates at 39% and 40%, respectively. JAK2, however, demonstrates the highest rate at 65%, reflecting a broader range of variation in the kinase domain across the family^30–33^.

It seems that variations in the JAK domains generally occur more often than in STAT domains. This data suggests that while certain domains are more prone to mutation, no JAK and STAT gene family domain is fully conserved in the general population. These variations may subtly influence how individuals respond to signaling and immune challenges, even in the absence of disease. The findings from the All of Us database challenge the assumption that essential domains in both the JAK and STAT families are highly conserved throughout the healthy population since a significant number of mutation rates are observed across key functional domains.

**Fig. 2:**
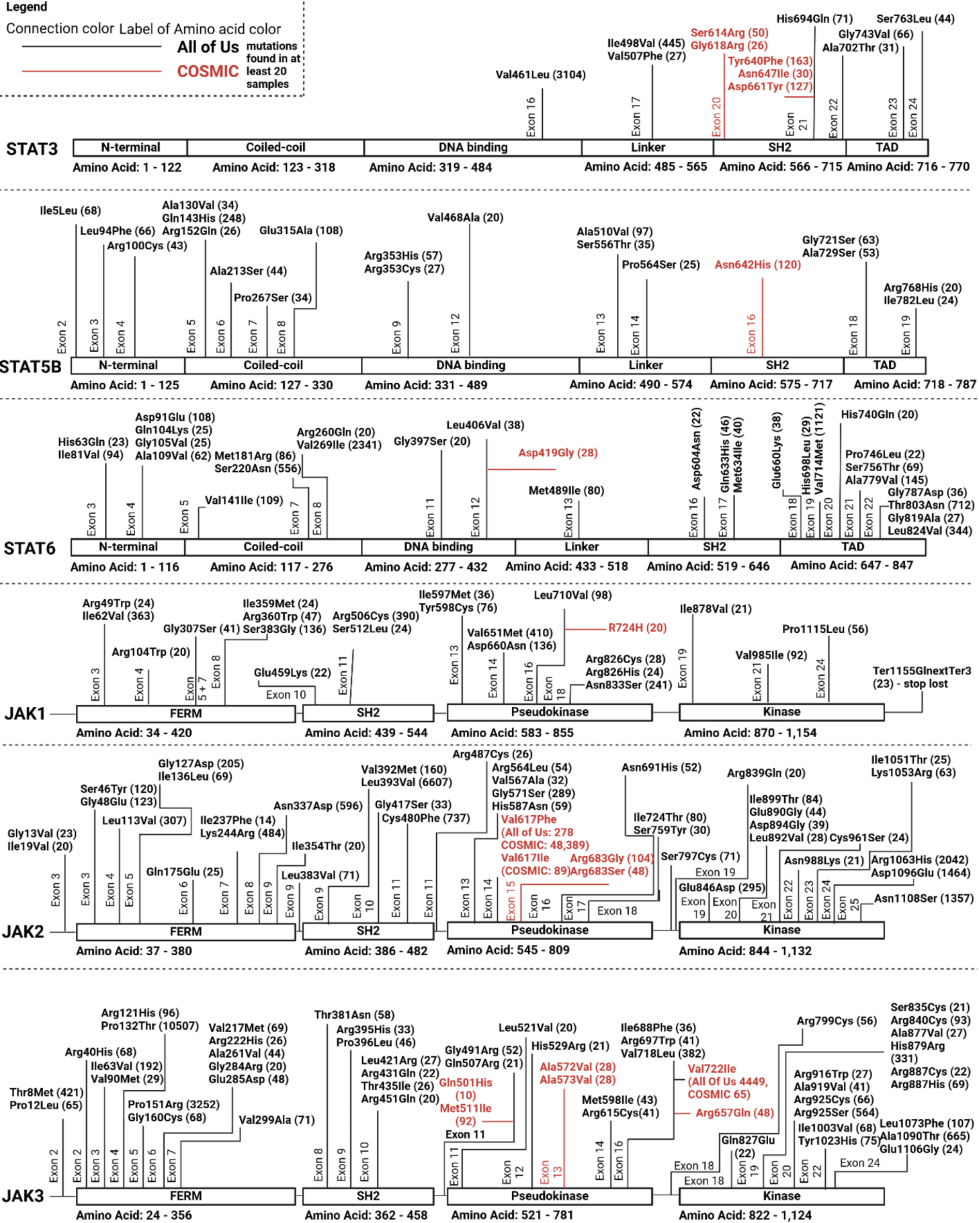
Missense mutations found in more than 20 samples in All of Us or COSMIC for STAT5B, STAT6, JAK1, JAK2, and JAK3. STAT1, STAT2, STAT3, STAT4, STAT5A, and TYK2 can be found in Suppl. Figures 2–4. If the amino acid change is labeled in red color then it exists in at least 20 individuals in COSMIC but not in All of Us. If the amino acid change is labeled in black color then it exists in at least 20 individuals in All of Us. We labeled it with COSMIC and All of Us numbers, if an amino acid change exists in both databases.

### Comparing missense single nucleotide variants in the JAK/STAT genes that were identified in All of Us or COSMIC to what is known in the literature and how they are associated with disease in All of Us

We examined the All of Us database for missense mutations that occur in at least 20 individuals within the JAK and STAT gene families and evaluated whether these mutations are associated with any diseases based on the available data in All of Us (Fig. 2). Additionally, we conducted a literature review for each of these mutations. We first discuss the SNPs identified in general, focusing on their associations with sex at birth and ethnicity. Next, we investigate some SNPs that are predominant in the Asian-American population. Then, we explored the SNPs found in both All of Us and COSMIC, reviewing the existing literature on these variants. Following this, we assessed the SNPs that were present in All of Us but absent in COSMIC and vice versa.

Suppl. Table 1 shows that mutations in members of the JAK-STAT pathway predominantly occur in females. STAT1 mutations are most frequently found in Black and Hispanic females. STAT2 and STAT3 also show a significant representation of Black and Hispanic individuals but include a substantial number of mutations observed in white females, especially at the end of the protein sequence. On the other hand, STAT4 mutations are primarily observed in Black females. Both STAT5A and STAT5B exhibit similar trends, with mutations predominantly affecting females in White and Black populations. STAT6 mutations, while also more frequent in females, show a strong presence in White populations. For the JAK family, JAK1 and JAK2 mutations also trend toward female prevalence, particularly in White populations. JAK3 exhibits a similar pattern of female predominance across various ethnicities. TYK2 mutations follow this overarching trend, with a notable prevalence among Hispanic and White females. In the All of Us database, none of these SNPs could be significantly associated with a disease.

While the analysis highlights significant representation among Black, Hispanic, and White populations, we recognize the presence of mutations affecting Asian populations as well. A few JAK-STAT pathway mutations have been observed in Asian females and males, mainly with no health condition associated with the All of Us database. A total of 205 have been identified in All of Us with rs56118985 (JAK2^Gly127Asp^), most of them Asian females. In the literature, this missense mutation is associated with Philadelphia-negative myeloproliferative neoplasms (Ph-MPN), a group of cancers that cause the body to produce too many blood cells^34^. The authors highlight that rs56118985 co-occurs exclusively with rs77375493 (polycythemia vera) in their patients. Additionally, Motegi et al. showed that the variant rs201917359 (TYK2^Arg231Trp^) was characterized as a gain-of-function mutation in the Japanese population, which could enhance TYK2 signaling and contribute to the pathogenesis of Rheumatoid Arthritis^35^. In the study by Nemoto et al. (2018), the SNP rs201917359 in the TYK2 gene was linked to primary immunodeficiency in two siblings, characterized by T-cell lymphopenia^36^. In the COSMIC database, neither rs56118985 nor rs201917359 was identified in 20 patients or more, and none of the individuals in the All of Us database was diagnosed with any of the diseases mentioned in the literature.

Next, we investigated whether some variants were identified in COSMIC and All of Us. The most prominent example is rs77375493 (polycythemia vera, JAK2^Val617Phe^). rs77375493 has been identified in 48,389 individuals in COSMIC and 278 individuals, from which 79% are 65 years or older, in All of Us. This mutation has been widely described as a risk factor in literature^37–51^. From the numbers in COSMIC, we can assume that it is directly related to cancer. However, upon closer look into All of Us, we cannot find any cancer diagnosis for those with rs77375493, even though most of the individuals are 65 years or older, and we would expect them to develop the disease. Similar to the previous findings, we also analyzed rs3213409 (JAK3^Val722Ile^, germline, also reported as somatic in a few solid tumors^52^), present in the All of Us and COSMIC databases. rs3213409 has been identified in 48 individuals in COSMIC and 4449 individuals within All of Us, predominantly affecting white females. However, no disease association is recorded in the All of Us database. In contrast, the literature has linked rs3213409 to several cancer types, including acute lymphoblastic leukemia^52–57^. These observations could be skewed since the individuals in All of Us who have rs77375493 or rs3213409 could be diagnosed with the disease after submitting their data.

We also found some further variations associated with various cancers in the literature that were not present in the COSMIC database but were present in the All of Us database. rs3212723 (JAK3^Pro132Thr^), a variation that was identified in 10,507 individuals (predominantly Black females) in All of Us but not in COSMIC, was associated with acute megakaryoblastic leukemia^58,59^, head and neck cancer^60^, and Ameloblastoma^61^ in the literature. In All of Us, we can only identify that the individuals suffer from essential hypertension and chest pain in large numbers. We also identified the SNP rs139504737 (JAK2^Gly571Ser^) in the All of Us database, where it was found in 289 individuals, predominantly among Hispanic females. However, no corresponding disease association was noted in the database. In the literature, rs139504737 has been linked to various malignancies, including acute lymphoblastic leukemia, myeloproliferative neoplasms, and thrombocythemia, suggesting a potential role in hematological disorders​ despite not being identified in large numbers in COSMIC^62–65^. rs372254348 (JAK2^Ile724Thr^) is present in the All of Us database but lacks cancer associations within the database. This SNP has been documented in the literature as being associated with myeloproliferative neoplasms, including conditions such as polycythemia vera, essential thrombocythemia, and primary myelofibrosis^66^. We observed rs142269166 (TYK2^Asn1108Ser^) in the All of Us database, where it was found in 1,357 individuals, primarily among white females, without any recorded disease associations. However, this SNP has been extensively studied in the literature, linking it to various myeloproliferative neoplasms, including polycythemia vera and myelofibrosis, which have been implicated in the transformation of myeloproliferative neoplasms into acute myeloid leukemia^43,67,68^. We detected rs200077579 (JAK3^Arg840Cys^) in the All of Us database but not in COSMIC, where it appears without any associated cancer diagnoses. This variant has been documented in the literature as a heterozygous mutation linked to Cytotoxic T Lymphocyte Antigen-4-Dependent Immune Dysregulation Syndrome^69^. rs201335603 (TYK2^Gly761Val^) is present in the All of Us database but lacks an associated disease diagnosis. This SNP has been implicated in the literature concerning various hematologic malignancies, particularly its association with acute lymphoblastic leukemia (ALL) and other cancers. Specifically, germline activating mutations in the TYK2 gene, including this SNP, have been linked to increased susceptibility to ALL, indicating a potential role in oncogenesis; however, none of the 66 individuals in All of Us seem to have any of the conditions^70,71^. The variant rs141331848 (STAT4^Thr446Ile^, 316 individuals in All of Us) has been documented in the literature as potentially linked to classic Kaposi sarcoma (cKS), with evidence suggesting its role in genetic predisposition. Specifically, this SNP was found in a Finnish family with affected individuals, indicating a possible association with the disease​^72^. We also noticed rs41316003 (JAK2^Arg1063His^), which has been documented in All of Us with 2,042 individuals. However, no associated disease diagnoses are present in the database. In the literature, this variant has been linked to several conditions, including myeloproliferative neoplasms and familial ischemic stroke^44,73–75^. None of these SNPs were associated with the diseases described in the literature in All of Us.

We reverse the search to investigate the SNPs that were found in COSMIC but not in All of Us. rs2081548277 (STAT3^Gly618Arg^) was identified in 21 samples in COSMIC but not in All of Us. rs2081548277 is associated with various hematological and lymphoid malignancies, particularly T-cell lymphomas. It is categorized as a gain-of-function (GOF) mutation that increases STAT3 activation, promoting cell proliferation and contributing to disease pathology^76–78^. In the context of large granular lymphocyte leukemia (LGLL), gain-of-function mutations in STAT3, including this specific variant, have been linked to aberrant cytokine signaling, specifically involving IL-6 and IL-15, and the upregulation of epigenetic regulators such as DNMT1 and EZH2. These mutations contribute to global hypermethylation, increased oxidative stress, and a proliferative advantage in affected cells^76–78^. The SNP rs770986654 (STAT3^Asn647Ile^) has been identified in 30 individuals in COSMIC but none in All of Us. In the literature, this mutation is associated with large granular lymphocyte (LGL) leukemia. rs770986654 is described as a gain-of-function mutation, leading to enhanced STAT3 activation. In the context of T-cell large granular lymphocytic leukemia, these gain-of-function mutations promote the survival and proliferation of leukemic T cells by upregulating cytokine signaling pathways, particularly involving interleukin-6 (IL-6) and interleukin-15 (IL-15). Moreover, such mutations are linked to increased activity of epigenetic regulators, contributing to the dysregulated gene expression observed in the disease^78–81^. The SNP rs747639500 (STAT3^Asp661Tyr^) is associated with large granular lymphocyte (LGL) leukemia in literature. This mutation has been identified in COSMIC in 127 individuals and is classified as a somatic gain-of-function mutation in the STAT3 gene^81–84^. The SNP rs938448224 (STAT5B^Asn642His^) has been identified in 120 individuals in COSMIC but not in All of Us. rs938448224, a gain-of-function mutation of STAT5B, has been implicated in several hematological disorders, particularly chronic myeloid neoplasms and eosinophilia. The mutation enhances STAT5B signaling, promoting abnormal cell proliferation and survival by increasing cytokine signaling^85–87^. The SNP rs1057519721 (JAK2^Arg683Gly^) has been detected in 104 individuals in COSMIC, though it was not found in the All of Us database. rs1057519721, a GOF mutation in JAK2, has been implicated in hematological malignancies, particularly ALL. The mutation enhances JAK2 signaling, promoting abnormal cell proliferation and survival by dysregulating cytokine signaling pathways, contributing to leukemic progression^88–92^. The SNP rs121913504 (JAK3^Ala572Val^) has been identified in 28 individuals in COSMIC. This variant has been implicated in various hematologic malignancies, including T-cell malignancies, particularly JAK3-mutation-positive leukemia. It is a gain-of-function mutation that enhances the signaling pathway of JAK3, contributing to abnormal cell proliferation and survival through dysregulated cytokine signaling. The mutation impacts downstream epigenetic regulators and cytokine-mediated pathways, further exacerbating disease progression^93–97^. The SNP rs2147686240 (JAK3^Ala573Val^) has been identified in 28 individuals in COSMIC. It is a well-known gain-of-function mutation in JAK3, located in the Pseudokinase domain. It has been associated with various hematological malignancies, particularly natural killer/T-cell lymphoma (NKTCL). The mutation enhances JAK3-mediated STAT5 activation, leading to cytokine-independent cell growth and contributing to the pathogenesis of NKTCL by promoting uncontrolled proliferation and survival of malignant cells^53,98–101^. The SNP rs758959409 (JAK3^Arg657Gln^) was identified in 48 individuals in COSMIC but not in All of Us. This variant is involved in hematological malignancies, particularly in Down syndrome-related acute megakaryoblastic leukemia (AMKL) and transient myeloproliferative disorder (TMD). Mutations like this promote constitutive activation of JAK3, leading to enhanced signaling through the JAK-STAT pathway, thereby driving abnormal cell proliferation and survival in affected cells^101,102^. These mutations seem to be promising candidates for disease driver mutations due to their absence in the All of Us database.

Finally, we identified one SNP (rs369530676, TYK2^Arg118Gln^) that is exclusive to the All of Us database and is found in 34 individuals. A notable majority of individuals carrying this variant are diagnosed with Type 2 Diabetes. This SNP has not been discussed in the literature yet and might represent a suitable candidate for future experimental investigation.

### Limitations and considerations

The JAK-STAT signaling pathway is known for its evolutionary conservation across species, which underscores its fundamental role in cellular communication and immune responses^103,104^. The analysis of missense mutations within the JAK-STAT pathway in the All of Us database reveals a first sight into a complex landscape of genetic variations within the mostly healthy population, which we found in every domain of the JAK and STAT proteins. However, most of the identified SNPs could not be associated with any disease in the All of Us database. Our findings indicate that while certain domains of the JAK and STAT proteins exhibit higher mutation rates, no domain of any member of the JAK or STAT gene family is entirely conserved across the U.S. population. However, these mutations could subtly affect immune responses and signaling pathways among individuals. The presence of mutations in critical functional domains raises the possibility that they may not universally lead to deleterious effects but could instead contribute to population-level genetic diversity^105–107^.

The analysis of SNPs within the JAK-STAT pathway in the All of Us database reveals a notable predominance of these genetic variations in white females. This observation raises important considerations regarding the implications of ethnic and sex-based disparities in genetic susceptibility to diseases associated with the JAK-STAT signaling pathway, particularly hematological malignancies, and autoimmune disorders, which are also more prevalent in females^108^, highlighting a potential association for future studies. Previous studies have indicated that certain genetic variants exhibit differential frequencies across ethnic groups, which can impact disease susceptibility and treatment responses^109,110^.

Our investigation of missense SNPs in the JAK-STAT pathway, using both the All of Us and COSMIC databases, reveals discrepancies in mutation representation and disease associations. The SNPs can be categorized into three groups: those found in both databases, those unique to All of Us, and those unique to COSMIC. Each category provides insight into the limitations of current genetic data and its interpretation.

An important consideration is the concept of clonal hematopoiesis (CH), increasingly recognized as a premalignant condition in which somatic mutations—including those in genes such as JAK2, JAK3, and STAT3—may be detected in otherwise healthy individuals. The 5th edition of the World Health Organization (WHO) Classification of Haematolymphoid Tumours underscores the frequent occurrence of these mutations in CH and their clinical significance^111^. In particular, JAK2^Val617Phe^ is well-established as the main driver in myeloproliferative neoplasms (MPNs). However, its detection in asymptomatic individuals may reflect clonal hematopoiesis of indeterminate potential (CHIP) and not an overt MPN. A fraction of carriers eventually develop MPN, influenced by factors such as inflammation, additional mutations^112^, and an increased allelic burden^113^. Similarly, JAK3^Val722Ile^ can appear as a germline variant in seemingly healthy individuals yet has been reported as a somatic change in certain solid tumors. These observations could help explain why recognized cancer driver mutations appear in the general population database (All of Us).

The next category, mutations found only in All of Us, such as rs56118985 and rs139504737, raises intriguing questions. These SNPs, particularly prevalent in certain ethnic populations, are associated with malignancies in the literature but show no corresponding disease diagnoses in All of Us and were not discovered in COSMIC. This suggests either underdiagnosis or that these mutations have not yet manifested as a disease in these individuals at the time of the sampling. It emphasizes the importance of including diverse populations in genetic studies to capture variations that may not be reflected in cancer-centric databases like COSMIC that, in general, overrepresent European ancestry^114,115^.

Mutations like rs2081548277 and rs938448224, found only in COSMIC, highlight COSMICs value. These variants associated with cancer are absent in the All of Us database, likely due to fewer people with a cancer diagnosis. These SNPs might represent important driver mutations since they cannot be found in the general population. COSMIC emphasizes disease endpoints, particularly malignancies, while All of Us includes individuals earlier in their disease progression or who may never develop cancer. These observations highlight the complementary strengths and limitations of different genetic databases. This showcases that considering both disease-focused and general population databases when selecting SNPs for investigation can provide a more comprehensive understanding of their potential significance in disease.

Ultimately, the well-studied JAK2^Val617Phe^ mutation is present in both databases and widely considered as a driver. It shows no disease associations in All of Us and raises the possibility that some mutations may require additional mutations for disease development. Moreover, the identification of novel mutations exclusive to the All of Us database, such as rs369530676 in TYK2, which correlates strongly with Type 2 Diabetes, suggests that the JAK-STAT pathway may play a broader role in metabolic diseases beyond its established involvement in cancer^116,117^.

Moreover, it is increasingly evident that most complex diseases might not be explained by a single SNP but rather by a combination of SNPs working together. Tools like NeEDL allow us to interpret the statistical significance of these combinations, revealing that with 3–7 SNPs, we can achieve a higher predictive score for complex heritable diseases like Rheumatoid Arthritis, Diabetes, diabetes, or Alzheimer’s disease^118–120^. This insight suggests that a similar approach could be applied to cancer biology, where multiple incidental SNPs might collectively serve as a root cause of cancer^121–123^.

## Conclusion

In conclusion, this study underscores the need for integrative approaches that combine population-level data with disease-focused resources. While COSMIC provides critical insight into cancer-associated mutations, All of Us expands the scope by capturing genetic diversity across the general, mostly healthy, population and underrepresented groups. The discrepancies between disease-specific and general population databases underscore the importance of integrating genetic data from various sources to enhance our understanding of the role of these mutations in disease pathogenesis. We showed that neither database alone is sufficient to investigate the impact of genetic mutations on disease. In most cases, we believe that a single SNP is insufficient to drive the onset of complex diseases. Hence, we plan to expand our future research into multi-SNP analyses and integrate diverse data sources to improve the prediction of important genetic factors in complex diseases. By combining the strengths of multiple databases with different objectives and integrating multi-SNP analyses, we can unlock new insights into complex diseases and drive advancements in personalized genomic medicine.

## Materials and methods

### All of us Data Explorer

The All of Us Research Program is a national initiative aimed at collecting and analyzing health data from a diverse cohort of participants, with a particular focus on underrepresented populations in biomedical research. For this study, we accessed genetic data **(All of Us Controlled Tier Dataset v7**,** which includes 413**,**000 participants)** through the All of Us Data Browser (https://databrowser.researchallofus.org/), which allows users to explore aggregated genomic data, including single nucleotide polymorphisms (SNPs) and their associations with demographic variables such as ethnicity, sex at birth, and disease diagnosis. We examined missense mutations within the JAK-STAT gene families using this browser, focusing on SNPs that alter amino acid sequences and their prevalence across different demographic groups. We also explored the associations between these variants and known diseases within the All of Us dataset. All retrieved data adhered to the ethical standards of the program, which anonymizes participant information to protect privacy. The genetic variants of interest were filtered based on their mutation type, and we analyzed the frequency of these mutations in the general population. We further stratified our analysis by sex at birth and ethnicity to investigate population-specific variations in mutation prevalence.

We used all missense mutations that were found in at least 1 participant in the section *“Assessing the frequency of JAK and STAT domain missense mutations in the general population”.* For the closer analysis in the section “*Comparing missense single nucleotide variants in the JAK/STAT genes that were identified in All of Us or COSMIC to what is known in the literature and how they are associated with disease in All of Us*”, we used all missense mutations that were found in at least 20 participants due to the All of Us database policy.

### COSMIC (Catalogue of Somatic Mutations in Cancer)

The Catalogue of Somatic Mutations in Cancer (COSMIC) database (https://cancer.sanger.ac.uk/cosmic) is a comprehensive resource documenting somatic mutations observed in cancer. We used COSMIC **(COSMIC v100)** to examine the prevalence of missense mutations in the JAK-STAT pathway, specifically focusing on mutations that have been identified in cancer samples. COSMIC provides detailed information about the mutational spectrum, tissue distribution, and associated cancers for each SNP, which allowed us to compare these findings with the data from the All of Us cohort.

The mutations were extracted using COSMIC’s online tools, which offer a range of filtering options, including mutation type and tissue of origin. For each SNP identified in COSMIC, we recorded the number of samples carrying the mutation and linked this information to disease associations as documented in the literature. For consistency reasons with All of Us, we used only missense mutations that were found in at least 20 individuals.

### Comparison between All of Us and COSMIC

After retrieving the relevant SNP data from both databases, we manually conducted a comparative analysis to identify the overlap between mutations found in both resources and mutations unique to each dataset. This work had to be done manually since, currently, no data transfer between COSMIC and All of Us is allowed. This comparison enabled us to assess the clinical relevance of mutations across both general and cancer-focused populations. For mutations present in both datasets, we further reviewed existing literature to determine their disease associations and clinical implications. We investigated the relevance of mutations found only in COSMIC to specific cancer types. Similarly, mutations exclusive to All of Us were analyzed in the context of population-specific genetic variation and disease prevalence.

## Electronic supplementary material

Below is the link to the electronic supplementary material.

Supplementary Material 1

Supplementary Material 2

## Data Availability

This study used data from the All of Us Research Program’s controlled Tier Dataset v.7, available to authorized users on the Researcher Workbench (https://databrowser.researchallofus.org/). Data from COSMIC v100 is available at (https://cancer.sanger.ac.uk/cosmic).
