# Supplementary material for: Spotlight on amino acid changing mutations in the JAK-STAT pathway: from disease-specific mutation to general mutation databases
Source: Sci Rep. 2025 Feb 20;15:6202. doi: 10.1038/s41598-025-90788-5 (PMC11842829; doi:10.1038/s41598-025-90788-5)
Supplement: Supplementary file 1 — Supplementary Material 1 [file 41598_2025_90788_MOESM1_ESM.docx]

**Domain genomic positions for: *Spotlight on amino acid changing mutations in the JAK-STAT pathway from disease-specific mutation to general mutation databases***

Contents

[JAK1 2](#_Toc162340612)

[JAK2 3](#_Toc162340613)

[JAK3 4](#_Toc162340614)

[TYK2 5](#_Toc162340615)

[STAT1 6](#_Toc162340616)

[STAT2 7](#_Toc162340617)

[STAT3 8](#_Toc162340618)

[STAT4 9](#_Toc162340619)

[STAT5A 10](#_Toc162340620)

[STAT5B 11](#_Toc162340621)

[STAT6 12](#_Toc162340622)

**!!ALL COORDINATES ARE HG38 AND GENCODE V44!!**

# JAK1


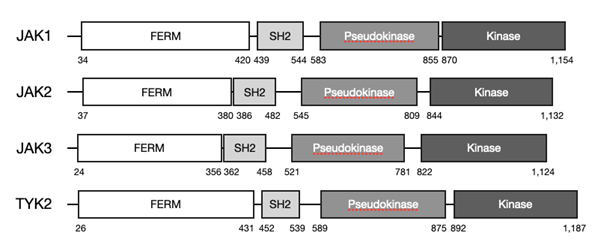


**Strandness: (-)**

| **Domain** | **Exon** | **Chr** | **Start** | **End** | **chr:start-end** | **Note** |
| --- | --- | --- | --- | --- | --- | --- |
| Starter_link | 2 | 1 | 64886259 | 64886264 | chr1:64886259-64886264 | Part of Exon 3 is still untranslated region |
| Starter_link | 3 | 1 | 64883383 | 64883475 | chr1:64883383-64883475 |  |
| FERM | 3 | 1 | 64883277 | 64883382 | chr1:64883277-64883382 | R69 - 1 base in Exon 3 |
| FERM | 4 | 1 | 64879025 | 64879148 | chr1:64879025-64879148 | R69 - 2 bases in Exon 4; R110 - 2 bases in Exon 4 |
| FERM | 5 | 1 | 64873370 | 64873523 | chr1:64873370-64873523 | R110 - 1 base in Exon 5 |
| FERM | 6 | 1 | 64869311 | 64869474 | chr1:64869311-64869474 | S216 - 2 bases in Exon 6 |
| FERM | 7 | 1 | 64866866 | 64867208 | chr1:64866866-64867208 | S216 - 1 base in Exon 7 |
| FERM | 8 | 1 | 64864787 | 64864972 | chr1:64864787-64864972 |  |
| FERM | 9 | 1 | 64860179 | 64860262 | chr1:64860179-64860262 |  |
| FERM_linker | 9 | 1 | 64860125 | 64860178 | chr1:64860125-64860178 |  |
| SH2 | 9 | 1 | 64860105 | 64860124 | chr1:64860105-64860124 | C445 - 2 bases in Exon 9 |
| SH2 | 10 | 1 | 64857656 | 64857779 | chr1:64857656-64857779 | C445 - 1 base in Exon 10 |
| SH2 | 11 | 1 | 64855525 | 64855696 | chr1:64855525-64855696 |  |
| SH2_linker | 11 | 1 | 64855509 | 64855524 | chr1:64855509-64855524 | E550 - 1 base in Exon 11 |
| SH2_linker | 12 | 1 | 64850810 | 64850910 | chr1:64850810-64850910 | E550 - 2 bases in Exon 12 |
| Pseudokinase | 12 | 1 | 64850804 | 64850809 | chr1:64850804-64850809 |  |
| Pseudokinase | 13 | 1 | 64847532 | 64847675 | chr1:64847532-64847675 |  |
| Pseudokinase | 14 | 1 | 64846649 | 64846736 | chr1:64846649-64846736 | N663 - 1 base in Exon 14 |
| Pseudokinase | 15 | 1 | 64845513 | 64845640 | chr1:64845513-64845640 | N663 - 2 bases in Exon 15 |
| Pseudokinase | 16 | 1 | 64844754 | 64844889 | chr1:64844754-64844889 | E751 - 1 base in Exon 16 |
| Pseudokinase | 17 | 1 | 64844064 | 64844215 | chr1:64844064-64844215 | E751 - 2 base in Exon 17 |
| Pseudokinase | 18 | 1 | 64841451 | 64841601 | chr1:64841451-64841601 | N852 - 1 base in Exon 18 |
| Pseudokinase | 19 | 1 | 64841329 | 64841339 | chr1:64841329-64841339 | N852 - 2 bases in Exon 19 |
| Pseudokinase_linker | 19 | 1 | 64841284 | 64841328 | chr1:64841284-64841328 |  |
| Kinase | 19 | 1 | 64841245 | 64841283 | chr1:64841245-64841283 |  |
| Kinase | 20 | 1 | 64839603 | 64839795 | chr1:64839603-64839795 | G948 - 1 base in Exon 20 |
| Kinase | 21 | 1 | 64838465 | 64838589 | chr1:64838465-64838589 | G948 - 2 bases in Exon 21 |
| Kinase | 22 | 1 | 64837932 | 64838104 | chr1:64837932-64838104 | W1047 - 2 bases in Exon 22 |
| Kinase | 23 | 1 | 64836098 | 64836215 | chr1:64836098-64836215 | W1047 - 1 base in Exon 23 |
| Kinase | 24 | 1 | 64835396 | 64835506 | chr1:64835396-64835506 |  |
| Kinase | 25 | 1 | 64834562 | 64834657 | chr1:64834562-64834657 |  |

| **Domain** | **Coordinates** |
| --- | --- |
| Starter_link | chr1:64883383-64886264 |
| FERM | chr1:64860179-64883382 |
| FERM_linker | chr1:64860125-64860178 |
| SH2 | chr1:64855525-64860124 |
| SH2_linker | chr1:64850810-64855524 |
| Pseudokinase | chr1:64841329-64850809 |
| Pseudokinase_linker | chr1:64841284-64841328 |
| Kinase | chr1:64834562-64841283 |

# JAK2


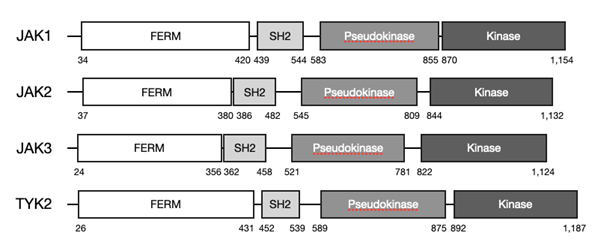


**Strandness: (+)**

| **Domain** | **Exon** | **Chr** | **Start** | **End** | **chr:start-end** | **Note** |
| --- | --- | --- | --- | --- | --- | --- |
| Starter_link | 3 | 9 | 5021988 | 5022095 | chr9:5021988-5022095 | Part of Exon 3 is still untranslated region |
| FERM | 3 | 9 | 5022096 | 5022213 | chr9:5022096-5022213 | G76 - 1 base in Exon 3 |
| FERM | 4 | 9 | 5029783 | 5029906 | chr9:5029783-5029906 | G76 - 2 bases in Exon 4; R116 - 2 bases in Exon 4 |
| FERM | 5 | 9 | 5044403 | 5044520 | chr9:5044403-5044520 | R116 - 1 base in Exon 5 |
| FERM | 6 | 9 | 5050685 | 5050831 | chr9:5050685-5050831 | S205 - 2 bases in Exon 6 |
| FERM | 7 | 9 | 5054563 | 5054884 | chr9:5054563-5054884 | S205 - 1 base in Exon 7 |
| FERM | 8 | 9 | 5055669 | 5055788 | chr9:5055669-5055788 |  |
| FERM | 9 | 9 | 5064883 | 5064966 | chr9:5064883-5064966 |  |
| FERM_linker | 9 | 9 | 5064967 | 5064981 | chr9:5064967-5064981 |  |
| SH2 | 9 | 9 | 5064982 | 5065040 | chr9:5064982-5065040 | S405 - 2 bases in Exon 9 |
| SH2 | 10 | 9 | 5066678 | 5066789 | chr9:5066678-5066789 | S405 - 1 base in Exon 10 |
| SH2 | 11 | 9 | 5069022 | 5069141 | chr9:5069022-5069141 |  |
| SH2_linker | 11 | 9 | 5069142 | 5069208 | chr9:5069142-5069208 | D505 - 1 base in Exon 11 |
| SH2_linker | 12 | 9 | 5069925 | 5070043 | chr9:5069925-5070043 | D505 - 2 bases in Exon 12 |
| Pseudokinase | 12 | 9 | 5070044 | 5070052 | chr9:5070044-5070052 |  |
| Pseudokinase | 13 | 9 | 5072492 | 5072626 | chr9:5072492-5072626 |  |
| Pseudokinase | 14 | 9 | 5073698 | 5073785 | chr9:5073698-5073785 | N622 - 1 base in Exon 14 |
| Pseudokinase | 15 | 9 | 5077453 | 5077580 | chr9:5077453-5077580 | N622 - 2 bases in Exon 15 |
| Pseudokinase | 16 | 9 | 5078306 | 5078444 | chr9:5078306-5078444 | I711 - 1 base in Exon 16 |
| Pseudokinase | 17 | 9 | 5080229 | 5080380 | chr9:5080229-5080380 | I711 - 2 bases in Exon 17 |
| Pseudokinase | 18 | 9 | 5080533 | 5080676 | chr9:5080533-5080676 |  |
| Pseudokinase_linker | 18 | 9 | 5080677 | 5080683 | chr9:5080677-5080683 | D812 - 1 base in Exon 18 |
| Pseudokinase_linker | 19 | 9 | 5081725 | 5081819 | chr9:5081725-5081819 | D812 - 2 bases in Exon 19 |
| Kinase | 19 | 9 | 5081820 | 5081861 | chr9:5081820-5081861 |  |
| Kinase | 20 | 9 | 5089674 | 5089863 | chr9:5089674-5089863 | G921 - 1 base in Exon 20 |
| Kinase | 21 | 9 | 5090446 | 5090570 | chr9:5090446-5090570 | G921 - 2 bases in Exon 21 |
| Kinase | 22 | 9 | 5090739 | 5090911 | chr9:5090739-5090911 | W1020 - 2 bases in Exon 22 |
| Kinase | 23 | 9 | 5123004 | 5123121 | chr9:5123004-5123121 | W1020 - 1 base in Exon 23 |
| Kinase | 24 | 9 | 5126333 | 5126446 | chr9:5126333-5126446 |  |
| Kinase | 25 | 9 | 5126684 | 5126791 | chr9:5126684-5126791 |  |

| **Domain** | **Coordinates** |
| --- | --- |
| Starter_link | chr9:5021988-5022095 |
| FERM | chr9:5022096-5064966 |
| FERM_linker | chr9:5064967-5064981 |
| SH2 | chr9:5064982-5069141 |
| SH2_linker | chr9:5069142-5070043 |
| Pseudokinase | chr9:5070044-5080676 |
| Pseudokinase_linker | chr9:5080677-5081819 |
| Kinase | chr9:5081820-5126791 |

# JAK3


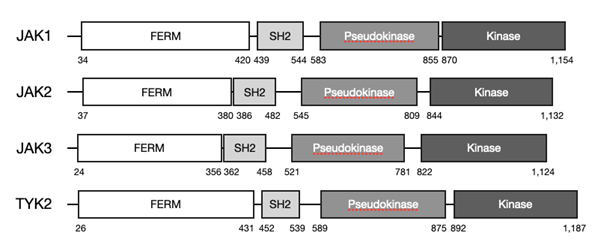


**Strandness: (-)**

| **Domain** | **Exon** | **Chr** | **Start** | **End** | **chr:start-end** | **Note** |
| --- | --- | --- | --- | --- | --- | --- |
| Starter_link | 2 | 19 | 17844349 | 17844417 | chr19:17844349-17844417 | Part of Exon 3 is still untranslated region |
| FERM | 2 | 19 | 17844234 | 17844348 | chr19:17844234-17844348 | G62 - 1 base on Exon 2 |
| FERM | 3 | 19 | 17843777 | 17843900 | chr19:17843777-17843900 | G62 - 2 bases on Exon 3; R103 - 2 bases on Exon 3 |
| FERM | 4 | 19 | 17843380 | 17843491 | chr19:17843380-17843491 | R103 - 1 base on Exon 4 |
| FERM | 5 | 19 | 17843027 | 17843172 | chr19:17843027-17843172 | S189 - 2 bases in Exon 5 |
| FERM | 6 | 19 | 17842316 | 17842610 | chr19:17842316-17842610 | S189 - 1 base in Exon 6 |
| FERM | 7 | 19 | 17841640 | 17841762 | chr19:17841640-17841762 |  |
| FERM | 8 | 19 | 17841463 | 17841546 | chr19:17841463-17841546 |  |
| FERM_linker | 8 | 19 | 17841448 | 17841462 | chr19:17841448-17841462 |  |
| SH2 | 8 | 19 | 17841389 | 17841447 | chr19:17841389-17841447 | T381 - 2 bases in Exon 8 |
| SH2 | 9 | 19 | 17840230 | 17840341 | chr19:17840230-17840341 |  |
| SH2 | 10 | 19 | 17839544 | 17839663 | chr19:17839544-17839663 |  |
| SH2_linker | 10 | 19 | 17839477 | 17839543 | chr19:17839477-17839543 | E481 - 1 base in Exon 10 |
| SH2_linker | 11 | 19 | 17838272 | 17838390 | chr19:17838272-17838390 | E481 - 2 bases in Exon 11 |
| Pseudokinase | 11 | 19 | 17838263 | 17838271 | chr19:17838263-17838271 |  |
| Pseudokinase | 12 | 19 | 17837932 | 17838063 | chr19:17837932-17838063 |  |
| Pseudokinase | 13 | 19 | 17837129 | 17837213 | chr19:17837129-17837213 | S596 - 1 base in Exon 13 |
| Pseudokinase | 14 | 19 | 17835924 | 17836051 | chr19:17835924-17836051 | S596 - 2 bases in Exon 14 |
| Pseudokinase | 15 | 19 | 17835083 | 17835215 | chr19:17835083-17835215 | M683 - 1 base in Exon 15 |
| Pseudokinase | 16 | 19 | 17834852 | 17835003 | chr19:17834852-17835003 | M683 - 2 bases in Exon 16 |
| Pseudokinase | 17 | 19 | 17834578 | 17834721 | chr19:17834578-17834721 |  |
| Pseudokinase_linker | 17 | 19 | 17834571 | 17834577 | chr19:17834571-17834577 | D784 - 1 base in Exon 17 |
| Pseudokinase_linker | 18 | 19 | 17832817 | 17832929 | chr19:17832817-17832929 |  |
| Kinase | 18 | 19 | 17832790 | 17832816 | chr19:17832790-17832816 |  |
| Kinase | 19 | 19 | 17832519 | 17832708 | chr19:17832519-17832708 | G894 - 1 base in Exon 19 |
| Kinase | 20 | 19 | 17831674 | 17831798 | chr19:17831674-17831798 | G894 - 2 bases in Exon 20 |
| Kinase | 21 | 19 | 17831228 | 17831400 | chr19:17831228-17831400 | W993 - 2 bases in Exon 21 |
| Kinase | 22 | 19 | 17830503 | 17830620 | chr19:17830503-17830620 | W993 - a base in Exon 22 |
| Kinase | 23 | 19 | 17830108 | 17830218 | chr19:17830108-17830218 |  |
| Kinase | 24 | 19 | 17826743 | 17826910 | chr19:17826743-17826910 |  |

| **Domain** | **Coordinates** |
| --- | --- |
| Starter_link | chr19:17844349-17844417 |
| FERM | chr19:17841463-17844348 |
| FERM_linker | chr19:17841448-17841462 |
| SH2 | chr19:17839544-17841447 |
| SH2_linker | chr19:17838272-17839543 |
| Pseudokinase | chr19:17834578-17838271 |
| Pseudokinase_linker | chr19:17832817-17834577 |
| Kinase | chr19:17826743-17832816 |

# TYK2


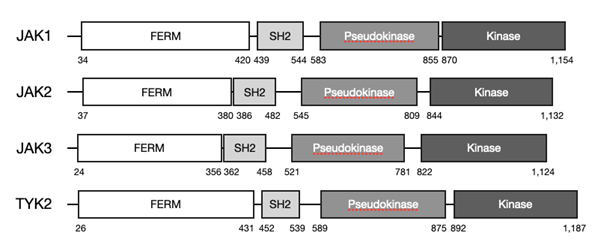


**Strandness: (-)**

| **Domain** | **Exon** | **Chr** | **Start** | **End** | **chr:start-end** | **Note** |
| --- | --- | --- | --- | --- | --- | --- |
| Starter_link | 3 | 19 | 10378332 | 10378406 | chr19:10378332-10378406 | Part of Exon 3 is still untranslated region |
| FERM | 3 | 19 | 10378214 | 10378331 | chr19:10378214-10378331 | G65 - 1 base in Exon 3 |
| FERM | 4 | 19 | 10368295 | 10368418 | chr19:10368295-10368418 | G65 - 2 bases in Exon 4; R106 - 2 bases in Exon 4 |
| FERM | 5 | 19 | 10368055 | 10368202 | chr19:10368055-10368202 | R106 - 1 base in Exon 5 |
| FERM | 6 | 19 | 10366417 | 10366580 | chr19:10366417-10366580 | S210 - 2 bases in Exon 6 |
| FERM | 7 | 19 | 10365517 | 10365898 | chr19:10365517-10365898 | S210 - 1 base in Exon 7 |
| FERM | 8 | 19 | 10364851 | 10365048 | chr19:10364851-10365048 |  |
| FERM | 9 | 19 | 10364688 | 10364771 | chr19:10364688-10364771 |  |
| FERM_linker | 9 | 19 | 10364628 | 10364687 | chr19:10364628-10364687 |  |
| SH2 | 9 | 19 | 10364614 | 10364627 | chr19:10364614-10364627 | L456 - 2 bases in Exon 9 |
| SH2 | 10 | 19 | 10362549 | 10362657 | chr19:10362549-10362657 | L456 - 1 base in Exon 10 |
| SH2 | 11 | 19 | 10362316 | 10362456 | chr19:10362316-10362456 |  |
| SH2_linker | 11 | 19 | 10362264 | 10362315 | chr19:10362264-10362315 | E557 - 1 base in Exon 11 |
| SH2_linker | 12 | 19 | 10362089 | 10362181 | chr19:10362089-10362181 | E557 - 2 bases in Exon 12 |
| Pseudokinase | 12 | 19 | 10362078 | 10362088 | chr19:10362078-10362088 |  |
| Pseudokinase | 13 | 19 | 10361770 | 10361955 | chr19:10361770-10361955 |  |
| Pseudokinase | 14 | 19 | 10361511 | 10361598 | chr19:10361511-10361598 | N683 - 1 base in Exon 14 |
| Pseudokinase | 15 | 19 | 10359175 | 10359302 | chr19:10359175-10359302 | N683 - 2 bases in Exon 15 |
| Pseudokinase | 16 | 19 | 10358003 | 10358138 | chr19:10358003-10358138 | E771 - 1 base in Exon 16 |
| Pseudokinase | 17 | 19 | 10357764 | 10357918 | chr19:10357764-10357918 | E771 - 2 bases in Exon 17 |
| Pseudokinase | 18 | 19 | 10356568 | 10356718 | chr19: 10356568-10356718 | N873 - 1 base in Exon 18 |
| Pseudokinase | 19 | 19 | 10354602 | 10354609 | chr19:10354602-10354609 | N873 - 2 bases in Exon 19 |
| Pseudokinase_linker | 19 | 19 | 10354554 | 10354601 | chr19:10354554-10354601 |  |
| Kinase | 19 | 19 | 10354512 | 10354553 | chr19:10354512-10354553 |  |
| Kinase | 20 | 19 | 10354042 | 10354234 | chr19:10354042-10354234 | G970 - 1 base in Exon 20 |
| Kinase | 21 | 19 | 10353528 | 10353646 | chr19:10353528-10353646 | G970 - 2 bases in Exon 21 |
| Kinase | 22 | 19 | 10352926 | 10353098 | chr19:10352926-10353098 | W1067 - 2 bases in Exon 22 |
| Kinase | 23 | 19 | 10352434 | 10352551 | chr19:10352434-10352551 | W1067 - 1 base in Exon 23 |
| Kinase | 24 | 19 | 10351052 | 10351162 | chr19:10351052-10351162 |  |
| Kinase | 25 | 19 | 10350834 | 10350968 | chr19:10350834-10350968 |  |

| **Domain** | **Coordinates** |
| --- | --- |
| Starter_link | chr19:10378332-10378406 |
| FERM | chr19:10364688-10378331 |
| FERM_linker | chr19:10364628-10364687 |
| SH2 | chr19:10362316-10364627 |
| SH2_linker | chr19:10362089-10362315 |
| Pseudokinase | chr19:10354602-10362088 |
| Pseudokinase_linker | chr19:10354554-10354601 |
| Kinase | chr19:10350834-10354553 |

# STAT1


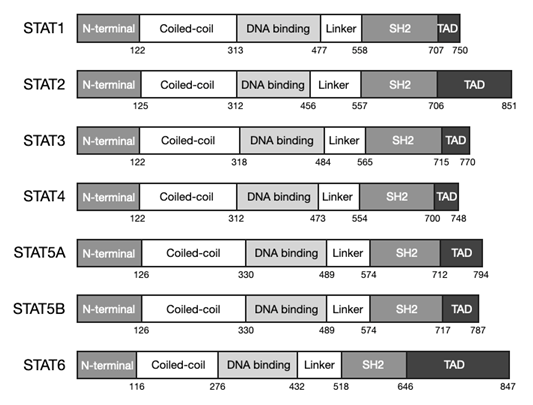


**Strandness: (-)**

| **Domain** | **Exon** | **Chr** | **Start** | **End** | **chr:start-end** | **Note** |
| --- | --- | --- | --- | --- | --- | --- |
| 5’ UTR | 1,2,3 | 2 | 191010004 | 191013678 | chr2:191010004-191013678 |  |
| NTerminal | 3 | 2 | 191009876 | 191010003 | chr2:191009876-191010003 | UTR on Exon 3; W43 - 2 bases on Exon 3 |
| NTerminal | 4 | 2 | 191008963 | 191009107 | chr2: 191008963-191009107 | W43 - 1 base in Exon 4 |
| NTerminal | 5 | 2 | 191007569 | 191007661 | chr2:191007569-191007661 |  |
| CoiledCoil | 5 | 2 | 191007563 | 191007568 | chr2:191007563-191007568 |  |
| CoiledCoil | 6 | 2 | 191001074 | 191001163 | chr2:191001074-191001163 |  |
| CoiledCoil | 7 | 2 | 190999626 | 190999704 | chr2:190999626-190999704 | E181 - 1 base in Exon 7 |
| CoiledCoil | 8 | 2 | 190998217 | 190998308 | chr2:190998217-190998308 | E181 - 2 bases in Exon 8 |
| CoiledCoil | 9 | 2 | 190997856 | 190998007 | chr2:190997856-190998007 | W262 - 1 base in Exon 9 |
| CoiledCoil | 10 | 2 | 190995066 | 190995219 | chr2:190995066-190995219 | W262 - 2 bases in Exon 9 |
| DNAbinding | 10 | 2 | 190995061 | 190995065 | chr2:190995061-190995065 | S215 - 2 bases in Exon 10 |
| DNAbinding | 11 | 2 | 190991228 | 190991320 | chr2: 190991228-190991320 | S215 - 1 base in Exon 11; R346 - 2 bases in Exon 11 |
| DNAbinding | 12 | 2 | 190989615 | 190989674 | chr2:190989615-190989674 | R346 - 1 base in Exon 12; K366 - 2 bases in Exon 12 |
| DNAbinding | 13 | 2 | 190987039 | 190987068 | chr2:190987039-190987068 | K366 - 1 base in Exon 13; G376 - 2 bases in Exon 13 |
| DNAbinding | 14 | 2 | 190986854 | 190986947 | chr2:190986854-190986947 | G376 - 1 base in Exon 14 |
| DNAbinding | 15 | 2 | 190985619 | 190985660 | chr2:190985619-190985660 |  |
| DNAbinding | 16 | 2 | 190984310 | 190984393 | chr2:190984310-190984393 |  |
| DNAbinding | 17 | 2 | 190983657 | 190983740 | chr2:190983657-190983740 |  |
| Linker | 17 | 2 | 190983642 | 190983656 | chr2:190983642-190983656 |  |
| Linker | 18 | 2 | 190982383 | 190982518 | chr2:190982383-190982518 | G528 - 1 base in Exon 18 |
| Linker | 19 | 2 | 190980620 | 190980669 | chr2:190980620-190980669 | G528 - 2 bases in Exon 19 |
| Linker | 20 | 2 | 190979825 | 190979866 | chr2:190979825-190979866 |  |
| SH2 | 20 | 2 | 190979772 | 190979824 | chr2:190979772-190979824 | G576 - 2 bases in Exon 20 |
| SH2 | 21 | 2 | 190978856 | 190979001 | chr2:190978856-190979001 | G576 - 1 base in Exon 21; E625 - 1 base in Exon 21 |
| SH2 | 22 | 2 | 190976840 | 190977025 | chr2:190976840-190977025 | E625 - 2 bases in Exon 22; A687 - 1 base in Exon 22 |
| SH2 | 23 | 2 | 190975826 | 190975887 | chr2:190975826-190975887 | A687 - 2 base in Exon 23 |
| TAD | 23 | 2 | 190975812 | 190975825 | chr2:190975812-190975825 | V712 - 2 bases in Exon 23 |
| TAD | 24 | 2 | 190974830 | 190974932 | chr2:190974830-190974932 | V712 - 1 base in Exon 24 |
| TAD | 25 | 2 | 190970703 | 190970717 | chr2:190970703-190970717 |  |

| **Domain** | **Coordinates** |
| --- | --- |
| NTerminal | chr2:191007569-191010003 |
| CoiledCoil | chr2:190995066-191007568 |
| DNAbinding | chr2:190983657-190995065 |
| Linker | chr2:190979825-190983656 |
| SH2 | chr2:190975826-190979824 |
| TAD | chr2:190970703-190975825 |

# STAT2


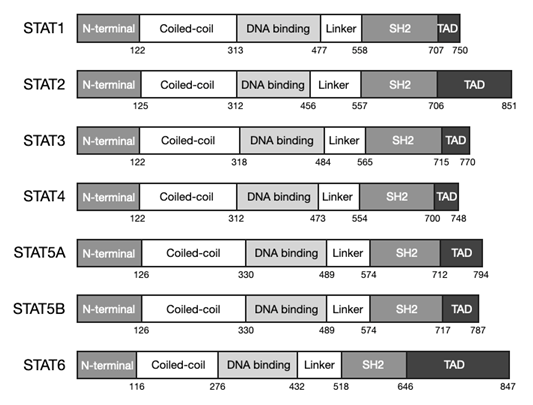


**Strandness: (-)**

| **Domain** | **Exon** | **Chr** | **Start** | **End** | **chr:start-end** | **Note** |
| --- | --- | --- | --- | --- | --- | --- |
| NTerminal | 2 | 12 | 56356441 | 56356571 | chr12:56356441-56356571 | UTR on Exon 2; W44 - 2 bases in Exon 2 |
| NTerminal | 3 | 12 | 56356132 | 56356285 | chr12:56356132-56356285 | W44 - 1 base in Exon 3 |
| NTerminal | 4 | 12 | 56355714 | 56355803 | chr12:56355714-56355803 |  |
| CoiledCoil | 4 | 12 | 56355708 | 56355713 | chr12:56355708-56355713 |  |
| CoiledCoil | 5 | 12 | 56355443 | 56355532 | chr12:56355443-56355532 |  |
| CoiledCoil | 6 | 12 | 56355276 | 56355351 | chr12:56355276-56355351 | G183 - 1 base in Exon 6 |
| CoiledCoil | 7 | 12 | 56354778 | 56354863 | chr12:56354778-56354863 | G183 - 2 bases in Exon 7 |
| CoiledCoil | 8 | 12 | 56354466 | 56354614 | chr12:56354466-56354614 | W261 - 2 bases in Exon 8 |
| CoiledCoil | 9 | 12 | 56351297 | 56351450 | chr12:56351297-56351450 | W261 - 1 base in Exon 9 |
| DNAbinding | 9 | 12 | 56351292 | 56351296 | chr12:56351292-56351296 | R314 - 2 bases in Exon 9 |
| DNAbinding | 10 | 12 | 56351098 | 56351190 | chr12:56351098-56351190 | R314 - 1 base in Exon 10; R345 - 2 bases in Exon 10 |
| DNAbinding | 11 | 12 | 56350829 | 56350888 | chr12:56350829-56350888 | R345 - 1 base in Exon 11; R365 - 2 bases in Exon 11 |
| DNAbinding | 12 | 12 | 56350412 | 56350432 | chr12:56350412-56350432 | R365 - 1 base in Exon 12; G372 - 2 bases in Exon 12 |
| DNAbinding | 13 | 12 | 56350097 | 56350190 | chr12:56350097-56350190 | G372 - 1 base in Exon 13 |
| DNAbinding | 14 | 12 | 56349589 | 56349636 | chr12:56349589-56349636 |  |
| DNAbinding | 15 | 12 | 56349426 | 56349509 | chr12:56349426-56349509 |  |
| DNAbinding | 16 | 12 | 56349235 | 56349261 | chr12:56349235-56349261 |  |
| Linker | 16 | 12 | 56349163 | 56349234 | chr12:56349163-56349234 |  |
| Linker | 17 | 12 | 56348924 | 56349059 | chr12:56348924-56349059 | G526 - 1 base in Exon 17 |
| Linker | 18 | 12 | 56348752 | 56348804 | chr12:56348752-56348804 | G526 - 2 bases in Exon 18 |
| Linker | 19 | 12 | 56348582 | 56348623 | chr12:56348582-56348623 |  |
| SH2 | 19 | 12 | 56348529 | 56348581 | chr12:56348529-56348581 | G575 - 2 bases in Exon 19 |
| SH2 | 20 | 12 | 56346819 | 56346955 | chr12:56346819-56346955 | G575 - 1 base in Exon 20; D621 - 1 base in Exon 20 |
| SH2 | 21 | 12 | 56346442 | 56346624 | chr12:56346442-56346624 | D621 - 2 bases in Exon 21; V682 - 1 base in Exon 21 |
| SH2 | 22 | 12 | 56346146 | 56346203 | chr12:56346146-56346203 | V682 - 2 bases in Exon 22; R701 - 2 bases in Exon 22 |
| SH2 | 23 | 12 | 56344120 | 56344135 | chr12:56344120-56344135 | R701 - 1 base in Exon 23 |
| TAD | 23 | 12 | 56343825 | 56344119 | chr12:56343825-56344119 | I805 - 1 base in Exon 23 |
| TAD | 24 | 12 | 56343389 | 56343531 | chr12:56343389-56343531 | I805 - 2 bases in Exon 24 |

| **Domain** | **Coordinates** |
| --- | --- |
| NTerminal | chr12:56355714-56356571 |
| CoiledCoil | chr12:56351297-56355713 |
| DNAbinding | chr12:56349235-56351296 |
| Linker | chr12:56348582-56349234 |
| SH2 | chr12:56344120-56348581 |
| TAD | chr12:56343389-56344119 |

# STAT3


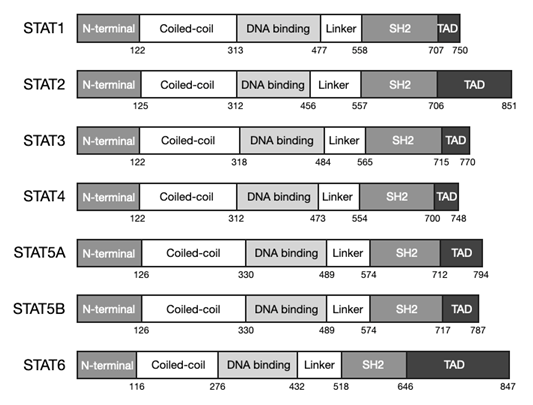


**Strandness: (-)**

| **Domain** | **Exon** | **Chr** | **Start** | **End** | **chr:start-end** | **Note** |
| --- | --- | --- | --- | --- | --- | --- |
| NTerminal | 2 | 17 | 42348389 | 42348516 | chr17:42348389-42348516 | UTR on Exon 2; W43 - 2 bases on Exon 2 |
| NTerminal | 3 | 17 | 42346569 | 42346713 | chr17:42346569-42346713 | W43 - 1 base on Exon 3 |
| NTerminal | 4 | 17 | 42345568 | 42345657 | chr17:42345568-42345657 |  |
| CoiledCoil | 4 | 17 | 42345559 | 42345567 | chr17:42345559-42345567 |  |
| CoiledCoil | 5 | 17 | 42339314 | 42339409 | chr17:42339314-42339409 |  |
| CoiledCoil | 6 | 17 | 42338731 | 42338812 | chr17:42338731-42338812 | D184 - 1 base in Exon 6 |
| CoiledCoil | 7 | 17 | 42337763 | 42337857 | chr17:42337763-42337857 | D184 - 2 bases in Exon 7 |
| CoiledCoil | 8 | 17 | 42337435 | 42337586 | chr17:42337435-42337586 | W266 - 2 bases in Exon 8 |
| CoiledCoil | 9 | 17 | 42333893 | 42334049 | chr17:42333892-42334049 | W266 - 1 base in Exon 9 |
| DNAbinding | 9 | 17 | 42333891 | 42333892 | chr17:42333891-42333892 | S319 - 2 bases in Exon 9 |
| DNAbinding | 10 | 17 | 42333673 | 42333765 | chr17:42333673-42333765 | S319 - 1 base in Exon 10, R350 - 2 bases in Exon 10 |
| DNAbinding | 11 | 17 | 42331472 | 42331531 | chr17:42331472-42331531 | R350 - 1 base in Exon 11; K370 - 2 bases in Exon 11 |
| DNAbinding | 12 | 17 | 42329747 | 42329776 | chr17:42329747-42329776 | K370 - 1 base in Exon 12; G380 - 2 bases in Exon 12 |
| DNAbinding | 13 | 17 | 42329554 | 42329647 | chr17:42329554-42329647 | G380 - 1 base in Exon 13 |
| DNAbinding | 14 | 17 | 42329410 | 42329457 | chr17:42329410-42329457 |  |
| DNAbinding | 15 | 17 | 42326116 | 42326199 | chr17:42326116-42326199 |  |
| DNAbinding | 16 | 17 | 42324975 | 42325061 | chr17:42324975-42325061 |  |
| Linker | 16 | 17 | 42324963 | 42324974 | chr17:42324963-42324974 |  |
| Linker | 17 | 17 | 42324711 | 42324846 | chr17:42324711-42324846 | G534 - 1 base in Exon 17 |
| Linker | 18 | 17 | 42323573 | 42323625 | chr17:42323573-42323625 | G534 - 2 bases in Exon 18 |
| Linker | 19 | 17 | 42323313 | 42323354 | chr17:42323313-42323354 |  |
| SH2 | 19 | 17 | 42323260 | 42323312 | chr17:42323260-42323312 | G583 - 2 bases in Exon 19 |
| SH2 | 20 | 17 | 42323004 | 42323143 | chr17:42323004-42323143 | G583 - 1 base in Exon 20; G630 - 1 base in Exon 20 |
| SH2 | 21 | 17 | 42322282 | 42322494 | chr17:42322282-42322494 | G630 - 2 bases in Exon 21; S701 - 1 base in Exon 21 |
| SH2 | 22 | 17 | 42317182 | 42317224 | chr17:42317182-42317224 | S701 - 2 bases in Exon 22; P715 - 2 bases in Exon 22 |
| SH2 | 23 | 17 | 42316901 | 42316901 | chr17:42316901-42316901 | P715 - 1 base in Exon 23 |
| TAD | 23 | 17 | 42316789 | 42316900 | chr17:42316789-42316900 | E753 - 1 base in Exon 23 |
| TAD | 24 | 17 | 42315745 | 42315800 | chr17:42315745-42315800 | E753 - 2 bases in Exon 24 |

| **Domain** | **Coordinates** |
| --- | --- |
| NTerminal | chr17:42345568-42348516 |
| CoiledCoil | chr17:42333892-42345567 |
| DNAbinding | chr17:42324975-42333892 |
| Linker | chr17:42323313-42324974 |
| SH2 | chr17:42316901-42323312 |
| TAD | chr17:42315745-42316900 |

# STAT4


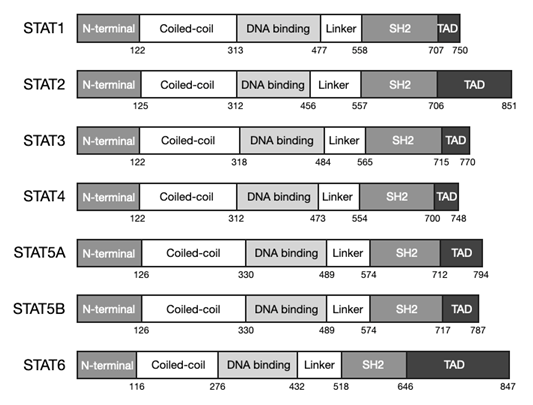


**Strandness: (-)**

| **Domain** | **Exon** | **Chr** | **Start** | **End** | **chr:start-end** | **Note** |
| --- | --- | --- | --- | --- | --- | --- |
| NTerminal | 2 | 2 | 191148076 | 191148203 | chr2:191148076-191148203 | UTR on Exon 2; W43 - 2 bases in Exon 2 |
| NTerminal | 3 | 2 | 191146613 | 191146757 | chr2: 191146613-191146757 | W43 - 1 base in Exon 3 |
| NTerminal | 4 | 2 | 191076233 | 191076325 | chr2:191076233-191076325 |  |
| CoiledCoil | 4 | 2 | 191076227 | 191076232 | chr2:191076227-191076232 |  |
| CoiledCoil | 5 | 2 | 191073098 | 191073190 | chr2:191073098-191073190 |  |
| CoiledCoil | 6 | 2 | 191069693 | 191069771 | chr2:191069693-191069771 | D182 - 1 base in Exon 6 |
| CoiledCoil | 7 | 2 | 191066430 | 191066515 | chr2:191066430-191066515 | D182 - 2 bases in Exon 7 |
| CoiledCoil | 8 | 2 | 191064807 | 191064958 | chr2:191064807-191064958 | C261 - 2 bases in Exon 8 |
| CoiledCoil | 9 | 2 | 191062767 | 191062920 | chr2:191062767-191062920 | C261 - 1 base in Exon 9 |
| DNAbinding | 9 | 2 | 191062762 | 191062766 | chr2:191062762-191062766 | N314 - 2 bases in Exon 9 |
| DNAbinding | 10 | 2 | 191061729 | 191061821 | chr2:191061729-191061821 | N314 - 1 base in Exon 10; R345 - 2 bases in Exon 10 |
| DNAbinding | 11 | 2 | 191058710 | 191058769 | chr2:191058710-191058769 | R345 - 1 base in Exon 11; K365 - 2 bases in Exon 11 |
| DNAbinding | 12 | 2 | 191058202 | 191058219 | chr2:191058202-191058219 | K365 - 1 base in Exon 12; S371 - 2 bases in Exon 12 |
| DNAbinding | 13 | 2 | 191058018 | 191058111 | chr2:191058018-191058111 | S371 - 1 base in Exon 13 |
| DNAbinding | 14 | 2 | 191054490 | 191054534 | chr2:191054490-191054534 |  |
| DNAbinding | 15 | 2 | 191041065 | 191041148 | chr2:191041065-191041148 |  |
| DNAbinding | 16 | 2 | 191039214 | 191039297 | chr2:191039214-191039297 |  |
| Linker | 16 | 2 | 191039199 | 191039213 | chr2:191039199-191039213 |  |
| Linker | 17 | 2 | 191036164 | 191036299 | chr2:191036164-191036299 | V524 - 1 base in Exon 17 |
| Linker | 18 | 2 | 191034548 | 191034597 | chr2:191034548-191034597 | V524 - 2 bases in Exon 18 |
| Linker | 19 | 2 | 191033964 | 191034005 | chr2:191033964-191034005 |  |
| SH2 | 19 | 2 | 191033911 | 191033963 | chr2:191033911-191033963 | G572 - 2 bases in Exon 19 |
| SH2 | 20 | 2 | 191033490 | 191033626 | chr2:191033490-191033626 | G572 - a base in Exon 20; G618 - 1 base in Exon 20 |
| SH2 | 21 | 2 | 191032958 | 191033149 | chr2:191032958-191033149 | G618 - 2 bases in Exon 21; V682 - 1 base in Exon 21 |
| SH2 | 22 | 2 | 191031461 | 191031516 | chr2:191031461-191031516 | V682 - 2 bases in Exon 22 |
| TAD | 22 | 2 | 191031450 | 191031460 | chr2:191031450-191031460 | I704 - 2 bases in Exon 22 |
| TAD | 23 | 2 | 191030972 | 191031080 | chr2:191030972-191031080 | I704 - 1 base in Exon 23 |
| TAD | 24 | 2 | 191029840 | 191029865 | chr2:191029840-191029865 |  |

| **Domain** | **Coordinates** |
| --- | --- |
| NTerminal | chr2:191076233-191148203 |
| CoiledCoil | chr2:191062767-191076232 |
| DNAbinding | chr2:191039214-191062766 |
| Linker | chr2:191033964-191039213 |
| SH2 | chr2:191031461-191033963 |
| TAD | chr2:191029840-191031460 |

# STAT5A


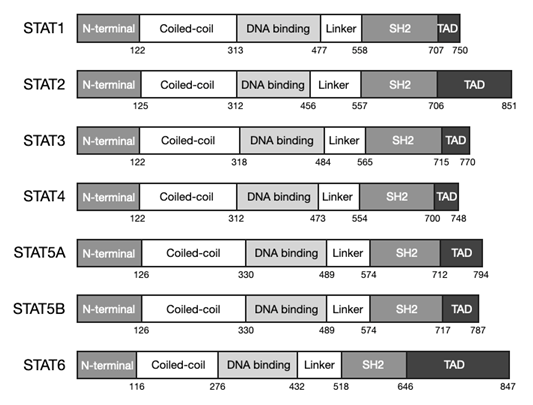


**Strandness: (+)**

| **Domain** | **Exon** | **Chr** | **Start** | **End** | **chr:start-end** | **Note** |
| --- | --- | --- | --- | --- | --- | --- |
| NTerminal | 2 | 17 | 42289412 | 42289539 | chr17:42289412-42289539 | UTR on Exon 2; W43 - 2 bases in Exon 2 |
| NTerminal | 3 | 17 | 42289866 | 42290022 | chr17:42289866-42290022 | W43 - 1 base in Exon 3 |
| NTerminal | 4 | 17 | 42291972 | 42292061 | chr17:42291972-42292061 |  |
| NTerminal | 5 | 17 | 42295619 | 42295621 | chr17:42295619-42295621 |  |
| CoiledCoil | 5 | 17 | 42295622 | 42295793 | chr17:42295622-42295793 | A184 - 1 base in Exon 5 |
| CoiledCoil | 6 | 17 | 42299751 | 42299881 | chr17:42299751-42299881 | A184 - 2 bases in Exon 6 |
| CoiledCoil | 7 | 17 | 42300130 | 42300281 | chr17:42300130-42300281 | W278 - 2 bases in Exon 7 |
| CoiledCoil | 8 | 17 | 42300715 | 42300870 | chr17:42300715-42300870 | W278 - 1 base in Exon 8; S330 - 2 bases on Exon 8 |
| CoiledCoil | 9 | 17 | 42301275 | 42301275 | chr17:42301275-42301275 | S330 - 1 base on Exon 9 |
| DNAbinding | 9 | 17 | 42301276 | 42301454 | chr17:42301276-42301454 | N390 - 2 bases in Exon 9 |
| DNAbinding | 10 | 17 | 42304342 | 42304429 | chr17:42304342-42304429 | N390 - 1 base in Exon 10 |
| DNAbinding | 11 | 17 | 42304530 | 42304652 | chr17:42304530-42304652 |  |
| DNAbinding | 12 | 17 | 42305610 | 42305696 | chr17:42305610-42305696 |  |
| Linker | 12 | 17 | 42305697 | 42305702 | chr17:42305697-42305702 |  |
| Linker | 13 | 17 | 42306241 | 42306447 | chr17:42306241-42306447 |  |
| Linker | 14 | 17 | 42307402 | 42307443 | chr17:42307402-42307443 |  |
| SH2 | 14 | 17 | 42307444 | 42307496 | chr17:42307444-42307496 | G592 - 2 bases in Exon 14 |
| SH2 | 15 | 17 | 42307593 | 42307725 | chr17:42307593-42307725 | G592 - 1 base in Exon 15; S636 - 1 base in Exon 15 |
| SH2 | 16 | 17 | 42308178 | 42308333 | chr17:42308178-42308333 | S636 - 2 bases in Exon 16; A688 - 1 base in Exon 16 |
| SH2 | 17 | 17 | 42309047 | 42309098 | chr17:42309047-42309098 | A688 - 2 bases in Exon 17; E705 - 2 bases in Exon 17 |
| SH2 | 18 | 17 | 42309377 | 42309398 | chr17:42309377-42309398 | E705 - 1 base in Exon 18 |
| TAD | 18 | 17 | 42309399 | 42309484 | chr17:42309399-42309484 | N741 - 2 bases in Exon 18 |
| TAD | 19 | 17 | 42310507 | 42310669 | chr17:42310507-42310669 | N741 -1 base in Exon 19 |

| **Domain** | **Coordinates** |
| --- | --- |
| NTerminal | chr17:42289412-42295621 |
| CoiledCoil | chr17:42295622-42301275 |
| DNAbinding | chr17:42301276-42305696 |
| Linker | chr17:42305697-42307443 |
| SH2 | chr17:42307444-42309398 |
| TAD | chr17:42309399-42310669 |

# STAT5B


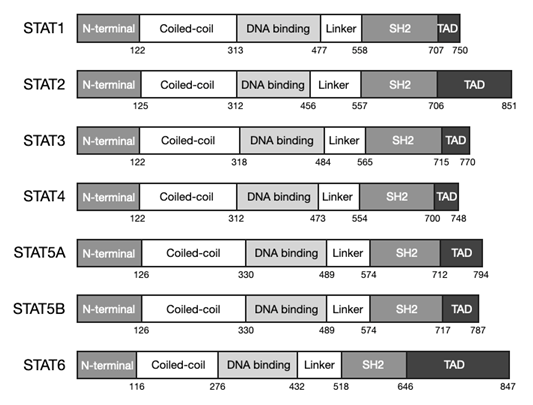


**Strandness: (-)**

| **Domain** | **Exon** | **Chr** | **Start** | **End** | **chr:start-end** | **Note** |
| --- | --- | --- | --- | --- | --- | --- |
| NTerminal | 2 | 17 | 42232000 | 42232127 | chr17:42232000-42232127 | UTR on Exon 2; W43 - 2 bases in Exon 2 |
| NTerminal | 3 | 17 | 42227529 | 42227685 | chr17:42227529-42227685 | W43 - 1 base in Exon 3 |
| NTerminal | 4 | 17 | 42224779 | 42224868 | chr17:42224779-42224868 |  |
| NTerminal | 5 | 17 | 42223554 | 42223556 | chr17:42223554-42223556 |  |
| CoiledCoil | 5 | 17 | 42223382 | 42223553 | chr17:42223382-42223553 | A184 - 1 base in Exon 5 |
| CoiledCoil | 6 | 17 | 42219712 | 42219842 | chr17:42219712-42219842 | A184 - 2 bases in Exon 6 |
| CoiledCoil | 7 | 17 | 42219312 | 42219463 | chr17:42219312-42219463 | W278 - 2 bases in Exon 7 |
| CoiledCoil | 8 | 17 | 42218723 | 42218878 | chr17:42218723-42218878 | W278 - 1 base in Exon 8; S330 - 2 bases in Exon 8 |
| CoiledCoil | 9 | 17 | 42218330 | 42218330 | chr17:42218330-42218330 | S330 - 1 base in Exon 9 |
| DNAbinding | 9 | 17 | 42218151 | 42218329 | chr17:42218151-42218329 | N390 - 2 bases in Exon 9 |
| DNAbinding | 10 | 17 | 42217377 | 42217464 | chr17:42217377-42217464 | N390 - 1 base in Exon 10 |
| DNAbinding | 11 | 17 | 42217160 | 42217282 | chr17:42217160-42217282 |  |
| DNAbinding | 12 | 17 | 42216020 | 42216106 | chr17:42216020-42216106 |  |
| Linker | 12 | 17 | 42216014 | 42216019 | chr17:42216014-42216019 |  |
| Linker | 13 | 17 | 42211984 | 42212190 | chr17:42211984-42212190 |  |
| Linker | 14 | 17 | 42210456 | 42210497 | chr17:42210456-42210497 |  |
| SH2 | 14 | 17 | 42210403 | 42210455 | chr17:42210403-42210455 | G592 - 2 bases in Exon 14 |
| SH2 | 15 | 17 | 42210171 | 42210301 | chr17:42210171-42210301 | G592 - 1 base in Exon 15; Q636 - 1 base in Exon 15 |
| SH2 | 16 | 17 | 42207558 | 42207728 | chr17:42207558-42207728 | Q636 - 2 bases in Exon 16; A693 - 1 base in Exon 16 |
| SH2 | 17 | 17 | 42202757 | 42202808 | chr17:42202757-42202808 | A693 - 2 bases in Exon 17; E710 - 2 bases in Exon 17 |
| SH2 | 18 | 17 | 42202426 | 42202447 | chr17:42202426-42202447 | E710 - 1 base in Exon 18 |
| TAD | 18 | 17 | 42202340 | 42202425 | chr17:42202340-42202425 | N746 - 2 bases in Exon 18 |
| TAD | 19 | 17 | 42201738 | 42201864 | chr17:42201738-42201864 | N746 - 1 base in Exon 19 |

| **Domain** | **Coordinates** |
| --- | --- |
| NTerminal | chr17:42223554-42232127 |
| CoiledCoil | chr17:42218330-42223553 |
| DNAbinding | chr17:42216020-42218329 |
| Linker | chr17:42210456-42216019 |
| SH2 | chr17:42202426-42210455 |
| TAD | chr17:42201738-42202425 |

# STAT6


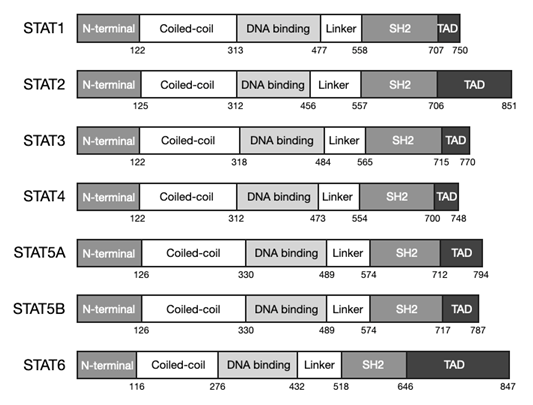


**Strandness: (-)**

| **Domain** | **Exon** | **Chr** | **Start** | **End** | **chr:start-end** | **Note** |
| --- | --- | --- | --- | --- | --- | --- |
| NTerminal | 2 | 12 | 57108163 | 57108278 | chr12:57108163-57108278 | UTR on Exon 2; W39 - 2 bases in Exon 2 |
| NTerminal | 3 | 12 | 57107605 | 57107743 | chr12:57107605-57107743 | W39 - 1 base in Exon 3 |
| NTerminal | 4 | 12 | 57107231 | 57107314 | chr12:57107231-57107314 |  |
| NTerminal | 5 | 12 | 57106823 | 57106831 | chr12:57106823-57106831 |  |
| CoiledCoil | 5 | 12 | 57106693 | 57106822 | chr12:57106693-57106822 | V160 - 1 base in Exon 5 |
| CoiledCoil | 6 | 12 | 57106528 | 57106580 | chr12:57106528-57106580 | V160 - 2 bases in Exon 6 |
| CoiledCoil | 7 | 12 | 57106191 | 57106339 | chr12:57106191-57106339 | R227 - 2 bases in Exon 7 |
| CoiledCoil | 8 | 12 | 57105468 | 57105599 | chr12:57105468-57105599 | R227 - 1 base in Exon 8; S271 - 2 bases in Exon 8 |
| CoiledCoil | 9 | 12 | 57105324 | 57105339 | chr12:57105324-57105339 | S271 - 1 base in Exon 9 |
| DNAbinding | 9 | 12 | 57105151 | 57105323 | chr12:57105151-57105323 | A334 - 2 bases in Exon 9 |
| DNAbinding | 10 | 12 | 57104726 | 57104813 | chr12:57104726-57104813 | A334 - 1 base in Exon 10 |
| DNAbinding | 11 | 12 | 57104464 | 57104586 | chr12:57104464-57104586 |  |
| DNAbinding | 12 | 12 | 57102838 | 57102921 | chr12:57102838-57102921 |  |
| Linker | 12 | 12 | 57102829 | 57102837 | chr12:57102829-57102837 |  |
| Linker | 13 | 12 | 57102290 | 57102496 | chr12:57102290-57102496 |  |
| Linker | 14 | 12 | 57100049 | 57100090 | chr12:57100049-57100090 |  |
| SH2 | 14 | 12 | 57099996 | 57100048 | chr12:57099996-57100048 | R536 - 2 bases in Exon 14 |
| SH2 | 15 | 12 | 57099767 | 57099903 | chr12:57099767-57099903 | R536 - 1 base in Exon 15; G582 - 2 bases in Exon 15 |
| SH2 | 16 | 12 | 57099294 | 57099440 | chr12:57099294-57099440 | G582 - 1 base in Exon 16; P631 - 1 base in Exon 16 |
| SH2 | 17 | 12 | 57099032 | 57099078 | chr12:57099032-57099078 | P631 - 2 bases in Exon 17 |
| TAD | 17 | 12 | 57099015 | 57099031 | chr12:57099015-57099031 | R652 - 2 bases in Exon 17 |
| TAD | 18 | 12 | 57098792 | 57098902 | chr12:57098792-57098902 | R652 - 1 base in Exon 18; V689 - 2 bases in Exon 18 |
| TAD | 19 | 12 | 57098505 | 57098597 | chr12:57098505-57098597 | V689 - 1 base in Exon 19; E720 - 2 bases in Exon 19 |
| TAD | 20 | 12 | 57097068 | 57097133 | chr12:57097068-57097133 | E720 - 1 base in Exon 20; Q742 - 2 bases in Exon 20 |
| TAD | 21 | 12 | 57096850 | 57096978 | chr12:57096850-57096978 | Q742 - 1 base in Exon 21; W785 - 2 bases in Exon 21 |
| TAD | 22 | 12 | 57096572 | 57096761 | chr12:57096572-57096761 | W785 - 1 base in Exon 22 |

| **Domain** | **Coordinates** |
| --- | --- |
| NTerminal | chr12:57106823-57108278 |
| CoiledCoil | chr12:57105324-57106822 |
| DNAbinding | chr12:57102838-57105323 |
| Linker | chr12:57100049-57102837 |
| SH2 | chr12:57099032-57100048 |
| TAD | chr12:57096572-57099031 |
