# Supplementary material for: Spotlight on amino acid changing mutations in the JAK-STAT pathway: from disease-specific mutation to general mutation databases
Source: Sci Rep. 2025 Feb 20;15:6202. doi: 10.1038/s41598-025-90788-5 (PMC11842829; doi:10.1038/s41598-025-90788-5)
Supplement: Supplementary file 2 — Supplementary Material 2 [file 41598_2025_90788_MOESM2_ESM.docx]

***Supplementary Materials for:* Spotlight on amino acid changing mutations in the JAK-STAT pathway from disease-specific mutation to general mutation databases**

Markus Hoffmann^1,*^ and Lothar Hennighausen^1^

^1^ National Institute of Diabetes, Digestive, and Kidney Diseases, Bethesda, MD 20892, United States of America

*corresponding author: [markus.hoffmann@nih.gov](mailto:markus.hoffmann@nih.gov)

**ABSTRACT**

The JAK-STAT pathway is central to cytokine signaling and controls normal physiology and disease. Aberrant activation via mutations that change amino acids in proteins of the pathway can result in diseases. While disease-centric databases like COSMIC catalog mutations in cancer, their prevalence in healthy populations remains underexplored. We systematically studied such mutations in the JAK-STAT genes by comparing COSMIC and the population-focused All of Us database. Our analysis revealed frequent mutations in all JAK and STAT domains, particularly among white females. We further identified three categories: Mutations uniquely found in All of Us that were associated with cancer in the literature but could not be found in COSMIC, underscoring COSMIC’s limitations. Mutations unique to COSMIC underline their potential as drivers of cancer due to their absence in the general population. Mutations present in both databases, e.g., JAK2^Val617Phe/V617F^ - widely recognized as a cancer driver in hematopoietic cells, but without disease associations in All of Us, raising the possibility that combinatorial SNPs might be responsible for disease development. These findings illustrate the complementarity of both databases for understanding mutation impacts and underscore the need for multi-mutation analyses to uncover genetic factors underlying complex diseases and advance personalized medicine.

**Supplementary Figure 1: The JAK-STAT pathway**


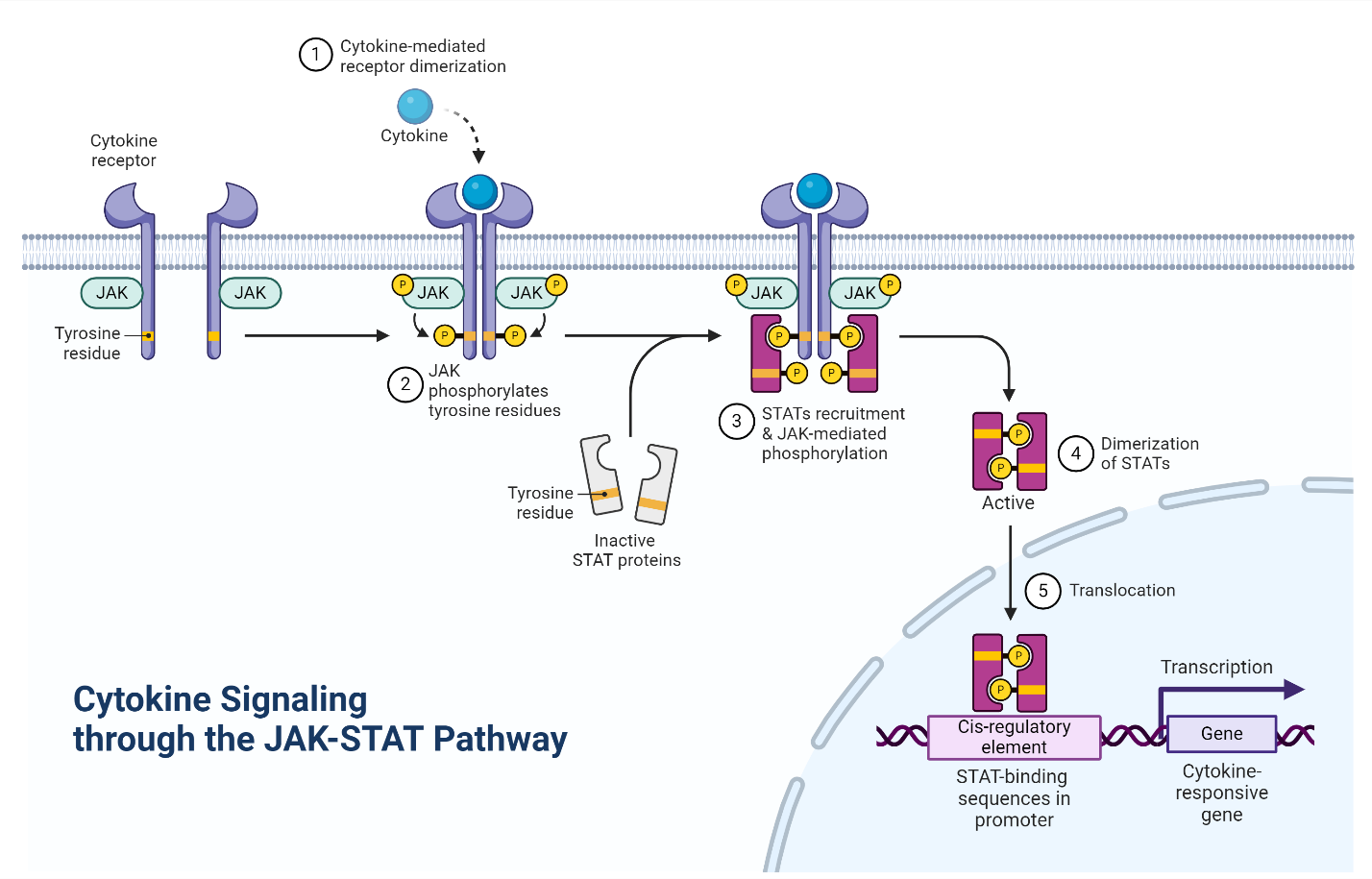


**Supplementary Figure 1:** The activation of the JAK-STAT pathway is critical in the immune system [1], leading to the regulation of immune genes, which play a significant role in the body's response to viral infections [2,3], autoimmune diseases [4], cancer [5], and a plethora of other conditions [6,7]. Following cytokine signaling, the JAK-STAT pathway facilitates the phosphorylation and dimerization of STAT transcription factors (TFs), which subsequently translocate to the nucleus to bind gamma-activated sites (GAS) motifs in cis-regulatory elements (CREs) such as promoters and enhancers [1] and regulate various immune genes (Figure 1 a, [8]). The successful and targeted binding of the STAT TF family with GAS motifs is crucial in regulating the expression levels of immune genes.

**Supplementary Figure 2: Missense mutations with at least 20 samples in All Of Us and/ or COSMIC in the coding regions of the genes STAT1, STAT2, STAT3, and STAT4**


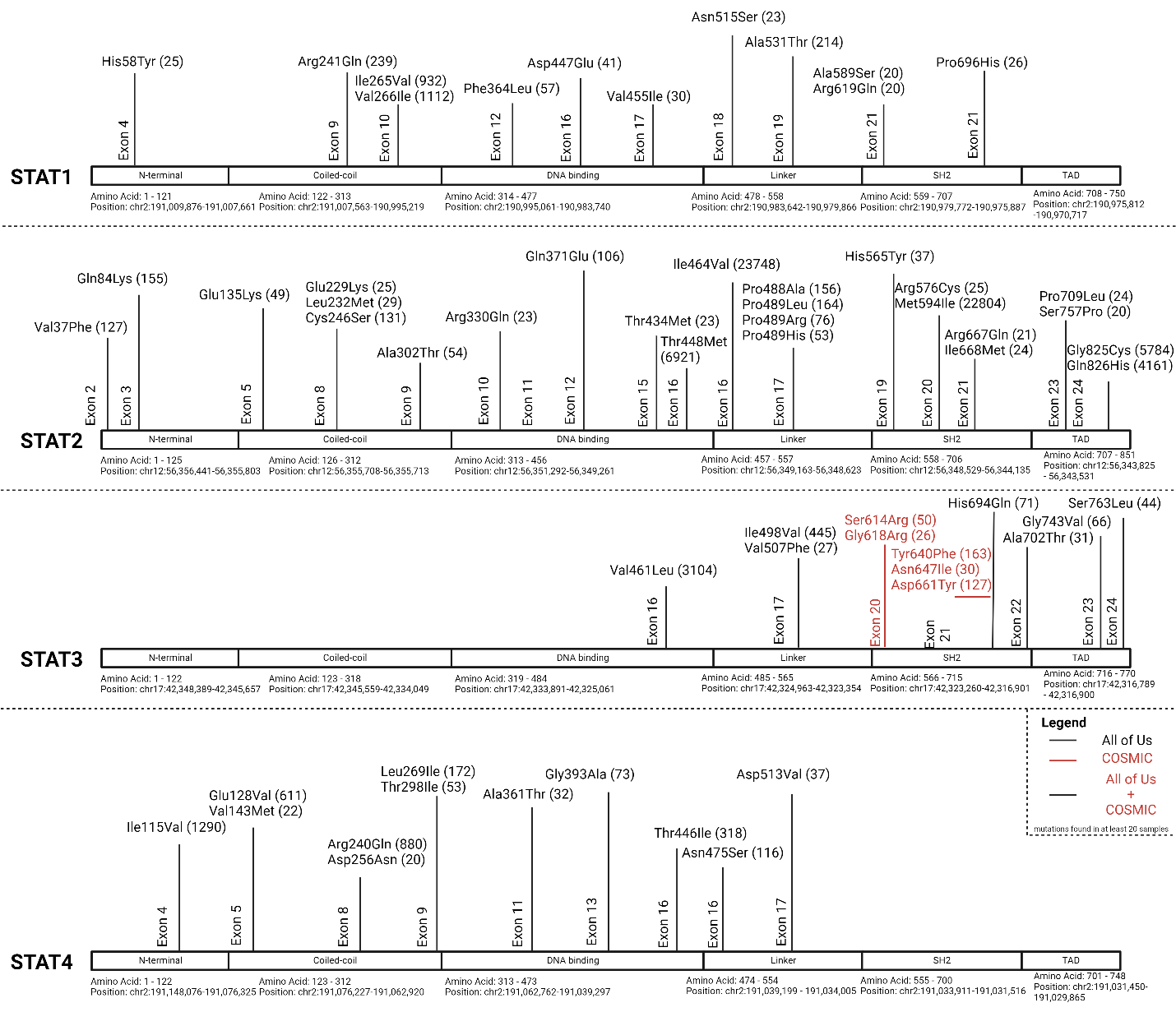


**Supplementary Figure 3: Missense mutations with at least 20 samples in All Of Us and/ or COSMIC in the coding regions of the genes STAT5A, STAT5B, STAT6**


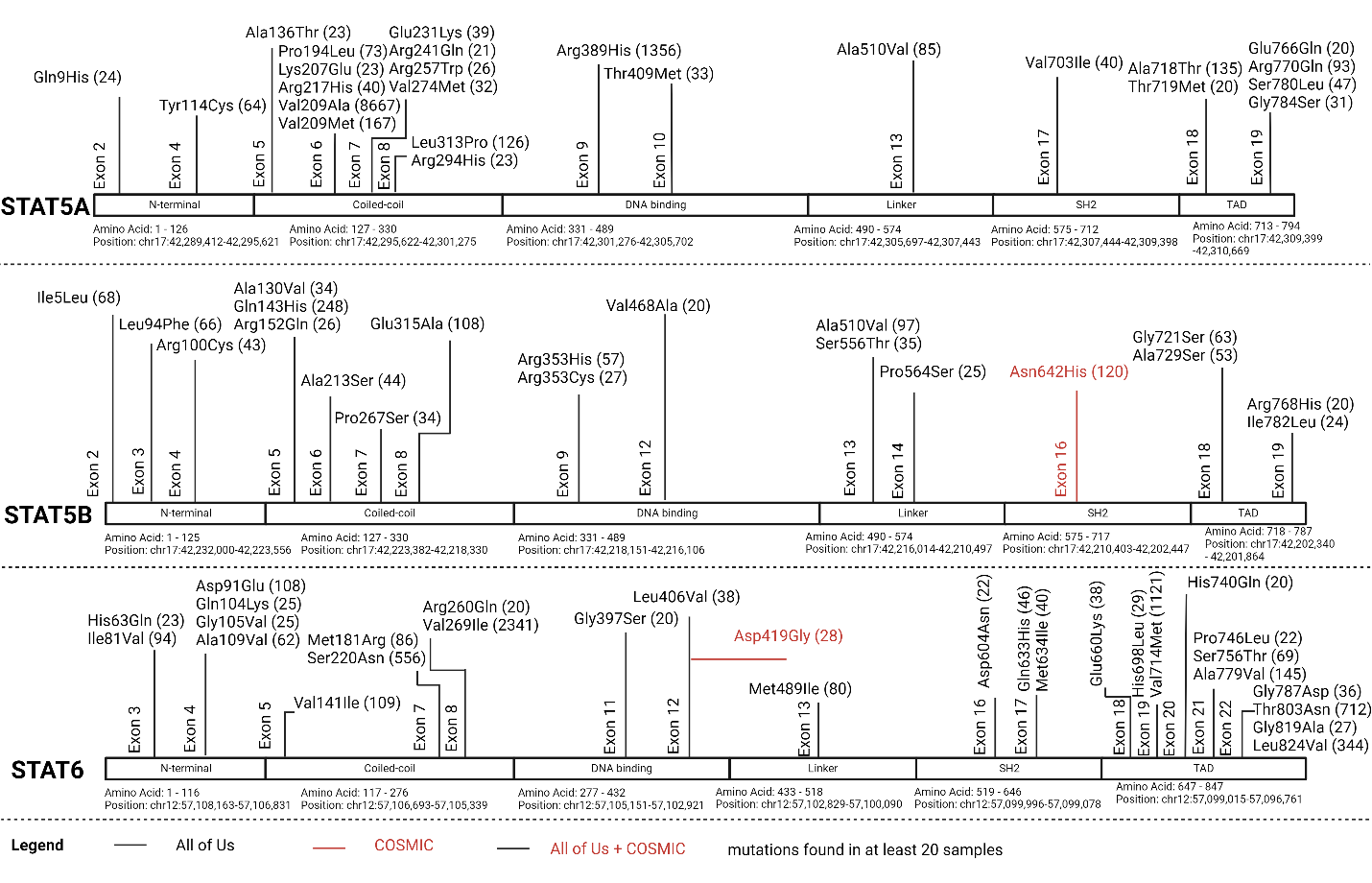


**Supplementary Figure 4: Missense mutations with at least 20 samples in All Of Us and/ or COSMIC in the coding regions of the genes JAK1, JAK2, JAK3, TYK2**


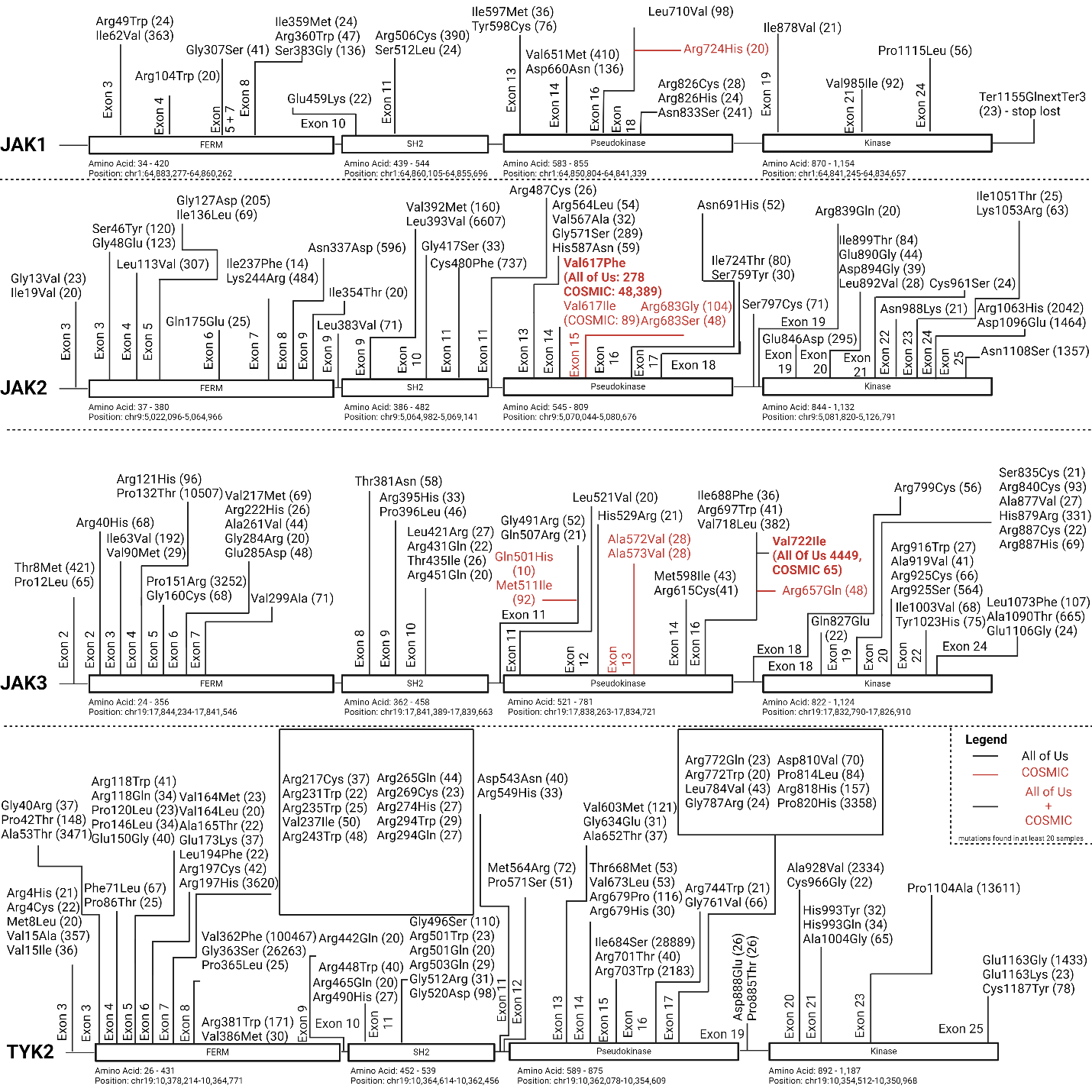


**Supplementary Table 1: Overview of JAK/STAT mutations in COSMIC and All of Us and which ones were published in the literature**

Note: If there is no entry in Disease association in AllOfUs, we did not find a condition that affects at least 25% of samples. Predominant ethnicity/sex at birth means that AllOfUs indicates this for >50% of samples.

1. STAT1

| **Amino acid substitution** | **One-letter code** | **rsID** | **Number of samples COSMIC** | **Number of samples All of Us** | **Literature reference** | **Disease association in the literature** | **Predominant ethnicity, according to AllOfUs** | **Predominant sex at birth, according to AllOfUs** | **Disease association in All of Us** |
| --- | --- | --- | --- | --- | --- | --- | --- | --- | --- |
| His58Tyr | H58Y | rs751586208 | - | 25 | - | - | Black | Male | Several conditions associated with weight (i.e., Essential hypertension, diabetes 2 Mellitus without complications) |
| Arg241Gln | R241Q | rs146273341 | - | 239 | - | - | Hispanic | Female | Essential hypertension |
| Ile265Val | I265V | rs148775168 | - | 932 | - | - | Black | Female | Essential hypertension, chest pain |
| Val266Ile | V266I | rs41473544 | - | 1112 | [9] | Polyendocrinopathy  in 5 children | White | Female | Essential hypertension, Hyperlipidemia |
| Phe364Leu | F364L | N/A | - | 52 | - | - | White | Female | Essential hypertension, Hyperlipidemia |
| Asp447Glu | D447E | N/A | - | 41 | - | - | White | Male | Essential hypertension, Hyperlipidemia, Anxiety |
| Val455Ile | V455I | rs371982540 | - | 30 | [10] | Chronic mucocutaneous candidiasis  in 1 child | Hispanic | Female | Essential hypertension, Hyperlipidemia |
| Asn515Ser | N515S | rs190269533 | - | 23 | - | - | Hispanic | Female | Essential hypertension, Hyperlipidemia, Major depression, Anxiety disorder |
| Ala531Thr | A531T | rs148573907 | - | 214 | - | - | Black | Female | Essential hypertension |
| Ala589Ser | A589S | rs745491762 | - | 20 | - | - | Black | Male | Several conditions associated with weight (i.e., Essential hypertension, diabetes 2 Mellitus without complications) |
| Arg619Gln | R619Q | rs369060692 | - | 20 | - | - | Black | Male | Essential hypertension, Hyperlipidemia, Obesity, Gastroesophageal reflux disease |
| Pro696His | P696H | rs138723664 | - | 26 | - | - | White | Female | Essential hypertension, Anemia, Disorder of bone |

1. STAT2

| **Amino acid substitution** | **One-letter code** | **rsID** | **Number of samples COSMIC** | **Number of samples All of Us** | **Literature references** | **Disease association in the literature** | **Predominant ethnicity, according to AllOfUs** | **Predominant sex at birth, according to AllOfUs** | **Disease association in All of Us** |
| --- | --- | --- | --- | --- | --- | --- | --- | --- | --- |
| Val37Phe | V37F | rs144812882 | - | 127 | - | - | Hispanic | Female | Abdominal Pain, Essential hypertension, Obesity |
| Gln84Lys | Q84K | rs150901100 | - | 155 | - | - | Hispanic | Female | Essential hypertension, Hyperlipidemia |
| Glu135Lys | E135K | rs753806117 | - | 49 | - | - | Black | Female | - |
| Glu229Lys | E229K | rs200581403 | - | 25 | - | - | White | Female | Essential hypertension, Anxiety disorder |
| Leu232Met | L232M | rs143159503 | - | 29 | - | - | White | - | Essential hypertension, Anxiety disorder, Osteoarthritis of knee |
| Cys246Ser | C246S | N/A | - | 131 | - | - | Black | Female | Essential hypertension |
| Ala302Thr | A302T | rs141205552 | - | 54 | - | - | Black | Female | Abdominal pain |
| Arg330Gln | R330Q | rs149666262 | - | 23 | - | - | - | Female | Essential hypertension, Abdominal pain |
| Gln371Glu | Q371E | rs142439434 | - | 106 | - | - | White | Female | Essential hypertension, Hyperlipidemia, Pure hypercholesterolemia, Gastroesophageal reflux disease without esophagitis |
| Thr434Met | T434M | rs146115536 | - | 23 | - | - | White | Female | Essential hypertension, Gastroesophageal reflux disease without esophagitis, Hyperlipidemia, Abdominal pain, Anxiety disorder, Benign neoplasm of colon |
| Thr448Met | T448M | rs2066815 | - | 6921 | - | - | Hispanic | Female | - |
| Ile464Val | I464V | rs2066811 | - | 23748 | - | - | Black | Female | Essential hypertension |
| Pro488Ala | P488A | rs750338004 | - | 156 | - | - | Hispanic | Female | - |
| Pro489Leu | P489L | N/A | - | 164 | - | - | Hispanic | Female | Essential hypertension, Hyperlipidemia |
| Pro489Arg | P489R | N/A | - | 76 | - | - | - | Female | Hyperlipidemia, Essential hypertension, Abdominal pain, Chest pain |
| Pro489His | P489H | N/A | - | 53 | - | - | - | Female | Essential hypertension, Obesity |
| His565Tyr | H565Y | rs143700674 | - | 37 | - | - | White | Female | Hyperlipidemia, Essential hypertension |
| Arg576Cys | R576C | rs780130229 | - | 25 | - | - | - | Female | Abdominal pain, Essential hypertension |
| Met594Ile | M594I | N/A | - | 22804 | - | - | White | Female | Essential hypertension, Hyperlipidemia |
| Arg667Gln | R667Q | rs200606416 | - | 21 | - | - | White | Female | Essential hypertension, Hyperlipidemia, Abdominal pain |
| Ile668Met | I668M | rs199528062 | - | 24 | - | - | White | Female | Essential hypertension, Hyperlipidemia, Abdominal pain, Type 2 diabetes mellitus without complication |
| Pro709Leu | P709L | rs149859580 | - | 24 | - | - | White | Female | - |
| Gln825Cys | Q825C | N/A | - | 5784 | - | - | White | Female | Essential hypertension, Hyperlipidemia |
| Gln826His | Q826H | N/A | - | 4161 | - | - | White | Female | Essential hypertension, Hyperlipidemia, Chest pain |

1. STAT3

| **Amino acid substitution** | **One-letter code** | **rsID** | **Number of samples COSMIC** | **Number of samples All of Us** | **Literature references** | **Disease association in the literature** | **Predominant ethnicity, according to AllOfUs** | **Predominant sex at birth, according to AllOfUs** | **Disease association in All of Us** |
| --- | --- | --- | --- | --- | --- | --- | --- | --- | --- |
| Val461Leu | V461L | rs149214040 | - | 3104 | - | - | Hispanic | Female | Abdominal pain, Essential hypertension |
| Ile498Val | I498V | rs146620441 | - | 445 | - | - | Black | Female | Essential hypertension, Chest pain, Obesity |
| Val507Phe | V507F | rs145786768 | - | 27 | [11] | In silicio analysis resulted that V507 is one of the most deleterious SNPs in STAT3 | - | Female | Essential hypertension, low back pain |
| Ser614Arg | S614R | N/A | 50 | - | [12] | Mixed leukemic stage | - | - | - |
| Gly618Arg | G618R | rs2081548277 | 26 | - | [13] | Lymphocyte leukemia | - | - | - |
| Gly618Arg | G618R | rs2081548277 | 26 | - | [14] | EBV-positive inflammatory follicular dendritic cell sarcoma | - | - | - |
| Gly618Arg | G618R | rs2081548277 | 26 | - | [15] | T-cell large granular lymphocytic leukemia | - | - | - |
| Tyr614Phe | Y614F | rs769031989 | 163 | - | - | - | - | - | - |
| Asn647Ile | N647I | rs770986654 | 30 | - | [16] | Large granular lymphocytic leukemia | - | - | - |
| Asn647Ile | N647I | rs770986654 | 30 | - | [15] | Large granular lymphocytic leukemia | - | - | - |
| Asn647Ile | N647I | rs770986654 | 30 | - | [17] | Review | - | - | - |
| Asn647Ile | N647I | rs770986654 | 30 | - | [18] | T-LGL leukemia | - | - | - |
| Asn647Ile | N647I | rs770986654 | 30 | - | [19] | T-large granular lymphocyte leukemia | - | - | - |
| Asp661Tyr | D661Y | rs747639500 | 127 | - | [19] | T-large granular lymphocyte leukemia | - | - | - |
| Asp661Tyr | D661Y | rs747639500 | 127 | - | [20] | Large granular lymphocyte leukemia | - | - | - |
| Asp661Tyr | D661Y | rs747639500 | 127 | - | [21] | T-Cell Large Granular Lymphocytic Leukemia and Chronic Lymphoproliferative Disorder | - | - | - |
| Asp661Tyr | D661Y | rs747639500 | 127 | - | [18] | T-LGL leukemia | - | - | - |
| Asp661Tyr | D661Y | rs747639500 | 127 | - | [22] | Large granular lymphocyte leukemia | - | - | - |
| His694Gln | H694Q | N/A | - | 71 | - | - | White | Female | Essential hypertension |
| Ala702Thr | A702T | rs747667389 | - | 31 | - | - | Hispanic | Female | Type 2 diabetes mellitus without complication |
| Gly743Val | G743V | rs151033214 | - | 66 | - | - | White | Female | Essential hypertension, Hyperlipidemia, Gastroesophageal reflux disease without esophagitis, Anxiety disorder, Obstructive sleep apnea syndrome |
| Ser763Leu | S763L | rs140604473 | - | 44 | - | - | White | Female | Essential hypertension, Hyperlipidemia, Acute pharyngitis, Anxiety disorder, Inflammatory dermatosis |

1. STAT4

| **Amino acid substitution** | **One-letter code** | **rsID** | **Number of samples COSMIC** | **Number of samples All of Us** | **Literature references** | **Disease association in the literature** | **Predominant ethnicity, according to AllOfUs** | **Predominant sex at birth, according to AllOfUs** | **Disease association in All of Us** |
| --- | --- | --- | --- | --- | --- | --- | --- | --- | --- |
| Ile115Val | I115V | rs3024839 | - | 1290 | - | - | Black | Female | Essential hypertension, Chest pain |
| Glu128Val | E128V | rs140675301 | - | 611 | [23] | Rheumatoid arthritis | White | Female | Essential hypertension, Hyperlipidemia |
| Val143Met | V143M | rs370819441 | - | 22 | - | - | White | - | Anxiety disorder, Chest pain, Diverticulosis of large intestine without diverticulitis, Essential hypertension |
| Arg240Gln | R240Q | rs61756200 | - | 880 | - | - | White | Female | Essential hypertension, Hyperlipidemia, Chest pain |
| Asp256Asn | D256N | rs771061948 | - | 20 | - | - | Black | Female | Essential hypertension, Chest pain, Type 2 diabetes mellitus without complication, Eruption |
| Leu269Ile | L269I | rs35279173 | - | 172 | - | - | White | Female | Hyperlipidemia, Essential hypertension, Chest pain, Gastroesophageal reflux disease without esophagitis, **Vitamin D deficiency, Disorder of bone, Pure hypercholesterolemia** |
| Thr298Ile | T298I | rs200982266 | - | 53 | - | - | White | Female | Hyperlipidemia, Essential hypertension |
| Ala361Thr | A361T | rs751746803 | - | 32 | - | - | - | Female | Essential hypertension, Abdominal pain, Gastroesophageal reflux disease without esophagitis, Hyperlipidemia |
| Gly393Ala | G393A | rs199633613 | - | 73 | - | - | White | Female | Hyperlipidemia, Essential hypertension, Gastroesophageal reflux disease without esophagitis, Anxiety Disorder |
| Thr446Ile | T446I | rs141331848 | - | 318 | [24] | Classic Kaposi Sarcoma | White | Female | Essential hypertension, Hyperlipidemia, Anxiety disorder |
| Asn475Ser | N475S | rs146386562 | - | 116 | - | - | Black | Female | Essential hypertension |
| Asp513Val | D513V | rs147007636 | - | 37 | - | - | Black | Female | Essential hypertension |

1. STAT5A

| **Amino acid substitution** | **One-letter code** | **rsID** | **Number of samples COSMIC** | **Number of samples All of Us** | **Literature references** | **Disease association in the literature** | **Predominant ethnicity, according to AllOfUs** | **Predominant sex at birth, according to AllOfUs** | **Disease association in All of Us** |
| --- | --- | --- | --- | --- | --- | --- | --- | --- | --- |
| Gln9His | Q9H | N/A | - | 24 | - | - | White | Male | Essential hypertension, Hyperlipidemia, Obesity |
| Tyr114Cys | Y114C | rs149721767 | - | 64 | - | - | White | Female | Essential hypertension, Hyperlipidemia, Gastroesophageal reflux disease without esophagitis |
| Ala136Thr | A136T | rs766742717 | - | 23 | - | - | Black | Female | Chest pain, Abdominal pain, Gastroesophageal reflux disease without esophagitis |
| Pro194Leu | P194L | rs200134221 | - | 73 | - | - | White | - | Hyperlipidemia, Essential hypertension, Anxiety disorder |
| Lys207Glu | K207E | rs369438268 | - | 23 | - | - | White | Female | Abdominal pain, Acute pharyngitis, Insomnia, Chest pain, Essential hypertension |
| Arg217His | R217H | rs527783523 | - | 40 | - | - | Asian | - | Hyperlipidemia |
| Val209Ala | V209A | rs2230123 | - | 8667 | - | - | Black | Female | Essential hypertension, Chest pain |
| Val209Met | V209M | rs367568442 | - | 167 | - | - | Black | Female | Essential hypertension, Chest pain, Abdominal pain |
| Glu231Lys | E231K | rs765171270 | - | 39 | - | - | - | Female | Abdominal pain, Essential hypertension, Hyperlipidemia, Chest pain, Obesity |
| Arg241Gln | R241Q | rs990723381 | - | 21 | - | - | - | Female | Anemia, Chest pain, Diarrhea, Essential hypertension, Major depression, Obesity, Vitamin D deficiency |
| Arg257Trp | R257W | rs1331665794 | - | 26 | - | - | White | Female | Essential hypertension, Hyperlipidemia, Anxiety disorder, Chest pain |
| Val274Met | V274M | rs750660966 | - | 32 | - | - | White | Female | Essential hypertension, Hyperlipidemia, Chest pain, Abdominal pain, Neck pain, Acute pharyngitis |
| Leu313Pro | L313P | rs142232293 | - | 126 | - | - | White | Female | Essential hypertension, Low back pain, Hyperlipidemia, Chest pain |
| Arg294His | R294H | rs778262335 | - | 23 | - | - | White | Male | Backache, Hyperlipidemia, Essential hypertension, Abdominal pain, Gastroesophageal reflux disease without esophagitis, Vitamin D deficiency |
| Arg389His | R389H | rs2230134 | - | 1356 | - | - | Black | Female | Essential hypertension, Chest pain |
| Thr409Met | T409M | rs143067673 | - | 33 | - | - | Asian | Female | - |
| Ala510Val | A510V | rs775536794 | - | 85 | - | - | - | - | Essential hypertension, Hyperlipidemia, Gastroesophageal reflux disease without esophagitis |
| Val703Ile | V703I | rs759275556 | - | 40 | - | - | Black | Female | Essential hypertension |
| Ala718Thr | A718T | rs200025445 | - | 135 | - | - | - | Female | - |
| Thr719Met | T719M | rs568644507 | - | 20 | - | - | White | Female | Gastroesophageal reflux disease without esophagitis, Essential hypertension, Anxiety disorder, Backache, Blood chemistry abnormal, Hyperlipidemia |
| Glu766Gln | E766Q | rs776801677 | - | 20 | - | - | White | Female | Dysuria |
| Arg770Gln | R770Q | rs149261935 | - | 93 | - | - | White | Female | Hyperlipidemia, Essential hypertension, Pure hypercholesterolemia |
| Ser780Leu | S780L | rs147384091 | - | 47 | - | - | - | Female | Essential hypertension, Hyperlipidemia, Abdominal pain, Low back pain |
| Gly784Ser | G784S | rs139614126 | - | 31 | - | - | White | Female | Abdominal pain, Dyspnea, Essential hypertension, Hyperlipidemia |

1. STAT5B

| **Amino acid substitution** | **One-letter code** | **rsID** | **Number of samples COSMIC** | **Number of samples All of Us** | **Literature references** | **Disease association in the literature** | **Predominant ethnicity, according to AllOfUs** | **Predominant sex at birth, according to AllOfUs** | **Disease association in All of Us** |
| --- | --- | --- | --- | --- | --- | --- | --- | --- | --- |
| Ile5Leu | I5L | N/A | - | 68 | - | - | Black | Female | Essential hypertension, Type 2 diabetes mellitus without complication, Dyspnea, Low back pain |
| Leu94Phe | L94F | rs199645527 | - | 66 | - | - | Asian | Female | Essential hypertension |
| Arg100Cys | R100C | rs199894785 | - | 43 | - | - | White | Female | Essential hypertension, Chest pain, Hyperlipidemia, Dyspnea, Neck pain, Abdominal pain, Vitamin D deficiency |
| Ala130Val | A130V | rs2277619 | - | 34 | - | - | Asian | Female | - |
| Gln143His | Q143H | N/A | - | 248 | - | - | Black | Female | Essential hypertension, Obesity |
| Arg162Gln | R152Q | N/A | - | 26 | - | - | Black | Female | Essential hypertension, Chest pain, Depressive disorder, Abdominal pain, Dyspnea, Hyperlipidemiam Low back pain, Type 2 diabetes mellitus without complication |
| Ala213Ser | A213S | N/A | - | 44 | - | - | White | Female | Hyperlipidemia, Low back pain, Essential hypertension, Eruption, Abdominal pain, Chest pain, Dyspnea, Backache |
| Pro267Ser | P267S | N/A | - | 34 | - | - | Asian | Female | Abdominal pain, Essential hypertension |
| Glu315Ala | E315A | rs572536541 | - | 108 | - | - | Asian | Female | - |
| Arg353His | R353H | rs143171571 | - | 57 | - | - | White | Female | Hyperlipidemia, Essential hypertension, Eruption, Inflammatory dermatosis, Low back pain |
| Arg353Cys | R353C | rs762833594 | - | 27 | - | - | Black | Female | Essential hypertension, Shoulder joint pain, Neck pain, Abdominal pain, Backache, Chest pain, Knee pain, Low back pain |
| Val468Ala | V468A | N/A | - | 20 | - | - | - | Female | Abdominal pain, Chest pain, Low back pain, Essential hypertension, Hyperlipidemia, Iron deficiency anemia, Type 2 diabetes mellitus without complication |
| Ala510Val | A510V | rs200200711 | - | 97 | - | - | White | - | Essential hypertension, Hyperlipidemia, Dyspnea, Chest pain, Low back pain, Gastroesophageal reflux disease without esophagitis, |
| Ser556Thr | S556T | N/A | - | 35 | - | - | Black | Female | Essential hypertension, Chest pain |
| Pro564Ser | P564S | N/A | - | 25 | - | - | Black | Female | - |
| Asn642His | N642H | rs938448224 | 120 | - | [25] | Chronic myeloid neoplasms with eosinophilia and/or basophilia | - | - | - |
| Asn642His | N642H | rs938448224 | 120 | - | [26] | T-Cell Prolymphocytic Leukemia with 15 cases | - | - | - |
| Asn642His | N642H | rs938448224 | 120 | - | [27] | Mycobacterial osteomyelitis in 1 child | - | - | - |
| Asn642His | N642H | rs938448224 | 120 | - | [28] | Feline Alimentary T Cell Lymphoma | - | - | - |
| Asn642His | N642H | rs938448224 | 120 | - | [29] | Review | - | - | - |
| Gly721Ser | G721S | N/A | - | 63 | - | - | - | Female | Essential hypertension, Abdominal pain, Low back pain |
| Ala729Ser | A729S | N/A | - | 53 | - | - | Asian | Female | Hyperlipidemia, Essential hypertension |
| Ala768His | A768H | N/A | - | 20 | - | - | Hispanic | Female | Hyperlipidemia, Abdominal pain, Chest pain, Essential hypertension, Edema, |
| Ile782Leu | I782L | N/A | - | 24 | - | - | Black | Male | Chest pain, Essential hypertension, Dyspnea, Type 2 diabetes mellitus without complication |

1. STAT6

| **Amino acid substitution** | **One-letter code** | **rsID** | **Number of samples COSMIC** | **Number of samples All of Us** | **Literature references** | **Disease association in the literature** | **Predominant ethnicity, according to AllOfUs** | **Predominant sex at birth, according to AllOfUs** | **Disease association in All of Us** |
| --- | --- | --- | --- | --- | --- | --- | --- | --- | --- |
| His63Gln | H63Q | N/A | - | 23 | - | - | White | Female | Essential hypertension, Hyperlipidemia, Chest pain, Eruption, Gastroesophageal reflux disease without esophagitis, Low back pain, Pain in limb |
| Ile81Val | I81V | rs761099650 | - | 94 | - | - | Hispanic | Female | Abdominal pain, Chest pain |
| Asp91Glu | D91E | N/A | - | 108 | - | - | Hispanic | Female | Abdominal pain, Chest pain |
| Gln104Lys | Q104K | rs143832217 | - | 25 | - | - | White | Female | Gastroesophageal reflux disease without esophagitis, Backache, Hyperlipidemia, Abdominal pain, Anxiety disorder, Essential hypertension, Shoulder joint pain |
| Gly105Val | G105V | rs769739267 | - | 25 | - | - | White | Female | Chronic pain, Abdominal pain, Chest pain, Gastroesophageal reflux disease, Gastroesophageal reflux disease without esophagitis |
| Ala109Val | A109V | rs570858641 | - | 62 | - | - | Black | Female | Essential hypertension, Chest pain, Low back pain, Obesity |
| Val141Ile | V141I | rs377723871 | - | 109 | - | - | White | Female | Essential hypertension, Hyperlipidemia, Chest pain, Gastroesophageal reflux disease without esophagitis |
| Met181Arg | M181R | rs3024952 | - | 86 | - | - | - | Female | Essential hypertension, Chest pain, Abdominal pain |
| Ser220Asn | S220N | rs138604557 | - | 556 | - | - | White | Female | Essential hypertension, Hyperlipidemia, Chest pain, Gastroesophageal reflux disease without esophagitis, Low back pain |
| Arg260Gln | R260Q | rs370880075 | - | 20 | - | - | White | Female | Dyspnea, Abdominal pain, Anemia, Anxiety disorder, Essential hypertension, Gastroesophageal reflux disease without esophagitis, Major depression |
| Val269Ile | V269I | rs118014438 | - | 2341 | - | - | - | Female | - |
| Gly397Ser | G397S | rs372158354 | - | 20 | - | - | White | Female | Abdominal pain, Essential hypertension, Gastroesophageal reflux disease without esophagitis, Hyperlipidemia, Low back pain, Major depression, Shoulder joint pain, Urinary tract infectious disease |
| Leu406Val | L406V | rs145406233 | - | 38 | - | - | White | Female | Dyspnea, Essential hypertension, |
| Asp419Gly | D419G | N/A | 28 | - | - | - | - | - | - |
| Met489Ile | M489I | N/A | - | 80 | - | - | Black | Female | Essential hypertension |
| Asp604Asn | D604N | rs373679457 | - | 22 | - | - | White | Female | Essential hypertension, Allergic rhinitis, Hyperlipidemia, Abdominal pain, Chest pain, Dyspnea, Low back pain |
| Gln633His | Q633H | N/A | - | 46 | - | - | White | Female | Hyperlipidemia, Essential hypertension, Obesity, Gastroesophageal reflux disease without esophagitis |
| Met634Ile | M634I | N/A | - | 40 | - | - | Black | Female | Chest pain, Dyspnea, Essential hypertension |
| Glu660Lys | E660K | rs201859128 | - | 38 | - | - | White | Female | Essential hypertension, Hyperlipidemia, |
| His698Leu | H698L | rs201585470 | - | 29 | - | - | White | Female | - |
| Val714Met | V714M | rs147655222 | - | 1121 | - | - | Black | Female | Essential hypertension, Chest pain |
| Pro746Leu | P746L | rs373268442 | - | 22 | - | - | Black | Female | Chest pain, Neck pain, Essential hypertension, |
| Ser756Thr | S756T | rs369234436 | - | 69 | - | - | Black | - | Essential hypertension, Low back pain, Chest pain |
| Ala779Val | A779V | rs145346629 | - | 145 | - | - | Black | Female | Essential hypertension, Obesity, Chest pain |
| Gly787Asp | G787D | rs369827931 | - | 36 | - | - | Black | Female | Essential hypertension |
| Thr803Asn | T803N | rs140273166 | - | 712 | - | - | Black | Female | Essential hypertension, Chest pain |
| **Gly819Ala** | G819A | rs766855555 | - | 27 | - | - | White | Female | Essential hypertension, Hyperlipidemia, Abdominal pain, Eruption, Inflammatory dermatosis, Senile hyperkeratosis |
| Leu824Val | L824V | rs146670318 | - | 344 | - | - | White | Female | Essential hypertension, Hyperlipidemia, Chest pain, |

1. JAK1

| **Amino acid substitution** | **One-letter code** | **rsID** | **Number of samples COSMIC** | **Number of samples All of Us** | **Literature references** | **Disease association in the literature** | **Predominant ethnicity, according to AllOfUs** | **Predominant sex at birth, according to AllOfUs** | **Disease association in All of Us** |
| --- | --- | --- | --- | --- | --- | --- | --- | --- | --- |
| Arg49Trp | R49W | rs754991396 | - | 24 | - | - | - | Female | Essential hypertension, Gastroesophageal reflux disease without esophagitis, Abdominal pain, Type 2 diabetes mellitus without complication |
| Ile62Val | I62V | N/A | - | 363 | - | - | White | Female | Essential hypertension, Hyperlipidemia |
| Arg104Trp | R104W | rs752389823 | - | 20 | - | - | Hispanic | Female | Abdominal pain, Chest pain, Anxiety disorder, Essential hypertension, Inflammatory dermatosis, Nausea, Neck pain |
| Gly307Ser | G307S | rs373142876 | - | 41 | - | - | White | Female | Essential hypertension, Hyperlipidemia, Low back pain, Chest pain, Vitamin D deficiency, Abdominal pain |
| Ile359Met | I359M | rs376472861 | - | 24 | - | - | White | Female | Chronic pain, Hyperlipidemia, Abdominal pain, Dyspnea, Essential hypertension, Anemia, Low back pain, Vitamin D deficiency |
| Arg360Trp | R360W | rs200049537 | - | 47 | - | - | White | Female | Hyperlipidemia, Essential hypertension |
| Ser383Gly | S383G | rs201432491 | - | 136 | - | - | White | Female | Essential hypertension, Hyperlipidemia, |
| Glu459Lys | E459K | N/A | - | 22 | - | - | Hispanic | Female | Essential hypertension, Anxiety disorder, Gastroesophageal reflux disease without esophagitis, Chest pain, Low back pain |
| Arg506Cys | R506C | rs61735631 | - | 390 | - | - | White | Female | Hyperlipidemia, Essential hypertension, Low back pain |
| Ser512Leu | S512L | rs368776025 | - | 24 | - | - | White | Female | Essential hypertension, Dyspnea, Hyperlipidemia, Blood chemistry abnormal, Gastroesophageal reflux disease without esophagitis, Pain in limb, Acute pharyngitis, Anxiety disorder, Low back pain |
| Ile597Met | I597M | rs758372896 | - | 36 | - | - | - | Female | Gastroesophageal reflux disease without esophagitis, Abdominal pain, Anxiety disorder, Essential hypertension, Low back pain |
| Tyr598Cys | Y598C | rs374269002 | - | 76 | - | - | Black | Female | Essential hypertension |
| Val651Met | V651M | rs149968614 | - | 410 | - | - | White | Female | Essential hypertension, Hyperlipidemia, Chest pain |
| Asp660Asn | D660N | rs368904859 | - | 136 | - | - | Hispanic | Female | - |
| Leu710Val | L710V | rs377757935 | - | 98 | - | - | Black | Female | Essential hypertension, Type 2 diabetes mellitus without complication, Obesity |
| Arg724His | R724H | rs780016058 | 20 | - | - | - | - | - | - |
| Arg826Cys | R826C | rs150021823 | - | 28 | - | - | Black | Female | Acute upper respiratory infection, Essential hypertension, Gastroesophageal reflux disease without esophagitis, Anemia, Chest pain, Depressive disorder, Type 2 diabetes mellitus without complication |
| Arg826His | R826H | rs199886153 | - | 24 | - | - | Hispanic | Female | Abdominal pain, Chest pain, Dyspnea, Acute pharyngitis, Dysuria, Gastroesophageal reflux disease without esophagitis, Obesity |
| Asn833Ser | N833S | rs187043211 | - | 241 | - | - | Hispanic | Female | Essential hypertension, Hyperlipidemia, |
| Ile878Val | I878V | rs553794700 | - | 21 | - | - | Asian | Female | - |
| Val985Ile | V985I | rs367582687 | - | 92 | - | - | Black | Female | Essential hypertension |
| Pro1115Leu | P1115L | rs543134894 | - | 56 | - | - | Hispanic | Female | - |
| Ter1155Gln (lost stop) | X1155Q | rs200563303 | - | 23 | - | - | Asian | Male | Essential hypertension, Hyperlipidemia, Vitamin D deficiency |

1. JAK2

| **Amino acid substitution** | **One-letter code** | **rsID** | **Number of samples COSMIC** | **Number of samples All of Us** | **Literature references** | **Disease association in the literature** | **Predominant ethnicity, according to AllOfUs** | **Predominant sex at birth, according to AllOfUs** | **Disease association in All of Us** |
| --- | --- | --- | --- | --- | --- | --- | --- | --- | --- |
| Gly13Val | G13V | rs759031245 | - | 23 | - | - | Hispanic | Female | Essential hypertension, Obesity, Abdominal pain, Chest pain, Hyperlipidemia |
| Ile19Val | I19V | rs150159583 | - | 20 | - | - | - | Female | Abdominal pain, Backache, Chest pain, Major depression |
| Ser46Tyr | S46Y | rs138655335 | - | 120 | - | - | Black | Female | Essential hypertension, Chest pain, |
| Gly48Glu | G48E | rs143227399 | - | 123 | - | - | White | Female | Hyperlipidemia, Essential hypertension, Gastroesophageal reflux disease without esophagitis, Shoulder joint pain |
| Leu113Val | L113V | rs143103233 | - | 307 | - | - | White | Female | Essential hypertension, Hyperlipidemia, Chest pain, Abdominal pain, Low back pain |
| Gly127Asp | G127D | rs56118985 | - | 205 | [30] | Ph-negative MPN | Asian | Female | - |
| Ile136Leu | I136L | N/A | - | 69 | - | - | Black | Female | Essential hypertension, Chest pain |
| Gln175Glu | Q175E | rs756128160 | - | 25 | - | - | - | Female | Hyperlipidemia, Abdominal pain, Anxiety disorder, Essential hypertension |
| Ile237Phe | I237F | rs376125987 | - | 14 | - | - | White | Female | Abdominal pain, Hyperlipidemia, Anxiety disorder, Chest pain, Backache, Obesity |
| Lys244Arg | K244R | rs62637625 | - | 484 | - | - | Black | Female | Essential hypertension, Chest pain |
| Asn337Asp | N337D | rs149683525 | - | 596 | - | - | Black | Female | Essential hypertension, Chest pain |
| Ile354Thr | I354T | rs371907546 | - | 20 | - | - | White | - | Chronic pain, Low back pain |
| Leu383Val | L383V | rs143124074 | - | 71 | - | - | Black | Female | Essential hypertension |
| Val392Met | V392M | rs200018153 | - | 160 | - | - | Asian | Female | - |
| Leu393Val | L393V | rs2230723 | - | 6607 | - | - | - | Female | Essential hypertension, Chest pain |
| Gly417Ser | G417S | rs190968273 | - | 33 | - | - | - | Female | Essential hypertension |
| Cys480Phe | C480F | rs62637623 | - | 737 | - | - | Black | Female | Essential hypertension |
| Arg487Cys | R487C | rs764423560 | - | 26 | - | - | White | Female | Essential hypertension, Hyperlipidemia, Chest pain, Gastroesophageal reflux disease without esophagitis, Anxiety disorder, Backache, Diarrhea |
| Arg564Leu | R564L | rs368927897 | - | 54 | [31] | Myelodysplastic Syndromes | White | Female | Essential hypertension, Hyperlipidemia, Abdominal pain, Chest pain, Low back pain |
| Arg564Leu | R564L | rs368927897 | - | 54 | [32] | Comparative study | White | Female | Essential hypertension, Hyperlipidemia, Abdominal pain, Chest pain, Low back pain |
| Val567Ala | V567A | rs587778408 | - | 32 | - | - | Hispanic | Female | Essential hypertension, Abdominal pain, Acute pharyngitis, Hyperlipidemia |
| Gly571Ser | G571S | rs139504737 | - | 289 | [33] | Acute lymphoblastic leukemia | - | Female | Essential hypertension, Hyperlipidemia, Abdominal pain, |
| Gly571Ser | G571S | rs139504737 | - | 289 | [34] | Thrombocythemia | - | Female | Essential hypertension, Hyperlipidemia, Abdominal pain, |
| Gly571Ser | G571S | rs139504737 | - | 289 | [35] | Myeloproliferative neoplasms | - | Female | Essential hypertension, Hyperlipidemia, Abdominal pain, |
| Gly571Ser | G571S | rs139504737 | - | 289 | [36] | - | - | Female | Essential hypertension, Hyperlipidemia, Abdominal pain, |
| His587Asn | H587N | rs149705816 |  | 59 | [32] | Comparative study | Black | Female | Chest pain, Essential hypertension |
| Val617Phe | V617F | rs77375493 | 48389 | 278 | [37] | Myeloproliferative neoplasms | White | Female | Essential hypertension, Hyperlipidemia, Chest pain, Dyspnea, Essential thrombocythemia, Abdominal pain, Pain in limb, Blood chemistry abnormal, Anemia |
| Val617Phe | V617F | rs77375493 | 48389 | 278 | [38] | Polycythemia Vera | White | Female | Essential hypertension, Hyperlipidemia, Chest pain, Dyspnea, Essential thrombocythemia, Abdominal pain, Pain in limb, Blood chemistry abnormal, Anemia |
| Val617Phe | V617F | rs77375493 | 48389 | 278 | [39] | Chronic Thromboembolic Pulmonary Hypertension | White | Female | Essential hypertension, Hyperlipidemia, Chest pain, Dyspnea, Essential thrombocythemia, Abdominal pain, Pain in limb, Blood chemistry abnormal, Anemia |
| Val617Phe | V617F | rs77375493 | 48389 | 278 | [40] | - | White | Female | Essential hypertension, Hyperlipidemia, Chest pain, Dyspnea, Essential thrombocythemia, Abdominal pain, Pain in limb, Blood chemistry abnormal, Anemia |
| Val617Phe | V617F | rs77375493 | 48389 | 278 | [41] | Myeloproliferative neoplasms | White | Female | Essential hypertension, Hyperlipidemia, Chest pain, Dyspnea, Essential thrombocythemia, Abdominal pain, Pain in limb, Blood chemistry abnormal, Anemia |
| Val617Phe | V617F | rs77375493 | 48389 | 278 | [42] | L-positive myelofibrosis | White | Female | Essential hypertension, Hyperlipidemia, Chest pain, Dyspnea, Essential thrombocythemia, Abdominal pain, Pain in limb, Blood chemistry abnormal, Anemia |
| Val617Phe | V617F | rs77375493 | 48389 | 278 | [43] | Myeloproliferative neoplasms, co-occurring with V617F | White | Female | Essential hypertension, Hyperlipidemia, Chest pain, Dyspnea, Essential thrombocythemia, Abdominal pain, Pain in limb, Blood chemistry abnormal, Anemia |
| Val617Phe | V617F | rs77375493 | 48389 | 278 | [44] | Myeloid Sarcoma of the Breast | White | Female | Essential hypertension, Hyperlipidemia, Chest pain, Dyspnea, Essential thrombocythemia, Abdominal pain, Pain in limb, Blood chemistry abnormal, Anemia |
| Val617Phe | V617F | rs77375493 | 48389 | 278 | [45] | Myeloproliferative neoplasm | White | Female | Essential hypertension, Hyperlipidemia, Chest pain, Dyspnea, Essential thrombocythemia, Abdominal pain, Pain in limb, Blood chemistry abnormal, Anemia |
| Val617Phe | V617F | rs77375493 | 48389 | 278 | [46] | Myeloproliferative neoplasm | White | Female | Essential hypertension, Hyperlipidemia, Chest pain, Dyspnea, Essential thrombocythemia, Abdominal pain, Pain in limb, Blood chemistry abnormal, Anemia |
| Val617Phe | V617F | rs77375493 | 48389 | 278 | [47] | - | White | Female | Essential hypertension, Hyperlipidemia, Chest pain, Dyspnea, Essential thrombocythemia, Abdominal pain, Pain in limb, Blood chemistry abnormal, Anemia |
| Val617Phe | V617F | rs77375493 | 48389 | 278 | [48] | Myeloproliferative disorders | White | Female | Essential hypertension, Hyperlipidemia, Chest pain, Dyspnea, Essential thrombocythemia, Abdominal pain, Pain in limb, Blood chemistry abnormal, Anemia |
| Val617Ile | V617I | rs77375493 | 89 | - | [32] | Comparative study  (did not predict V617F!!) | White | Female | Essential hypertension, Hyperlipidemia, Chest pain, Dyspnea, Essential thrombocythemia, Abdominal pain, Pain in limb, Blood chemistry abnormal, Anemia |
| Val617Ile | V617I | rs77375493 | 89 | - | [49] | 20 different amino acids were tried | White | Female | Essential hypertension, Hyperlipidemia, Chest pain, Dyspnea, Essential thrombocythemia, Abdominal pain, Pain in limb, Blood chemistry abnormal, Anemia |
| Val617Ile | V617I | rs77375493 | 89 | - | [50] | Does not result In myeloproliferative disorder | White | Female | Essential hypertension, Hyperlipidemia, Chest pain, Dyspnea, Essential thrombocythemia, Abdominal pain, Pain in limb, Blood chemistry abnormal, Anemia |
| Arg683Gly | R683G | rs1057519721 | 104 | - | [51] | Lymphoblastic leukemia cell lines | - | - | - |
| Arg683Gly | R683G | rs1057519721 | 104 | - | [52] | Acute Lymphoblastic Leukemia and Netherton Syndrome | - | - | - |
| Arg683Gly | R683G | rs1057519721 | 104 | - | [53] | Thrombocythemia | - | - | - |
| Arg683Gly | R683G | rs1057519721 | 104 | - | [54] | Lymphoblastic lymphoma | - | - | - |
| Arg683Gly | R683G | rs1057519721 | 104 | - | [55] | B-precursor acute lymphoblastic leukemia | - | - | - |
| Arg683Gly | R683G | rs1057519721 | 104 | - | [56] | Acute lymphoblastic leukemia | - | - | - |
| Arg683Ser | R683S | N/A | 48 | - | [57] | ABL1-like B-lineage acute lymphoblastic leukemia | - | - | - |
| Arg683Ser | R683S | N/A | 48 | - | [58] | Basic science | - | - | - |
| Arg683Ser | R683S | N/A | 48 | - | [59] | Chronic Eosinophilic Leukemia and Myelodysplastic/ Myeloproliferative | - | - | - |
| Arg683Ser | R683S | N/A | 48 | - | [60] | Philadelphia-like B-lineage acute lymphoblastic leukemia | - | - | - |
| Arg683Ser | R683S | N/A | 48 | - | [61] | Erythroid leukemia | - | - | - |
| Asn691His | N691H | rs151160183 | - | 52 | - | - | Black | Male | Essential hypertension, Low back pain |
| Ile724Thr | I724T | rs372254348 | - | 80 | [62] | Myeloproliferative neoplasms | - | Female | Essential hypertension, Abdominal pain, Dyspnea |
| Ser759Tyr | S759Y | rs766524586 | - | 30 | - | - | - | Female | Abdominal pain, Essential hypertension |
| Ser797Cys | S797C | rs201992086 | - | 71 | - | - | White | Female | Essential hypertension, Hyperlipidemia |
| Arg839Gln | R839Q | rs747381013 | - | 20 | - | - | White | Female | Essential hypertension, Chest pain, Gastroesophageal reflux disease without esophagitis, Obesity, Anemia, Dyspnea, Hyperlipidemia, Morbid obesity, Anxiety disorder, |
| Glu846Asp | E846D | N/A | - | 295 | [63] | Hereditary erythrocytosis with megakaryocytic atypia | White | Female | Essential hypertension, Hyperlipidemia, Chest pain |
| Glu846Asp | E846D | N/A | - | 295 | [64] | Familial erythrocytosis | White | Female | Essential hypertension, Hyperlipidemia, Chest pain |
| Glu846Asp | E846D | N/A | - | 295 | [65] | Erythrocytosis | White | Female | Essential hypertension, Hyperlipidemia, Chest pain |
| Ile899Thr | I899T | rs200282557 | - | 84 | - | - | - | Female | Essential hypertension |
| Glu890Gly | E890G | rs368599778 | - | 44 | - | - | White | Female | Essential hypertension, Anemia, Anxiety disorder, Chest pain, Hyperlipidemia |
| Asp894Gly | D894G | rs757780497 | - | 39 | - | - | Hispanic | Female | Essential hypertension, Obesity |
| Leu892Val | L892V | rs201551707 | - | 28 | - | - | White | Female | Chest pain, |
| Cys961Ser | C961S | N/A | - | 24 | - | - | Black | Female | Essential hypertension, Dyspnea, Obesity |
| Asn988Lys | N988K | N/A | - | 21 | - | - | Black | Female | Obesity, Essential hypertension, Hyperlipidemia, Chest pain, Osteoarthritis of knee, Type 2 diabetes mellitus without complication, |
| Ile1051Thr | I1051T | rs375671491 | - | 25 | - | - | - | Female | Essential hypertension |
| Lys1053Arg | K1053R | rs142094756 | - | 63 | - | - | - | Female | Essential hypertension |
| Arg1063His | R1063H | rs41316003 | - | 2042 | [42] | L-positive myelofibrosis | White | Female | Essential hypertension, Hyperlipidemia, Chest pain |
| Arg1063His | R1063H | rs41316003 | - | 2042 | [43] | Myeloproliferative neoplasms, co-occurring with V617F | White | Female | Essential hypertension, Hyperlipidemia, Chest pain |
| Arg1063His | R1063H | rs41316003 | - | 2042 | [63] | Familial erythrocytosis | White | Female | Essential hypertension, Hyperlipidemia, Chest pain |
| Arg1063His | R1063H | rs41316003 | - | 2042 | [66] | Familial Ischemic Stroke | White | Female | Essential hypertension, Hyperlipidemia, Chest pain |
| Asp1096Glu | D1096E | N/A | - | 1464 | - | - | Black | Female | Essential hypertension, Chest pain |
| Asn1108Ser | N1108S | rs142269166 | - | 1357 | [67] | Polycythemia | White | Female | Essential hypertension, Hyperlipidemia |
| Asn1108Ser | N1108S | rs142269166 | - | 1357 | [42] | Myelofibrosis | White | Female | Essential hypertension, Hyperlipidemia |
| Asn1108Ser | N1108S | rs142269166 | - | 1357 | [68] | Transformation of myeloproliferative neoplasms into acute myeloid leukemia | White | Female | Essential hypertension, Hyperlipidemia |

1. JAK3

| **Amino acid substitution** | **One-letter code** | **rsID** | **Number of samples COSMIC** | **Number of samples All of Us** | **Literature references** | **Disease association in the literature** | **Predominant ethnicity, according to AllOfUs** | **Predominant sex at birth, according to AllOfUs** | **Disease association in All of Us** |
| --- | --- | --- | --- | --- | --- | --- | --- | --- | --- |
| Thr8Met | T8M | rs145500023 | - | 421 | - | - | White | Female | Essential hypertension, Hyperlipidemia, Anxiety disorder, Gastroesophageal reflux disease without esophagitis |
| Pro12Leu | P12L | rs56061056 | - | 65 | - | - | White | - | Essential hypertension, Hyperlipidemia |
| Arg40His | R40H | rs56384680 | - | 68 | - | - | - | Female | Essential hypertension, Hyperlipidemia, Gastroesophageal reflux disease without esophagitis, Anxiety disorder, Dyspnea, Low back pain |
| Ile63Val | I63V | rs144405201 | - | 192 | - | - | White | Female | Essential hypertension, Hyperlipidemia, Chest pain, Low back pain, Gastroesophageal reflux disease without esophagitis, Dyspnea, Pure hypercholesterolemia, Abdominal pain |
| Val90Met | V90M | rs1016346013 | - | 29 | - | - | - | Female | Acute upper respiratory infection, Abdominal pain, Essential hypertension, |
| Arg121His | R121H | rs143586866 | - | 96 | - | - | White | Female | Essential hypertension, Acute upper respiratory infection, Hyperlipidemia |
| Pro132Thr | P132T | rs3212723 | - | 10507 | [69] | Acute megakaryoblastic leukemia | Black | Female | Essential hypertension, Chest pain |
| Pro132Thr | P132T | rs3212723 | - | 10507 | [70] | Acute megakaryoblastic leukemia | Black | Female | Essential hypertension, Chest pain |
| Pro132Thr | P132T | rs3212723 | - | 10507 | [71] | Head and Neck Cancer | Black | Female | Essential hypertension, Chest pain |
| Pro132Thr | P132T | rs3212723 | - | 10507 | [72] | Ameloblastoma | Black | Female | Essential hypertension, Chest pain |
| Val217Met | V217M | rs202167678 | - | 69 | - | - | White | - | Hyperlipidemia, Essential hypertension, Obesity, Chest pain, Gastroesophageal reflux disease without esophagitis, Pure hypercholesterolemia, Shoulder joint pain, Vitamin D deficiency |
| Arg222His | R222H | rs199868795 | - | 26 | [73] | Chronic active Epstein-Barr virus infection | Asian | Male | - |
| Ala261Val | A261V | rs777830679 | - | 44 | - | - | Black | Female | Essential hypertension, Abdominal pain, Chest pain, Acute upper respiratory infection, Backache, Dyspnea, Low back pain, Obesity |
| Gly284Arg | G284R | N/A | - | 20 | - | - | Black | Female | Essential hypertension, Low back pain, Type 2 diabetes mellitus without complication, Backache, Chest pain, Hyperlipidemia, Anemia, Gastroesophageal reflux disease without esophagitis |
| Glu285Asp | E285D | N/A | - | 48 | - | - | - | Female | Essential hypertension, Chest pain, Dyspnea, Gastroesophageal reflux disease without esophagitis, Major depression, |
| Val299Ala | V299A | rs571404212 | - | 71 | - | - | Black | Female | Essential hypertension, Anemia, Obesity |
| Thr381Asn | T381N | rs373046546 | - | 58 | - | - | Black | Female | Essential hypertension, Obesity |
| Arg395His | R395H | rs143038064 | - | 33 | - | - | Black | Female | - |
| Pro396Leu | P396L | rs149047410 | - | 46 | - | - | Asian | Male | - |
| Leu421Arg | L421R | rs535740127 | - | 27 | - | - | - | Female | - |
| Arg431Gln | R431Q | rs144953325 | - | 22 | - | - | - | Female | Essential hypertension |
| Thr435Ile | T435I | rs199706172 | - | 26 | - | - | White | Female | Anxiety disorder, Dyspnea, Gastroesophageal reflux disease without esophagitis |
| Arg451Gln | R451Q | rs145751599 | - | 20 | - | - | Black | Male | Essential hypertension |
| Gly491Arg | G491R | rs200112185 | - | 52 | - | - | Black | - | Essential hypertension, Low back pain, Abdominal pain, Anxiety disorder, Hyperlipidemia |
| Gln401His | Q401H | N/A | 10 | - | - | - | - | - | - |
| Gln507Arg | Q507R | rs140690573 | - | 21 | - | - | White | Female | Eruption, Hyperlipidemia, Chest pain, Essential hypertension |
| Met511Ile | M511I | N/A | 92 | - | [74] | T-cell acute lymphoblastic leukemia | - | - | - |
| Met511Ile | M511I | N/A | 92 | - | [75] | JAK3 inhibitor | - | - | - |
| Met511Ile | M511I | N/A | 92 | - | [76] | JAK3 inhibitor | - | - | - |
| Met511Ile | M511I | N/A | 92 | - | [77] | Acute Myeloid Leukemia | - | - | - |
| Met511Ile | M511I | N/A | 92 | - | [78] | Primary T-cell Lymphomas | - | - | - |
| Leu521Val | L521V | rs55666418 | - | 20 | - | - | White | - | Essential hypertension, Acute sinusitis, Gastroesophageal reflux disease without esophagitis, Hyperlipidemia, Low back pain, Malaise, Obesity, Acute bronchitis |
| His529Arg | H529R | rs142805245 | 21 | - | - | - | White | Female | Dyspnea, Anxiety disorder, Migraine |
| Ala572Val | A572V | rs121913504 | 28 | - | [79] | Basic science | - | - | - |
| Ala572Val | A572V | rs121913504 | 28 | - | [80] | Basic science | - | - | - |
| Ala572Val | A572V | rs121913504 | 28 | - | [81] | T-cell malignancies | - | - | - |
| Ala572Val | A572V | rs121913504 | 28 | - | [82] | Basic science | - | - | - |
| Ala572Val | A572V | rs121913504 | 28 | - | [83] | JAK3-mutation-positive leukemia | - | - | - |
| Ala573Val | A573V | rs2147686240 | 28 | - | [84] | Extranodal nasal-type natural killer cell lymphoma | - | - | - |
| Ala573Val | A573V | rs2147686240 | 28 | - | [82] | Basic science | - | - | - |
| Ala573Val | A573V | rs2147686240 | 28 | - | [85] | Extranodal NK/T-Cell Lymphoma | - | - | - |
| Ala573Val | A573V | rs2147686240 | 28 | - | [86] | Natural killer/T-cell lymphoma | - | - | - |
| Ala573Val | A573V | rs2147686240 | 28 | - | [87] | JAK3 inhibitor | - | - | - |
| Met598Ile | M598I | N/A | - | 43 | - | - | White | Female | Anxiety disorder |
| Arg615Cys | R615C | N/A | - | 41 | - | - | Asian | Female | Hyperlipidemia |
| Arg657Gln | R657Q | rs758959409 | 48 | - | [88] | T-cell prolymphocytic leukemia | - | - | - |
| Arg657Gln | R657Q | rs758959409 | 48 | - | [89] | Transient myeloproliferative disorder and acute megakaryoblastic leukemia accompanying Down syndrome | - | - | - |
| Ile688Phe | I888F | N/A | - | 36 | - | - | Asian | - | - |
| Val722Ile | V722I | rs3213409 | 48 | 4449 | [90] | Soft tissue sarcoma | White | Female | Essential hypertension, Hyperlipidemia, Chest pain |
| Val722Ile | V722I | rs3213409 | 48 | 4449 | [84] | Extranodal nasal-type natural killer cell lymphoma | White | Female | Essential hypertension, Hyperlipidemia, Chest pain |
| Val722Ile | V722I | rs3213409 | 48 | 4449 | [91] | Renal cell carcinoma | White | Female | Essential hypertension, Hyperlipidemia, Chest pain |
| Val722Ile | V722I | rs3213409 | 48 | 4449 | [92] | T-cell lymphoma (SOCS1 involvement) | White | Female | Essential hypertension, Hyperlipidemia, Chest pain |
| Val722Ile | V722I | rs3213409 | 48 | 4449 | [93] | HYPER FUNCTIONING PARATHYROID CYST | White | Female | Essential hypertension, Hyperlipidemia, Chest pain |
| Val722Ile | V722I | rs3213409 | 48 | 4449 | [94] | Acute lymphoblastic leukemia | White | Female | Essential hypertension, Hyperlipidemia, Chest pain |
| Val722Ile | V722I | rs3213409 | 48 | 4449 | [95] | Idiopathic erythrocytosis | White | Female | Essential hypertension, Hyperlipidemia, Chest pain |
| Arg799Cys | R799C | rs201241352 | - | 56 | - | - | - | Female | - |
| Gln827Glu | Q827E | rs144683649 | - | 22 | - | - | White | Female | Essential hypertension, Hyperlipidemia, Chest pain, Shoulder joint pain |
| Ser835Cys | S835C | rs201966394 | - | 21 | - | - | - | Female | Essential hypertension, Dyspnea, Hyperlipidemia, Dysuria, Finding related to pregnancy, Low back pain |
| Arg840Cys | R840C | rs200077579 | - | 93 | [96] | Cytotoxic T Lymphocyte Antigen-4-Dependent Immune Dysregulation Syndrome | White | - | Essential hypertension, Hyperlipidemia, Gastroesophageal reflux disease without esophagitis, Chest pain, Chronic pain |
| Ala877Val | A877V | rs201869359 | - | 27 | - | - | White | Female | Essential hypertension, Anxiety disorder, Abdominal pain, Hyperlipidemia |
| His879Arg | H879R | rs3179893 | - | 331 | - | - | Black | Female | Essential hypertension |
| Arg887Cys | R887C | rs759015510 | - | 22 | - | - | - | Female | Dyspnea, Abdominal pain, Chest pain, Hyperlipidemia, Essential hypertension, Anemia, Asthma, Blood chemistry abnormal |
| Arg887His | R887H | rs148688786 | - | 69 | - | - | White | Female | Essential hypertension, Hyperlipidemia, Chest pain |
| Arg916Trp | R916W | rs375807308 | - | 27 | - | - | Black | Male | Essential hypertension, Chest pain, |
| Ala919Val | A919V | rs767424476 | - | 41 | - | - | Black | Female | Essential hypertension, Abdominal pain, Chest pain, Obesity, Low back pain, Neck pain, Hyperlipidemia, Nicotine dependence |
| Arg925Cys | R925C | rs149452625 | - | 66 | - | - | White | Female | Essential hypertension, Hyperlipidemia, Chronic pain |
| Arg925Ser | R925S | rs149452625 | - | 564 | [91] | Cell renal cell carcinoma | Hispanic | Female | Essential hypertension, Hyperlipidemia |
| Ile1003Val | I1003V | rs137901277 | - | 68 | - | - | - | Female | Essential hypertension, Hyperlipidemia, Gastroesophageal reflux disease without esophagitis, Shoulder joint pain, Chest pain, Pure hypercholesterolemia, Pain in limb |
| Tyr1023His | Y1023H | rs145260622 | - | 75 | - | - | White | Female | Essential hypertension, Anxiety disorder, Hyperlipidemia |
| Leu1073Phe | L1073F | rs200580168 | - | 107 | - | - | White | Female | Essential hypertension, Hyperlipidemia, |
| Ala1090Thr | A1090T | rs144968714 | - | 665 | - | - | Black | Female | Essential hypertension, Chest pain, |
| Glu1106Gly | E1106G | rs374152339 | - | 24 | - | - | White | - | Essential hypertension |

1. TYK2

| **Amino acid substitution** | **One-letter code** | **rsID** | **Number of samples COSMIC** | **Number of samples All of Us** | **Literature references** | **Disease association in the literature** | **Predominant ethnicity, according to AllOfUs** | **Predominant sex at birth, according to AllOfUs** | **Disease association in All of Us** |
| --- | --- | --- | --- | --- | --- | --- | --- | --- | --- |
| Arg4His | R4H | rs12720343 | - | 21 | - | - | - | Male | Essential hypertension, Hyperlipidemia, Cough, Abdominal pain, Chest pain, Knee pain, Low back pain, Chronic kidney disease, Gastroesophageal reflux disease without esophagitis |
| Arg4Cys | R4C | rs368801193 | - | 22 | - | - | - | Female | Chest pain, Abdominal pain, Low back pain |
| Met8Leu | M8L | N/A | - | 20 | - | - | Black | Female | Essential hypertension, Chest pain, Abdominal pain, Pain in limb, Pain in right lower limb |
| Val15Ala | V15A | rs144960992 | - | 357 | - | - | White | Female | Hyperlipidemia, Essential hypertension, Gastroesophageal reflux disease without esophagitis |
| Val15Ile | V15I | rs374780145 | - | 36 | - | - | White | Female | Essential hypertension, Abdominal pain, Hyperlipidemia |
| Gly40Arg | G40R | N/A | - | 37 | - | - | Black | - | Essential hypertension |
| Pro42Thr | P42T | N/A | - | 148 | - | - | White | Female | Hyperlipidemia, Essential hypertension |
| Ala53Thr | A53T | rs55762744 | - | 3471 | - | - | White | Female | Essential hypertension, Hyperlipidemia |
| Phe71Leu | F71L | N/A | - | 67 | - | - |  |  |  |
| Pro86Thr | P86T | rs141466711 | - | 25 | - | - | Hispanic | Female | Anxiety disorder, Chest pain |
| Arg118Trp | R118W | rs138742402 | - | 41 | - | - | Black | Female | Chest pain, Low back pain, Abdominal pain, Essential hypertension, Obesity |
| Arg118Gln | R118Q | rs369530676 | - | 34 | - | - | - | Female | Essential hypertension, Type 2 diabetes mellitus without complication, Abdominal pain, Hyperlipidemia, Low back pain, Type 2 diabetes mellitus |
| Pro120Leu | P120L | N/A | - | 23 | - | - | White | Female | Hyperlipidemia, Abdominal pain, Chest pain, Essential hypertension, Shoulder joint pain, Benign neoplasm of skin, Dyspnea |
| Pro146Leu | P146L | rs137904701 | - | 34 | - | - | White | Female | Essential hypertension, Hyperlipidemia, Anxiety disorder, Dyspnea, Gastroesophageal reflux disease without esophagitis, Low back pain, Major depression, Shoulder joint pain |
| Glu150Gly | E150G | rs200718118 | - | 40 | - | - | White | - | Essential hypertension, Dyspnea, Hyperlipidemia, Anxiety disorder, Chest pain |
| Val164Met | V164M | rs531355933 | - | 23 | - | - | - | Female | Essential hypertension, Abdominal pain, Hyperlipidemia, Shoulder joint pain, Acute pharyngitis, |
| Val164Leu | V164L | rs531355933 | - | 20 | - | - | - | Female | Essential hypertension, Abdominal pain, Hyperlipidemia, Shoulder joint pain, Acute pharyngitis |
| Ala165Thr | A165T | rs370420133 | - | 22 | - | - | White | - | Essential hypertension, Hyperlipidemia, Blood chemistry abnormal, Vitamin D deficiency |
| Glu173Lys | E173K | N/A | - | 37 | - | - | Black | - | Essential hypertension |
| Leu194Phe | L194F | rs373574074 | - | 22 | - | - | White | Female | Essential hypertension, Hyperlipidemia, Chest pain, Obstructive sleep apnea syndrome, Chronic pain, Pain in left knee, Sleep apnea |
| Arg197Cys | R197C | rs376427383 | - | 42 | - | - | - | Female | Chest pain, Essential hypertension, Knee pain, Obesity, Shoulder joint pain, Backache |
| Arg197His | R197H | rs12720263 | - | 3620 | - | - | Black | Female | Essential hypertension, Chest pain, |
| Arg217Cys | R217C | rs754414161 | - | 37 | - | - | - | Female | Essential hypertension, Chest pain, Dyspnea |
| Arg231Trp | R231W | rs201917359 | - | 22 | [97] | Rheumatoid arthritis | Asian | Female | - |
| Arg231Trp | R231W | rs201917359 | - | 22 | [98] | T-cell lymphopenia | Asian | Female | - |
| Arg235Trp | R235W | rs767776173 | - | 25 | - | - | Hispanic | Female | Chest pain, Backache |
| Val237Ile | V237I | N/A | - | 50 | - | - | - | Female | Chest pain, Hyperlipidemia |
| Arg243Trp | R243W | rs200003143 | - | 48 | - | - | - | Female | Chest pain, Anxiety disorder, Essential hypertension, Gastroesophageal reflux disease without esophagitis, Abdominal pain, Hyperlipidemia, Neck pain |
| Arg265Gln | R265Q | rs775315394 | - | 44 | - | - | Black | Female | Essential hypertension |
| Arg269Cys | R269C | rs368109068 | - | 23 | - | - | - | Female | - |
| Arg274His | R274H | rs371937276 | - | 27 | - | - | Black | Female | Essential hypertension |
| Arg294Trp | R294W | rs367656874 | - | 29 | - | - | Hispanic | Female | - |
| Arg294Gln | R294Q | rs201811978 | - | 27 | - | - | - | Female | Essential hypertension, Hyperlipidemia, Obesity, Epigastric pain, Gastroesophageal reflux disease without esophagitis |
| Val362Phe | V362F | rs2304256 | - | 100467 | [99] | Basic science | White | Female | Essential hypertension, Hyperlipidemia, |
| Val362Phe | V362F | rs2304256 | - | 100467 | [100] | Tuberculosis | White | Female | Essential hypertension, Hyperlipidemia, |
| Val362Phe | V362F | rs2304256 | - | 100467 | [101] | Systemic sclerosis | White | Female | Essential hypertension, Hyperlipidemia, |
| Gly363Ser | G363S | rs2304255 | - | 26263 | - | - | White | Female | Essential hypertension, Hyperlipidemia |
| Pro365Leu | P365L | rs373929549 | - | 25 | - | - | Hispanic | Female | Dyspnea, Obesity, Essential hypertension, Gastroesophageal reflux disease without esophagitis |
| Arg381Trp | R381W | rs201240289 | - | 171 | - | - | White | - | Hyperlipidemia, Essential hypertension, Chest pain |
| Val386Met | V386M | rs55956017 | - | 30 | - | - | Asian | Female | - |
| Arg442Gln | R442Q | rs2304254 | - | 20 | - | - | White | - | Dyspnea, Hyperlipidemia, Essential hypertension |
| Arg448Trp | R448W | N/A | - | 40 | - | - | - | - | Low back pain, Diarrhea, Essential hypertension, Gastroesophageal reflux disease without esophagitis, Anemia, Backache, Nausea and vomiting |
| Arg465Gln | R465Q | rs771922681 | - | 20 | - | - | Black | Female | Essential hypertension |
| Arg490His | R490H | rs369615833 | - | 27 | - | - | White | Female | Chest pain |
| Gly496Ser | G496S | rs202072909 | - | 110 | - | - | White | Female | Essential hypertension, Hyperlipidemia, Chest pain |
| Arg501Trp | R501W | rs756944840 | - | 23 | - | - | White | Female | Chest pain, Hyperlipidemia, Essential hypertension, Shoulder joint pain, Abdominal pain, Disorder of bone, Gastroesophageal reflux disease without esophagitis |
| Arg501Gln | R501Q | rs200643906 | - | 20 | - | - | - | Female | Abdominal pain, Low back pain, Anxiety disorder, Blood chemistry abnormal, Essential hypertension |
| Arg503Gln | R503Q | rs541104349 | - | 29 | - | - | Black | Female | Essential hypertension |
| Gly512Arg | G512R | N/A | - | 31 | - | - | Asian | Female | Essential hypertension, Abdominal pain, Gastroesophageal reflux disease without esophagitis, Obesity |
| Gly520Asp | G520D | rs142576987 | - | 98 | - | - | White | Female | - |
| Asp543Asn | D543N | rs747616008 | - | 40 | - | - | Black | - | Essential hypertension, Chest pain, Abdominal pain, Anemia, Dyspnea |
| Arg549His | R549H | rs536181491 | - | 33 | - | - | White | Female | Essential hypertension, Hyperlipidemia |
| Met564Arg | M564R | rs72561470 | - | 72 | - | - | Black | - | Essential hypertension, Abdominal pain, |
| Pro571Ser | P571S | rs867249402 | - | 51 | - | - | Black | Female | Abdominal pain, Chest pain, Essential hypertension, |
| Val603Met | V603M | rs140594440 | - | 121 | - | - | - | Female | Essential hypertension, |
| Gly634Glu | G634E | rs189471343 | - | 31 | [102] | Mycobacterial disease | Asian | Female | Chest pain |
| Ala652Thr | A652T | rs144290894 | - | 37 | - | - | Asian | Female | Essential hypertension |
| Thr668Met | T668M | rs146430107 | - | 53 | - | - | - | Female | Essential hypertension, Low back pain, Obesity |
| Val673Leu | V673L | N/A | - | 53 | - | - | White | Female | Chronic pain, Dysuria, Essential hypertension, Hyperlipidemia, Depressive disorder, Vitamin D deficiency |
| Arg679Pro | R679P | N/A | - | 116 | - | - | Hispanic | Female | Essential hypertension, Chest pain, Hyperlipidemia, |
| Arg679His | R679H | N/A | - | 30 | - | - | White | Female | Low back pain, Essential hypertension, Neck pain, Disorder of bone, Headache, Hyperlipidemia, Obesity, Allergic rhinitis, Fatigue, Insomnia |
| Ile684Ser | I684S | rs12720356 | - | 28889 | [100] | Tuberculosis | White | Female | Essential hypertension, Hyperlipidemia, Chest pain |
| Ile684Ser | I684S | rs12720356 | - | 28889 | [101] | Systemic sclerosis | White | Female | Essential hypertension, Hyperlipidemia, Chest pain |
| Ile684Ser | I684S | rs12720356 | - | 28889 | [103] | Basic science; Autoimmune diseases | White | Female | Essential hypertension, Hyperlipidemia, Chest pain |
| Ile684Ser | I684S | rs12720356 | - | 28889 | [104] | Basic science | White | Female | Essential hypertension, Hyperlipidemia, Chest pain |
| Ile684Ser | I684S | rs12720356 | - | 28889 | [105] | Psoriasis-protective | White | Female | Essential hypertension, Hyperlipidemia, Chest pain |
| Ile684Ser | I684S | rs12720356 | - | 28889 | [106] | Protect against rheumatoid arthritis and autoimmunity | White | Female | Essential hypertension, Hyperlipidemia, Chest pain |
| Arg701Thr | R701T | rs200791116 | - | 40 | - | - | Asian | Female | - |
| Arg703Trp | R703W | rs55882956 | - | 2183 | [97] | Protect against rheumatoid arthritis | Hispanic | Female | Essential hypertension |
| Arg744Trp | R744W | rs142676100 | - | 21 | - | - | Black | Female | Cough, Chest pain, Gastroesophageal reflux disease, Acute pharyngitis, Asthma, Low back pain, Pain in limb, Abdominal pain |
| Gly761Val | G761V | rs201335603 | - | 66 | [107] | Oncogenic | White | Female | - |
| Gly761Val | G761V | rs201335603 | - | 66 | [108] | Primary acute lymphoblastic leukemia | White | Female | - |
| Arg772Gln | R772Q | rs148823525 | - | 23 | - | - | - | Female | Essential hypertension, Hyperlipidemia, Anxiety disorder, Nausea, Diarrhea, Major depression, Obesity, Osteoarthritis |
| Arg772Trp | R772W | rs755089851 | - | 20 | - | - | White | Female | Essential hypertension, Anemia, Eruption, Gastroesophageal reflux disease without esophagitis, Hyperlipidemia |
| Leu784Val | L784V | rs759014184 | - | 43 | - | - | - | Female | Essential hypertension, Hyperlipidemia, Chest pain, Obesity, Type 2 diabetes mellitus without complication, Anxiety disorder, Headache, Dyspnea |
| Gly787Arg | G787R | N/A | - | 24 | - | - | White | Female | Hyperlipidemia, Dyspnea, Essential hypertension, Anxiety disorder, Chest pain |
| Asp810Val | D810V | rs371070470 | - | 70 | - | - | White | Female | Essential hypertension, Hyperlipidemia, Chest pain, |
| Pro814Leu | P814L | rs143743593 | - | 84 | - | - | White | Female | Chest pain, Essential hypertension, Gastroesophageal reflux disease without esophagitis, Hyperlipidemia, |
| Arg818His | R818H | rs145437969 | - | 157 | - | - | Black | Female | Essential hypertension, Abdominal pain |
| Pro820His | P820H | rs34046749 | - | 3358 | - | - | Black | Female | Essential hypertension, Chest pain |
| Pro885Thr | P885T | N/A | - | 26 | - | - | White | - | Dyspnea, Hyperlipidemia, Essential hypertension, Senile hyperkeratosis, Obstructive sleep apnea syndrome, Chest pain |
| Asp888Glu | D888E | N/A | - | 26 | - | - | White | Female | Essential hypertension, Hyperlipidemia, Type 2 diabetes mellitus without complication, |
| Ala928Val | A928V | rs35018800 | - | 2334 | [102] | Mycobacterial disease | White | Female | Essential hypertension, Hyperlipidemia, Chest pain |
| Ala928Val | A928V | rs35018800 | - | 2334 | [101] | Systemic sclerosis | White | Female | Essential hypertension, Hyperlipidemia, Chest pain |
| Ala928Val | A928V | rs35018800 | - | 2334 | [106] | Protect against rheumatoid arthritis and autoimmunity | White | Female | Essential hypertension, Hyperlipidemia, Chest pain |
| Cys966Gly | C966G | N/A | - | 22 | - | - | Hispanic | Female | Hyperlipidemia |
| His993Tyr | H993Y | rs201397594 | - | 32 | - | - | Asian | Female | - |
| His993Gln | H993Q | N/A | - | 34 | - | - | - | - | Essential hypertension, Chest pain, Hyperlipidemia, Low back pain, Headache |
| Ala1004Gly | A1004G | rs202110875 | - | 65 | - | - | Black | Female | Essential hypertension, Dyspnea |
| Pro1104Ala | P1104A | rs34536443 | - | 13611 | [102] | Mycobacterial disease | White | Female | Essential hypertension, Hyperlipidemia, Chest pain |
| Pro1104Ala | P1104A | rs34536443 | - | 13611 | [109] | Autoimmune disease therapy | White | Female | Essential hypertension, Hyperlipidemia, Chest pain |
| Pro1104Ala | P1104A | rs34536443 | - | 13611 | [110] | Tuberculosis | White | Female | Essential hypertension, Hyperlipidemia, Chest pain |
| Glu1163Gly | E1163G | rs55886939 | - | 1433 | - | - | White | Female | Essential hypertension, Hyperlipidemia, Chest pain |
| Glu1163Lys | E1163K | N/A | - | 23 | - | - | - | Female | Chest pain, Essential hypertension, Dyspnea, Anemia, Gastroesophageal reflux disease without esophagitis, Headache, Hyperlipidemia |
| Cys1187Tyr | C1187Y | rs200932305 | - | 78 | - | - | Hispanic | Female | Essential hypertension, Abdominal pain, Chest pain, Hyperlipidemia |

**REFERENCES**

1. Hu X, Li J, Fu M, Zhao X, Wang W. The JAK/STAT signaling pathway: from bench to clinic. Signal Transduct Target Ther. 2021;6: 402.

2. Lee HK, Jung O, Hennighausen L. JAK inhibitors dampen activation of interferon-stimulated transcription of ACE2 isoforms in human airway epithelial cells. Commun Biol. 2021;4: 654.

3. Hoffmann M, Willruth L-L, Dietrich A, Lee HK, Knabl L, Trummer N, et al. Blood transcriptomics analysis offers insights into variant-specific immune response to SARS-CoV-2. Sci Rep. 2024;14: 1–11.

4. Banerjee S, Biehl A, Gadina M, Hasni S, Schwartz DM. JAK–STAT signaling as a target for inflammatory and autoimmune diseases: Current and future prospects. Drugs. 2017;77: 521–546.

5. Brooks AJ, Putoczki T. JAK-STAT signalling pathway in cancer. Cancers (Basel). 2020;12: 1971.

6. O’Shea JJ, Schwartz DM, Villarino AV, Gadina M, McInnes IB, Laurence A. The JAK-STAT pathway: Impact on human disease and therapeutic intervention. Annu Rev Med. 2015;66: 311–328.

7. Villarino AV, Kanno Y, O’Shea JJ. Mechanisms and consequences of Jak–STAT signaling in the immune system. Nat Immunol. 2017;18: 374–384.

8. Morris R, Kershaw NJ, Babon JJ. The molecular details of cytokine signaling via the JAK/STAT pathway. Protein Sci. 2018;27: 1984–2009.

9. Uzel G, Sampaio EP, Lawrence MG, Hsu AP, Hackett M, Dorsey MJ, et al. Dominant gain-of-function STAT1 mutations in FOXP3 wild-type immune dysregulation-polyendocrinopathy-enteropathy-X-linked-like syndrome. J Allergy Clin Immunol. 2013;131: 1611–1623.

10. Blanco Lobo P, Lei W-T, Pelham SJ, Guisado Hernández P, Villaoslada I, de Felipe B, et al. Biallelic TRAF3IP2 variants causing chronic mucocutaneous candidiasis in a child harboring a STAT1 variant. Pediatr Allergy Immunol. 2021;32: 1804–1812.

11. Ajith A, Subbiah U. In silico prediction of deleterious non-synonymous SNPs in *STAT3*. Asian Biomed (Res Rev News). 2023;17: 185–199.

12. Yan Y, Olson TL, Nyland SB, Feith DJ, Loughran TP Jr. Emergence of a STAT3 mutated NK clone in LGL leukemia. Leuk Res Rep. 2015;4: 4–7.

13. Kim D, Park G, Huuhtanen J, Ghimire B, Rajala H, Moriggl R, et al. STAT3 activation in large granular lymphocyte leukemia is associated with cytokine signaling and DNA hypermethylation. Leukemia. 2021;35: 3430–3443.

14. Ramsey MC, Sabatini PJB, Watson G, Chawla T, Ko M, Sakhdari A. Case Report: Identification of a novel STAT3 mutation in EBV-positive inflammatory follicular dendritic cell sarcoma. Front Oncol. 2023;13. doi:10.3389/fonc.2023.1266897

15. Kristensen T, Larsen M, Rewes A, Frederiksen H, Thomassen M, Møller MB. Clinical relevance of sensitive and quantitative STAT3 mutation analysis using next-generation sequencing in T-cell large granular Lymphocytic leukemia. J Mol Diagn. 2014;16: 382–392.

16. Koskela HLM, Eldfors S, Ellonen P, van Adrichem AJ, Kuusanmäki H, Andersson EI, et al. Somatic*STAT3*mutations in large granular Lymphocytic leukemia. N Engl J Med. 2012;366: 1905–1913.

17. Sakata-Yanagimoto M, Enami T, Yokoyama Y, Chiba S. Disease‐specific mutations in mature lymphoid neoplasms: Recent advances. Cancer Sci. 2014;105: 623–629.

18. Shen M. A case report of T-LGL leukemia-associated pure red cell aplasia harboring STAT3, TNFAIP3, and KMT2D mutation. Transl Cancer Res. 2023;12: 1054–1059.

19. Cheon H, Xing JC, Moosic KB, Ung J, Chan VW, Chung DS, et al. Genomic landscape of TCRαβ and TCRγδ T-large granular lymphocyte leukemia. Blood. 2022;139: 3058–3072.

20. Olson KC, Moosic KB, Jones MK, Larkin PMK, Olson TL, Toro MF, et al. Large granular lymphocyte leukemia serum and corresponding hematological parameters reveal unique cytokine and sphingolipid biomarkers and associations with STAT3 mutations. Cancer Med. 2020;9: 6533–6549.

21. Rivero A, Mozas P, Jiménez L, López-Guerra M, Colomer D, Bataller A, et al. Clinicobiological characteristics and outcomes of patients with T-cell large granular Lymphocytic leukemia and chronic lymphoproliferative disorder of natural killer cells from a single institution. Cancers (Basel). 2021;13: 3900.

22. Tanahashi T, Sekiguchi N, Matsuda K, Takezawa Y, Ito T, Kobayashi H, et al. Cell size variations of large granular lymphocyte leukemia: Implication of a small cell subtype of granular lymphocyte leukemia with STAT3 mutations. Leuk Res. 2016;45: 8–13.

23. Saevarsdottir S, Stefansdottir L, Sulem P, Thorleifsson G, Ferkingstad E, Rutsdottir G, et al. Multiomics analysis of rheumatoid arthritis yields sequence variants that have large effects on risk of the seropositive subset. Ann Rheum Dis. 2022;81: 1085–1095.

24. Aavikko M, Kaasinen E, Nieminen JK, Byun M, Donner I, Mancuso R, et al. Whole-genome sequencing identifies*STAT4*as a putative susceptibility gene in classic Kaposi sarcoma. J Infect Dis. 2015;211: 1842–1851.

25. Yin CC, Tam W, Walker SM, Kaur A, Ouseph MM, Xie W, et al. *STAT5B* mutations in myeloid neoplasms differ by disease subtypes but characterize a subset of chronic myeloid neoplasms with eosinophilia and/or basophilia. Haematologica. 2023;109. doi:10.3324/haematol.2023.284311

26. Hu Z, Medeiros LJ, Xu M, Yuan J, Peker D, Shao L, et al. T-cell prolymphocytic leukemia with t(X;14)(q28;Q11.2): A clinicopathologic study of 15 cases. Am J Clin Pathol. 2023;159: 325–336.

27. Kobets AJ, Ahmad S, Boyke A, Oriko D, Holland R, Eisenberg R, et al. STAT5b gain-of-function disease in a child with mycobacterial osteomyelitis of the skull: rare presentation of an emerging disease entity. Childs Nerv Syst. 2023;39: 2071–2077.

28. Freiche V, Couronné L, Bruneau J, Hermine O. Comment on kieslinger et al. A recurrent STAT5BN642H driver mutation in feline alimentary T cell lymphoma. Cancers 2021, 13, 5238. Cancers (Basel). 2022;14: 4593.

29. Groh M, Fenwarth L, Labro M, Boudry A, Fournier E, Wemeau M, et al. Involvement of the JAK‐STAT pathway in the molecular landscape of tyrosine kinase fusion‐negative hypereosinophilic syndromes: A nationwide CEREO study. Am J Hematol. 2024;99: 1108–1118.

30. Wang Z, Tian X, Ma J, Zhang Y, Ta W, Duan Y, et al. Clinical laboratory characteristics and gene mutation spectrum of *Ph*‐negative MPN patients with atypical variants of *JAK2*, *MPL*, or *CALR*. Cancer Med. 2024;13. doi:10.1002/cam4.7123

31. Delio M, Bryke C, Mendez L, Joseph L, Jassim S. JAK2 mutations are rare and diverse in myelodysplastic syndromes: Case series and review of the literature. Hematol Rep. 2023;15: 73–87.

32. Lee T-S, Ma W, Zhang X, Kantarjian H, Albitar M. Structural effects of clinically observed mutations in JAK2 exons 13-15: comparison with V617F and exon 12 mutations. BMC Struct Biol. 2009;9: 58.

33. Lin M, Nebral K, Gertzen CGW, Ganmore I, Haas OA, Bhatia S, et al. JAK2 p.G571S in B-cell precursor acute lymphoblastic leukemia: a synergizing germline susceptibility. Leukemia. 2019;33: 2331–2335.

34. Panovska-Stavridis I, Eftimov A, Ivanovski M, Pivkova-Veljanovska A, Cevreska L, Hermouet S, et al. Essential thrombocythemia associated with germline JAK2 G571S variant and somatic CALR type 1 mutation. Clin Lymphoma Myeloma Leuk. 2016;16: e55–e57.

35. Bahar B, Barton K, Kini AR. The role of the Exon 13 G571S JAK2 mutation in myeloproliferative neoplasms. Leuk Res Rep. 2016;6: 27–28.

36. Alghasham N, Alnouri Y, Abalkhail H, Khalil S. Detection of mutations in *JAK2* exons 12–15 by Sanger sequencing. Int J Lab Hematol. 2016;38: 34–41.

37. Zhang Y, Zhao Y, Liu Y, Zhang M, Zhang J. New advances in the role of *JAK2* V617F mutation in myeloproliferative neoplasms. Cancer. 2024 [cited 16 Sep 2024]. doi:10.1002/cncr.35559

38. Haji Paiman NS, Mat Nasir N, Miptah HN, Saidon N, Abdul Monir M. Challenges in diagnosing polycythemia Vera in primary care: A 55-year-old Malaysian woman with atypical presentation. Am J Case Rep. 2024;25. doi:10.12659/ajcr.944202

39. Eichstaedt CA, Verweyen J, Halank M, Benjamin N, Fischer C, Mayer E, et al. Myeloproliferative diseases as possible risk factor for development of chronic thromboembolic pulmonary hypertension—A genetic study. Int J Mol Sci. 2020;21: 3339.

40. Carlos JAEG, Lima K, Rego EM, Costa-Lotufo LV, Machado-Neto JA. The survivin/XIAP suppressant YM155 impairs clonal growth and induces apoptosis in JAK2V617F cells. Hematol Transfus Cell Ther. 2024 [cited 16 Sep 2024]. doi:10.1016/j.htct.2024.05.012

41. Bourrienne M-C, Loyau S, Faille D, Gay J, Akhenak S, Farkh C, et al. Impaired fibrinolysis in JAK2V617F-related myeloproliferative neoplasms. J Thromb Haemost. 2024 [cited 16 Sep 2024]. doi:10.1016/j.jtha.2024.07.031

42. Schulze S, Stengel R, Jaekel N, Wang S-Y, Franke G-N, Roskos M, et al. Concomitant and noncanonical *JAK2* and *MPL* mutations in *JAK2*V617F‐ and *MPLW*515 L‐positive myelofibrosis. Genes Chromosomes Cancer. 2019;58: 747–755.

43. Mambet C, Babosova O, Defour J-P, Leroy E, Necula L, Stanca O, et al. Cooccurring JAK2 V617F and R1063H mutations increase JAK2 signaling and neutrophilia in myeloproliferative neoplasms. Blood. 2018;132: 2695–2699.

44. Pace M, Guadagno E, Russo D, Gencarelli A, Carlea A, Di Spiezio A, et al. Myeloid sarcoma of the breast as blast phase of JAK2-mutated (Val617Phe Exon 14p) essential thrombocythemia: A case report and a systematic literature review. Pathobiology. 2023;90: 123–130.

45. Patchell D, Keohane C, O’Shea S, Langabeer SE. Incidence and impact of non-canonical JAK2 p.(Val617Phe) mutations in myeloproliferative neoplasm molecular diagnostics. J Clin Pathol. 2024; jcp-2023-209276.

46. Choi DC, Messali N, Uda NR, Abu-Zeinah G, Kermani P, Yabut MM, et al. JAK2V617F impairs lymphoid differentiation in myeloproliferative neoplasms. Leukemia. 2024 [cited 16 Sep 2024]. doi:10.1038/s41375-024-02388-3

47. Liosi M-E, Krimmer SG, Newton AS, Dawson TK, Puleo DE, Cutrona KJ, et al. Selective Janus kinase 2 (JAK2) pseudokinase ligands with a diaminotriazole core. J Med Chem. 2020;63: 5324–5340.

48. Veitia RA, Innan H. Pathogenic “germline” variants associated with myeloproliferative disorders in apparently normal individuals: Inherited or acquired genetic alterations? Clin Genet. 2022;101: 371–374.

49. Dusa A, Staerk J, Elliott J, Pecquet C, Poirel HA, Johnston JA, et al. Substitution of pseudokinase domain residue val-617 by large non-polar amino acids causes activation of JAK2. J Biol Chem. 2008;283: 12941–12948.

50. Brooks SA, Luty SB, Lai HY, Morse SJ, Nguyen TK, Royer LR, et al. JAK2V617I results in cytokine hypersensitivity without causing an overt myeloproliferative disorder in a mouse transduction–transplantation model. Exp Hematol. 2016;44: 24-29.e1.

51. Tomoyasu C, Imamura T, Tomii T, Yano M, Asai D, Goto H, et al. Copy number abnormality of acute lymphoblastic leukemia cell lines based on their genetic subtypes. Int J Hematol. 2018;108: 312–318.

52. Skoczen S, Stepien K, Mlynarski W, Centkowski P, Kwiecinska K, Korostynski M, et al. Genetic signature of acute lymphoblastic leukemia and netherton syndrome co-incidence—first report in the literature. Front Oncol. 2020;9. doi:10.3389/fonc.2019.01477

53. Arai A, Yoshimitsu M, Otsuka M, Ito Y, Miyazono T, Nakano N, et al. Identification of putative noncanonical driver mutations in patients with essential thrombocythemia. Eur J Haematol. 2023;110: 639–647.

54. Roncero AM, López-Nieva P, Cobos-Fernández MA, Villa-Morales M, González-Sánchez L, López-Lorenzo JL, et al. Contribution of JAK2 mutations to T-cell lymphoblastic lymphoma development. Leukemia. 2016;30: 94–103.

55. Hassan NM, Abdellateif MS, Radwan EM, Hameed SA, Desouky EDE, Kamel MM, et al. Prognostic significance of CRLF2 overexpression and JAK2 mutation in Egyptian pediatric patients with B-precursor acute lymphoblastic leukemia. Clin Lymphoma Myeloma Leuk. 2022;22: e376–e385.

56. Carreño-Tarragona G, Varghese LN, Sebastián E, Gálvez E, Marín-Sánchez A, López-Muñoz N, et al. A typical acute lymphoblastic leukemia JAK2 variant, R683G, causes an aggressive form of familial thrombocytosis when germline. Leukemia. 2021;35: 3295–3298.

57. Gupta DG, Varma N, Sreedharanunni S, Abdulkadir SA, Naseem S, Sachdeva MUS, et al. ‘Evaluation of adverse prognostic gene alterations & MRD positivity in BCR::ABL1-like B-lineage acute lymphoblastic leukaemia patients, in a resource-constrained setting. Br J Cancer. 2023;129: 143–152.

58. Abraham BG, Haikarainen T, Vuorio J, Girych M, Virtanen AT, Kurttila A, et al. Molecular basis of JAK2 activation in erythropoietin receptor and pathogenic JAK2 signaling. Sci Adv. 2024;10. doi:10.1126/sciadv.adl2097

59. Krah NM, Miotke L, Li P, Patel JL, Bowen AR, Pomicter AD, et al. JAK2 R683S mutation resulting in dual diagnoses of chronic eosinophilic leukemia and myelodysplastic/myeloproliferative overlap syndrome. J Natl Compr Canc Netw. 2023;21: 1218–1223.

60. Gupta DG, Varma N, Kumar A, Naseem S, Sachdeva MUS, Sreedharanunni S, et al. Genomic and proteomic characterization of Philadelphia‐like B‐lineage acute lymphoblastic leukemia: A report of Indian patients. Cancer. 2023;129: 1217–1226.

61. Xu RZ, Karsan A, Xu Z, Berry BR. A rare de novo pure erythroid leukemia with JAK2 R683S mutation. Ann Hematol. 2022;101: 921–922.

62. Puli’uvea C, Immanuel T, Green TN, Tsai P, Shepherd PR, Kalev-Zylinska ML. Insights into the role of JAK2-I724T variant in myeloproliferative neoplasms from a unique cohort of New Zealand patients. Hematology. 2024;29. doi:10.1080/16078454.2023.2297597

63. Kapralova K, Horvathova M, Pecquet C, Fialova Kucerova J, Pospisilova D, Leroy E, et al. Cooperation of germ line JAK2 mutations E846D and R1063H in hereditary erythrocytosis with megakaryocytic atypia. Blood. 2016;128: 1418–1423.

64. Tun PWW, Buka RJ, Graham J, Dyer P. Heterozygous, germline *JAK2* E846D substitution as the cause of familial erythrocytosis. Br J Haematol. 2022;198: 923–926.

65. Maaziz N, Garrec C, Airaud F, Bobée V, Contentin N, Cayssials E, et al. Germline JAK2 E846D substitution as the cause of erythrocytosis? Genes (Basel). 2023;14: 1066.

66. Ilinca A, Martinez-Majander N, Samuelsson S, Piccinelli P, Truvé K, Cole J, et al. Whole-exome sequencing in 22 young ischemic stroke patients with familial clustering of stroke. Stroke. 2020;51: 1056–1063.

67. Oliveira e Costa A, Barreira A, Cunha M, Salvador F. Polycythemia and JAK2 variant N1108S: cause-and-effect or coincidence? Hematol Transfus Cell Ther. 2023. doi:10.1016/j.htct.2023.01.006

68. Benton CB, Boddu PC, DiNardo CD, Bose P, Wang F, Assi R, et al. Janus kinase 2 variants associated with the transformation of myeloproliferative neoplasms into acute myeloid leukemia. Cancer. 2019;125: 1855–1866.

69. Riera L, Lasorsa E, Bonello L, Sismondi F, Tondat F, Di Bello C, et al. Description of a novel Janus kinase 3 P132A mutation in acute megakaryoblastic leukemia and demonstration of previously reported Janus kinase 3 mutations in normal subjects. Leuk Lymphoma. 2011;52: 1742–1750.

70. Walters DK, Mercher T, Gu T-L, O’Hare T, Tyner JW, Loriaux M, et al. Activating alleles of JAK3 in acute megakaryoblastic leukemia. Cancer Cell. 2006;10: 65–75.

71. Guerrero-Preston R, Lawson F, Rodriguez-Torres S, Noordhuis MG, Pirini F, Manuel L, et al. *JAK3* variant, immune signatures, DNA methylation, and social determinants linked to survival racial disparities in head and neck cancer patients. Cancer Prev Res (Phila). 2019;12: 255–270.

72. González-González R, López-Verdín S, Lavalle-Carrasco J, Molina-Frechero N, Isiordia-Espinoza M, Carreón-Burciaga RG, et al. Current concepts in ameloblastoma-targeted therapies in B-raf proto-oncogene serine/threonine kinase V600E mutation: Systematic review. World J Clin Oncol. 2020;11: 31–42.

73. Zhong L, Wang W, Ma M, Gou L, Tang X, Song H. Chronic active Epstein–Barr virus infection as the initial symptom in a Janus kinase 3 deficiency child. Medicine (Baltimore). 2017;96: e7989.

74. Yuan S, Wang X, Hou S, Guo T, Lan Y, Yang S, et al. PHF6 and JAK3 mutations cooperate to drive T-cell acute lymphoblastic leukemia progression. Leukemia. 2022;36: 370–382.

75. Su W, Chen Z, Liu M, He R, Liu C, Li R, et al. Design, synthesis and structure-activity relationship studies of pyrido[2,3-d]pyrimidin-7-ones as potent Janus Kinase 3 (JAK3) covalent inhibitors. Bioorg Med Chem Lett. 2022;64: 128680.

76. Li S, Si H, Song X, Lei C, He X, Wang J, et al. Discovery of hexahydrofuro[3,2-*b*]furans as new kinase-selective and orally bioavailable JAK3 inhibitors for the treatment of leukemia harboring a JAK3 activating mutant. J Med Chem. 2022;65: 10674–10690.

77. Si H, Wang J, He R, Yu X, Li S, Huang J, et al. Identification of U937JAK3-M511I acute myeloid leukemia cells as a sensitive model to JAK3 inhibitor. Front Oncol. 2022;11. doi:10.3389/fonc.2021.807200

78. Yim J, Koh J, Kim S, Song SG, Bae JM, Yun H, et al. Clinicopathologic and genetic features of primary T-cell lymphomas of the central nervous system. Am J Surg Pathol. 2022;46: 486–497.

79. Warsi J, Elvira B, Hosseinzadeh Z, Shumilina E, Lang F. Downregulation of chloride channel ClC-2 by Janus kinase 3. J Membr Biol. 2014;247: 387–393.

80. Warsi J, Luo D, Elvira B, Jilani K, Shumilina E, Hosseinzadeh Z, et al. Upregulation of excitatory amino acid transporters by coexpression of Janus kinase 3. J Membr Biol. 2014;247: 713–720.

81. Rivera-Munoz P, Laurent AP, Siret A, Lopez CK, Ignacimouttou C, Cornejo MG, et al. Partial trisomy 21 contributes to T-cell malignancies induced by JAK3-activating mutations in murine models. Blood Adv. 2018;2: 1616–1627.

82. Basheer F, Bulleeraz V, Ngo VQT, Liongue C, Ward AC. In vivo impact of JAK3 A573V mutation revealed using zebrafish. Cell Mol Life Sci. 2022;79. doi:10.1007/s00018-022-04361-8

83. Agarwal A, MacKenzie RJ, Eide CA, Davare MA, Watanabe-Smith K, Tognon CE, et al. Functional RNAi screen targeting cytokine and growth factor receptors reveals oncorequisite role for interleukin-2 gamma receptor in JAK3-mutation-positive leukemia. Oncogene. 2015;34: 2991–2999.

84. Bouchekioua A, Scourzic L, de Wever O, Zhang Y, Cervera P, Aline-Fardin A, et al. JAK3 deregulation by activating mutations confers invasive growth advantage in extranodal nasal-type natural killer cell lymphoma. Leukemia. 2014;28: 338–348.

85. Sim SH, Kim S, Kim TM, Jeon YK, Nam SJ, Ahn Y-O, et al. Novel JAK3-activating mutations in extranodal NK/T-cell lymphoma, nasal type. Am J Pathol. 2017;187: 980–986.

86. Koo GC, Tan SY, Tang T, Poon SL, Allen GE, Tan L, et al. Janus kinase 3–activating mutations identified in natural killer/T-cell lymphoma. Cancer Discov. 2012;2: 591–597.

87. Steven Martinez G, A. Ross J, A. Kirken R. Transforming mutations of Jak3 (A573V and M511I) show differential sensitivity to selective Jak3 inhibitors. Clin Cancer Drugs. 2016;3: 131–137.

88. Bergmann AK, Schneppenheim S, Seifert M, Betts MJ, Haake A, Lopez C, et al. Recurrent mutation of *JAK3* in T‐cell prolymphocytic leukemia. Genes Chromosomes Cancer. 2014;53: 309–316.

89. Sato T, Toki T, Kanezaki R, Xu G, Terui K, Kanegane H, et al. Functional analysis of *JAK3* mutations in transient myeloproliferative disorder and acute megakaryoblastic leukaemia accompanying Down syndrome. Br J Haematol. 2008;141: 681–688.

90. Xu L, Wilson RA, Laetsch TW, Oliver D, Spunt SL, Hawkins DS, et al. Potential pitfalls of mass spectrometry to uncover mutations in childhood soft tissue sarcoma: A report from the Children’s Oncology Group. Sci Rep. 2016;6. doi:10.1038/srep33429

91. de Martino M, Gigante M, Cormio L, Prattichizzo C, Cavalcanti E, Gigante M, et al. JAK3 in clear cell renal cell carcinoma: Mutational screening and clinical implications. Urol Oncol. 2013;31: 930–937.

92. Ehrentraut S, Schneider B, Nagel S, Pommerenke C, Quentmeier H, Geffers R, et al. Th17 cytokine differentiation and loss of plasticity after SOCS1 inactivation in a cutaneous T-cell lymphoma. Oncotarget. 2016;7: 34201–34216.

93. Alghamdi K. Delayed diagnosis of a hyper functioning parathyroid cyst. A case report and genetic analysis. Acta Endocrinol (Buchar). 2016;12: 215–218.

94. Yin C, Sandoval C, Baeg G-H. Identification of mutant alleles of*JAK3*in pediatric patients with acute lymphoblastic leukemia. Leuk Lymphoma. 2015;56: 1502–1506.

95. Elli EM, Mauri M, D’Aliberti D, Crespiatico I, Fontana D, Redaelli S, et al. Idiopathic erythrocytosis: a germline disease? Clin Exp Med. 2024;24. doi:10.1007/s10238-023-01283-y

96. Sic H, Speletas M, Cornacchione V, Seidl M, Beibel M, Linghu B, et al. An activating Janus kinase-3 mutation is associated with cytotoxic T lymphocyte antigen-4-dependent immune dysregulation syndrome. Front Immunol. 2017;8. doi:10.3389/fimmu.2017.01824

97. Motegi T, Kochi Y, Matsuda K, Kubo M, Yamamoto K, Momozawa Y. Identification of rare coding variants in *TYK2* protective for rheumatoid arthritis in the Japanese population and their effects on cytokine signalling. Ann Rheum Dis. 2019;78: 1062–1069.

98. Nemoto M, Hattori H, Maeda N, Akita N, Muramatsu H, Moritani S, et al. Compound heterozygous TYK2 mutations underlie primary immunodeficiency with T-cell lymphopenia. Sci Rep. 2018;8. doi:10.1038/s41598-018-25260-8

99. Li Z, Rotival M, Patin E, Michel F, Pellegrini S. Two common disease-associated TYK2 variants impact exon splicing and TYK2 dosage. PLoS One. 2020;15: e0225289.

100. Kerner G, Ramirez-Alejo N, Seeleuthner Y, Yang R, Ogishi M, Cobat A, et al. Homozygosity for *TYK2* P1104A underlies tuberculosis in about 1% of patients in a cohort of European ancestry. Proc Natl Acad Sci U S A. 2019;116: 10430–10434.

101. López-Isac E, Campillo-Davo D, Bossini-Castillo L, Guerra SG, Assassi S, Simeón CP, et al. Influence of*TYK2*in systemic sclerosis susceptibility: a new*locus*in the IL-12 pathway. Ann Rheum Dis. 2016;75: 1521–1526.

102. Ogishi M, Arias AA, Yang R, Han JE, Zhang P, Rinchai D, et al. Impaired IL-23–dependent induction of IFN-γ underlies mycobacterial disease in patients with inherited TYK2 deficiency. J Exp Med. 2022;219. doi:10.1084/jem.20220094

103. Li Z, Gakovic M, Ragimbeau J, Eloranta M-L, Rönnblom L, Michel F, et al. Two rare disease-associated Tyk2 variants are catalytically impaired but signaling competent. J Immunol. 2013;190: 2335–2344.

104. Ehm MG, Aponte JL, Chiano MN, Yerges-Armstrong LM, Johnson T, Barker JN, et al. Phenome-wide association study using research participants’ self-reported data provides insight into the Th17 and IL-17 pathway. PLoS One. 2017;12: e0186405.

105. Enerbäck C, Sandin C, Lambert S, Zawistowski M, Stuart PE, Verma D, et al. The psoriasis-protective TYK2 I684S variant impairs IL-12 stimulated pSTAT4 response in skin-homing CD4+ and CD8+ memory T-cells. Sci Rep. 2018;8. doi:10.1038/s41598-018-25282-2

106. Diogo D, Bastarache L, Liao KP, Graham RR, Fulton RS, Greenberg JD, et al. TYK2 protein-coding variants protect against rheumatoid arthritis and autoimmunity, with no evidence of major pleiotropic effects on non-autoimmune complex traits. PLoS One. 2015;10: e0122271.

107. Woess K, Macho-Maschler S, Van Ingen Schenau DS, Butler M, Lassnig C, Valcanover D, et al. Oncogenic TYK2 P760L kinase is effectively targeted by combinatorial TYK2, mTOR and CDK4/6 kinase blockade. Haematologica. 2022;108: 993–1005.

108. Waanders E, Scheijen B, Jongmans MCJ, Venselaar H, van Reijmersdal SV, van Dijk AHA, et al. Germline activating TYK2 mutations in pediatric patients with two primary acute lymphoblastic leukemia occurrences. Leukemia. 2017;31: 821–828.

109. Jensen LT, Attfield KE, Feldmann M, Fugger L. Allosteric TYK2 inhibition: redefining autoimmune disease therapy beyond JAK1-3 inhibitors. EBioMedicine. 2023;97: 104840.

110. Kerner G, Laval G, Patin E, Boisson-Dupuis S, Abel L, Casanova J-L, et al. Human ancient DNA analyses reveal the high burden of tuberculosis in Europeans over the last 2,000 years. Am J Hum Genet. 2021;108: 517–524.
